# Reversible Dissociation of a Dialumene**

**DOI:** 10.1002/anie.202111385

**Published:** 2021-10-07

**Authors:** Rosalyn L. Falconer, Keelan M. Byrne, Gary S. Nichol, Tobias Krämer, Michael J. Cowley

**Affiliations:** ^1^ School of Chemistry University of Edinburgh Edinburgh UK; ^2^ Department of Chemistry Maynooth University Maynooth Co. Kildare Ireland

**Keywords:** aluminium, aluminium(I) compounds, dialumene, low-valent atoms, multiple bonds

## Abstract

Dialumenes are neutral Al^I^ compounds with Al=Al multiple bonds. We report the isolation of an amidophosphine‐supported dialumene. Our X‐ray crystallographic, spectroscopic, and computational DFT analyses reveal a long and extreme trans‐bent Al=Al bond with a low dissociation energy and bond order. In solution, the dialumene can dissociate into monomeric Al^I^ species. Reactivity studies reveal two modes of reaction: as dialumene or as aluminyl monomers.

## Graphical abstract

A highly trans‐bent dialumene has marginal Al=Al double‐bond character and, in solution, can dissociate into monomeric aluminyl fragments. Reactivity studies reveal that both the dialuminene and aluminyl monomer can be trapped by varying the reaction partner. DFT calculations elaborate the origins of the extreme trans‐bending, weak Al=Al bond, and define substituent effects in dialumenes.

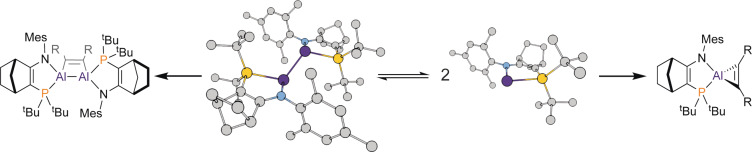

## Introduction

Like other low oxidation‐state main group systems, Al^I^ compounds are revealing potential in bond‐activation and catalysis.^[1]^ Dialumenes are neutral Al^I^ compounds with Al=Al multiple bonds. They sit alongside the prototypical neutral Al^I^ compounds (Cp*Al)_4_ and NacNacAl(I), and the rapidly developing class of anionic aluminyl compounds.^[2]^

**Figure 1:**
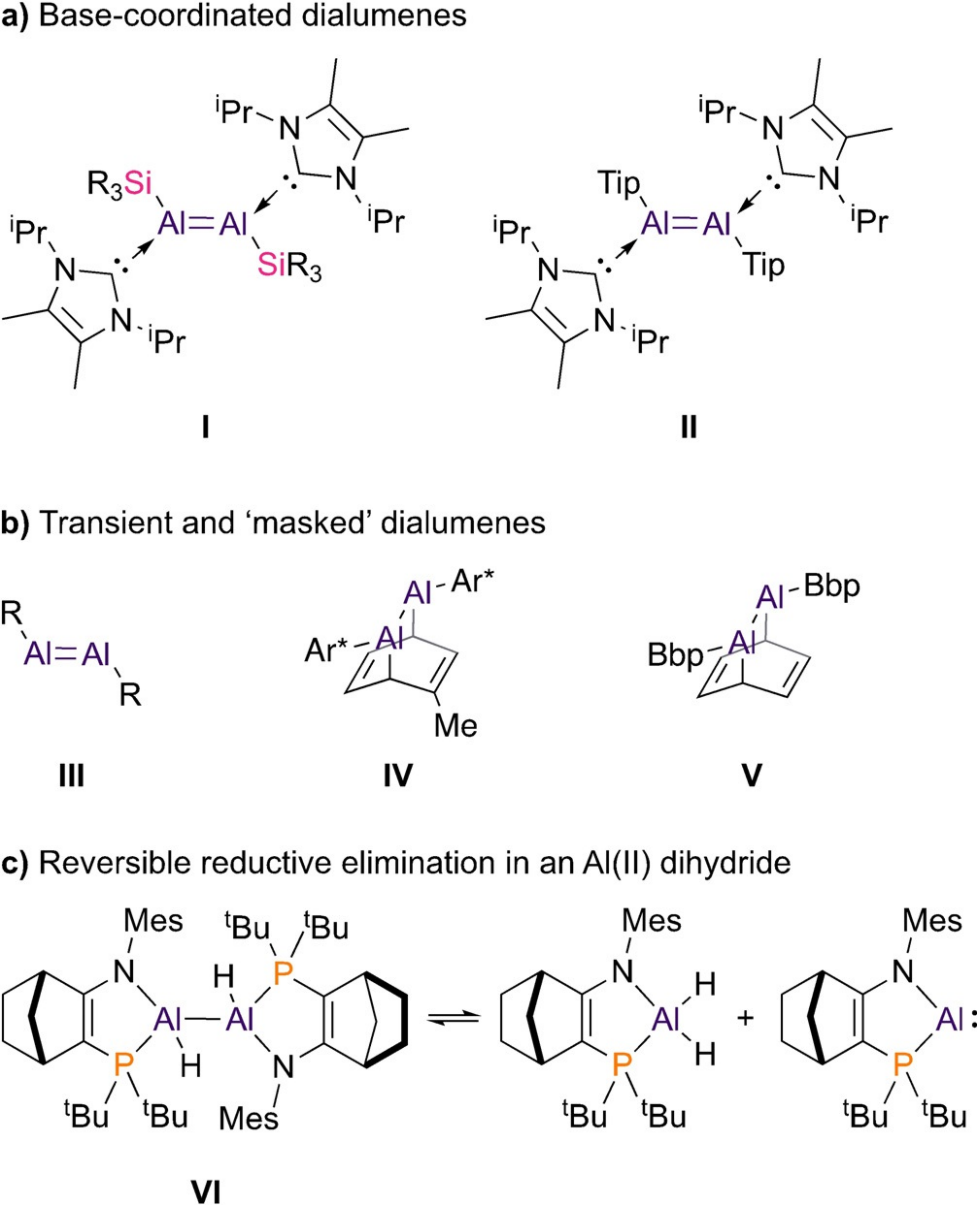
a) Base‐coordinated dialumenes (R_3_Si=Me^t^Bu_2_Si; Tip=2,4,6‐triisopropylphenyl). b) “masked” dialumenes (Ar*=2,6‐(2,6‐diisopropylphenyl)phenyl; Bbp=2,6‐(bis(trimethylsilyl)methyl)phenyl). c) Reversible reductive elimination in **VI** (Mes=2,4,6‐trimethylphenyl).

Dialumenes can be divided into two classes: base‐coordinated dialumenes (R(L)Al=Al(L)R), which are isoelectronic with alkenes, and “transient” dialumenes (RAl=AlR). Two base‐coordinated dialumenes have been reported. The first, silyl substituted **I**, was reported by Inoue in 2017.^[3a]^ An aryl analogue, **II**, followed (Figure 1).^[3b]^ Though base‐free dialumenes (**III**) are yet to be isolated, “masked” examples that behave as RAl=AlR are known. Power reported the toluene adduct **IV**,^[4]^ and Tokitoh the related benzene adduct **V**.^[5]^

Dialumenes readily activate dihydrogen and other small molecules.[^5^, ^6^] Inoue's **I** and **II** catalytically reduce CO_2_ with HBPin.[^3b^, ^7^] This capability comes from closely‐spaced frontier molecular orbitals, which beget high reactivity. Even considering the only isolated examples, **I** and **II**, it is clear that understanding the interplay between substituents, bonding, and reactivity in dialumenes is critical to their further development.

Base‐coordinated and base‐free dialumenes are clearly related, but insights from experiment and theory reveal very different pictures of bonding. Dialumenes **I** and **II** feature planar or moderately trans‐bent Al=Al bonds with double bond character, do not dissociate, and react as dialumenes. In contrast, donor‐free dialumenes **III** feature low Al=Al bond orders and substantially trans‐bent geometry.^[8]^ These dialumenes can dissociate readily in solution; **V** appears to react as either RAl=AlR or RAl: species.^[9]^ Recently, Power showed that a larger terphenyl substituent allows access to an RAl: monomer rather than **IV**.^[10]^

A transient N,P‐coordinated aluminyl monomer was implicated in our recent studies of reductive elimination in the Al(II) dihydrodialane **VI** (Figure 1 c).^[11]^ We thus targeted isolable Al^I^ compounds of the same amidophosphine ligand.

We report here the base‐coordinated dialumene **1**. Our studies demonstrate that **1** has an unusually weak Al=Al bond with low bond‐order and an extreme trans‐bent geometry. We reveal how the amidophosphine ligand of **1** is the origin of these effects. In solution, **1** dissociates and can react as either dialumene or monomeric aluminyl.

## Results and Discussion

**Scheme 1:**
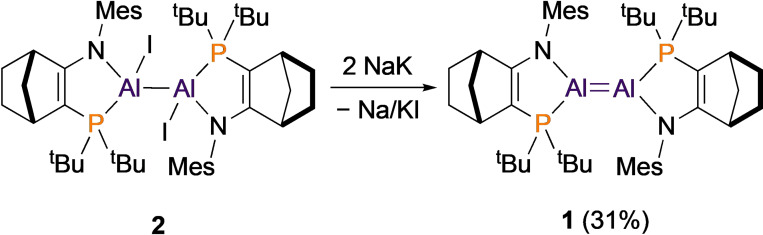
Preparation of dialumene **1**.

We prepared dialumene **1** by reduction from the Al(II) precursor diiododialane **2** (Scheme 1). Treatment of **2** with 2 equiv Na/K alloy in THF led to a colour change from yellow to dark purple. After 5 hours, ^31^P{^1^H} NMR spectroscopy revealed consumption of **2** and a new broad resonance at *δ* 21.3, as well as minor amounts of dihydrodialane **VI**. Crystalline dialumene **1** was isolated as a dark purple solid in 31 % yield from toluene at −30 °C. UV/vis spectroscopy revealed *λ*_max_ 567.0 nm, which we assign to a π to π* transition (Figure S3, Table S10). At 293 K, **1** decomposes over 1–2 days in THF, toluene or hexane solutions.

**Figure 2:**
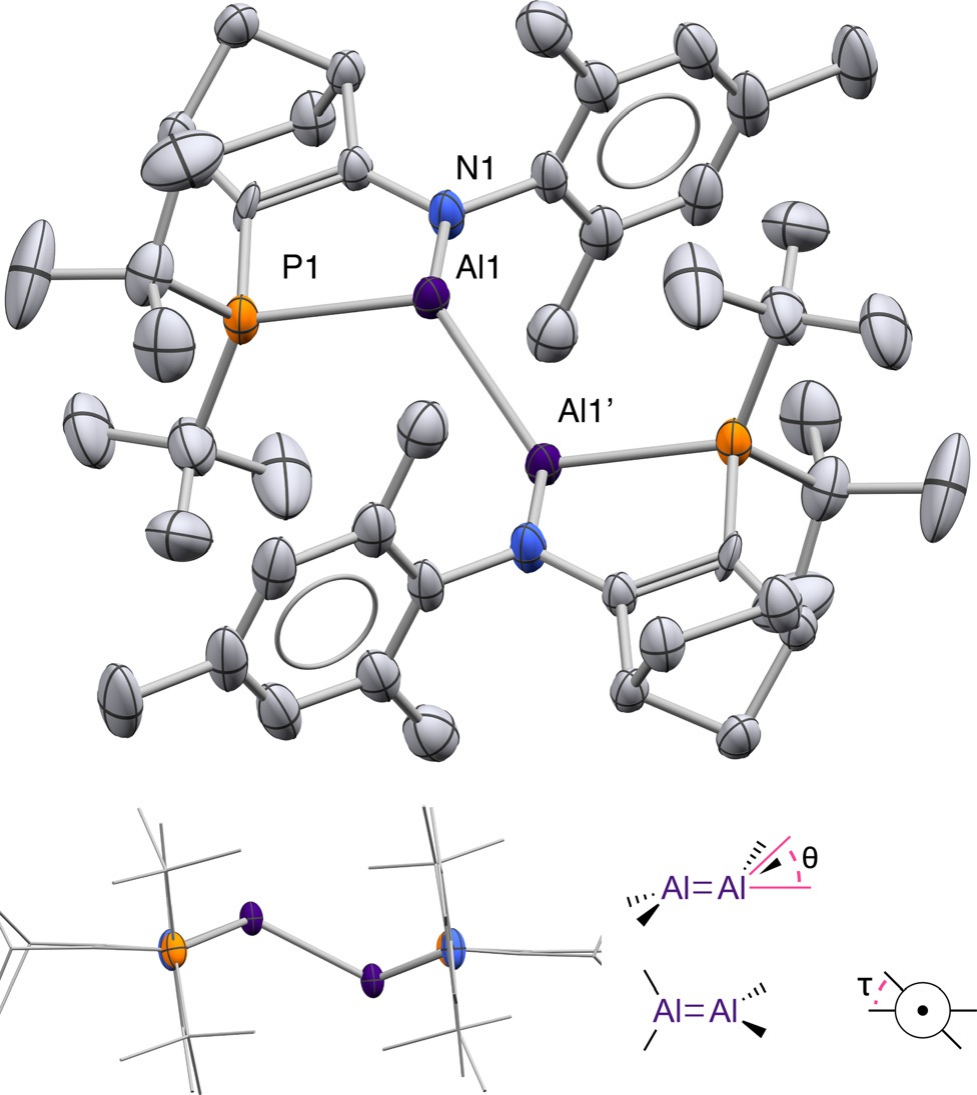
X‐ray crystal structure of dialumene **1** (H atoms omitted for clarity). Thermal ellipsoids at 50 % probability. Major component of disordered Al/ligand displayed (**1A**). Selected bond distances [Å] and angles [°]: Al1‐Al1′ 2.5190(14); N1‐Al1 1.909(2); P1‐Al1 2.4816(9); N1‐Al1‐P1 84.86(7); *θ*=48.8; *τ*=0.^[21]^

The solid‐state structure of dialumene **1**, determined by X‐ray crystallography, reveals a highly trans‐bent Al=Al bond in *E* configuration (Figure 2). Two‐site disorder of the Al positions reveals major and minor isomers of **1** (88/12 %) with distinct geometries around the Al_2_ core. The amidophosphine ligands enforce narrow N1−Al1−P1 angles (83–84°). The Al=Al distance in **1** is shorter by 0.1–0.2 Å than in the related Al(II) dihydrodialane **VI** or in Uhl's dialane(4) ((SiMe_3_)_2_HC)_2_Al−Al(CH(SiMe_3_)_2_)_2_ (**1** 2.5190(14)/2.471(13) Å; **VI** 2.6586(16) Å; Uhl's dialane 2.660(1) Å).^[12]^ Nevertheless, the Al=Al distance in **1** is notably longer (∼0.1 Å) than in Inoue's dialumenes [**I** 2.3943(16); **II** 2.4039(8)]. Compared to **I** and **II**, the Al=Al core of **1** is much less planar (**1** *θ*=48.8°/51.2°; **I**: 0°; **II**: 17.3°/23.7°). We note that the pyramidalised Al centres in **1** are stereogenic; the major and minor isomers in the solid‐state structure have opposite stereochemistry at the Al centres.

**Figure 3:**
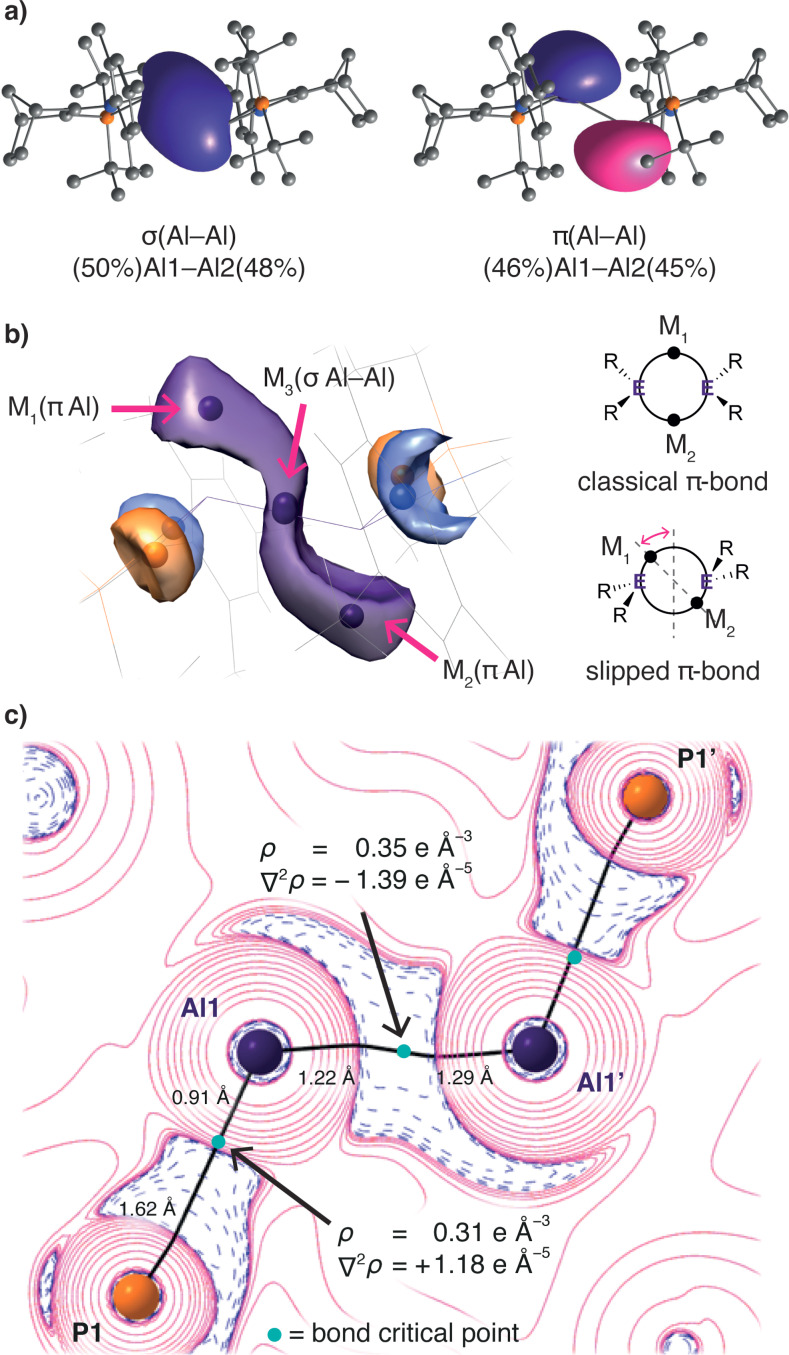
Electronic structure analysis of **1**. a) NLMOs (isovalue= 0.036) of the Al=Al bond. b) ELF localisation domains (isovalue= 0.795) of the Al=Al core. M_*n*_ indicates attractors. c) Laplacian of the electron density in the P−Al=Al−P plane. Areas of charge concentration and depletion (blue/pink), bond paths (black) and bond critical points shown.

DFT calculations reveal that the bonding situation in **1** is distinct from previous base‐coordinated dialumenes **I** and **II**. Natural Bond Orbital (NBO) analysis of **1** shows natural localised molecular orbitals (NLMOs) representing Al−Al σ‐ and π‐bonds (Figure 3 a). Although it retains some apparent π‐bond character, the corresponding NLMO of **1** is heavily localised on the aluminium centres; the relevant NLMOs of **I** or **II** more closely resemble classical π‐orbitals (Figure S19/20). The localisation of the π‐orbital in dialumene **1** results from admixture of the Al−Al σ*. The extent of this admixture is revealed by the increased s‐character of the NLMO of **1** (Al1/Al2 sp^1.25^/sp^1.09^) compared to that in, for example, **II** (Al1/Al2 sp^48.34^/sp^23.32^), where the π‐bond is constructed from essentially pure p‐orbitals. Consistent with this picture, the Wiberg bond index for the Al=Al bond of **1** is 1.31, which is increased from that of dihydrodialane **VI** (0.91) but lower than in **I** or **II** (1.67, 1.54).

Grützmacher and Fässler have proposed simple topographical criteria for distinguishing classical and non‐classical multiple bonds.^[13]^ Their criteria use the Electron Localisation Function (ELF), which identifies regions of localised valence electron density. The ELF of classically π‐bonded systems reveals “attractors”—local maxima *M* in the ELF that correspond to electron pairs—above and below the plane of E=E bonds. Each attractor *M* is surrounded by a “basin” of electron density. The topology and electron population of such basins is interpretable in familiar terms as covalent bonds or lone pairs. Classical π‐bonded systems have “dumb‐bell” shaped electron basins, and their populations sum to approximately 4 e^−^.

Topological analysis of the ELF of **1** (Figure 3 b) reveals a quite different picture. The characteristic pattern of attractors and basins for a slipped π‐bond is observed. Three valence attractors, M_1_‐M_3_, are found near the Al_2_ unit. M_3_ is centred on the Al−Al bond; its basin population is 1.11 e^−^. M_1_ and M_2_ are each above or below an Al centre, “slipped” from their positions in a classical double bond. The basins of M_1_/M_2_ are each populated by ∼1.30 e^−^; the summed basin populations (3.76 e^−^) correspond with the 4 e^−^ available for bonding from the two Al^I^ centres of **1**.

The positions of attractors M_1_‐M_3_ in **1** correspond with those in the base‐free dialumene Al_2_H_2_.[^8a^, ^14^] The sigmoidal form of the ELF isosurface of **1** is a feature of bonding in base‐free dialumenes, along with low Al−Al bond order (∼0.5) and diradical character.^[8a]^ Based on the basin population around M_3_ (1.11 e^−^), **1** also has low Al−Al bond order. M_1_ and M_2_ can be interpreted as non‐bonding electron density at Al. In contrast, the ELF of planar dialumene **I** reveals features of classical π‐bonds: an attractor on each face of the Al=Al bond (Figure S23).

To better determine the Al−Al bond order in **1**, we undertook Quantum Theory of Atoms in Molecules (QTAIM) analysis (Figure 3 c and SI). The molecular graph reveals Al−Al, Al−N and Al−P bond paths. The Al−P interactions are polar dative bonds, as revealed by the position of their bond critical points (bcp) closer to the more electropositive Al centres and associated QTAIM parameters (*σ*_bcp_=0.310 e Å^−3^, ∇^2^ *σ*_bcp_=+1.180 e Å^−5^, *H*_bcp_=−0.107 *E*_h_ Å^−3^), *G*_bcp_/*σ*_bcp_<1).^[15]^ Meanwhile the Al−N bonds exhibit stronger ionic character (*σ*_bcp_=0.499 e Å^−3^, ∇^2^ *σ*_bcp_=+8.410 e Å^−5^, *H*_bcp_=−0.094 *E*_h_ Å^−3^, *G*_bcp_/*σ*_bcp_<1). The Al−Al bond features weak shared‐shell covalent character, as judged by the charge concentration and topological parameters at its bcp (*σ*_bcp_=0.349 e Å^−3^, ∇^2^ *σ*_bcp_=−1.390 e Å^−5^, *H*_bcp_=−0.135 *E*_h_ Å^−3^, *G*_bcp_/*σ*_bcp_<1). In accordance with the ELF results, the values of both *σ*_bcp_ and ∇^2^ *σ*_bcp_ are rather low, indicating a weak Al−Al bond. The bond ellipticity parameter suggests a small degree of double bond character (*ϵ*_bcp_=0.195).

The delocalisation index *δ*(A,B) is a quantitative measure for the number of electron pairs exchanged between two atomic basins. When referenced against a chemically‐similar comparator compound with a well‐defined bonding situation, the delocalisation index can reflect chemical bond order. Here, we use *δ*(Al,Al) of the bond in dihydrodialane **VI** to define an Al−Al bond order of 1. At 0.65, *δ*(Al,Al) in **VI** is about half that in the planar transition state **TS_1C‐1C_** (see later) which unequivocally has a planar Al=Al double bond (*δ*(Al,Al)=1.21). In trans‐bent dialumene **1**, *δ*(Al,Al) at 0.80 is only slightly higher than that of dihydrodialane **VI**, but much lower than that of the Al=Al double‐bond.^[14]^

The combined results of our crystallographic and electronic structure analyses indicate small but significant Al=Al multiple bond character in **1**. Al−Al bond distance, and computational bond order and delocalisation‐index criteria all support the conclusion that the Al=Al bond in **1** is intermediate between single and double bonds, with bond order ∼1.3.

Why is dialumene **1** so different from **I** and **II**? We used DFT calculations on a set of minimal base‐coordinated dialumenes with NHC or PMe_3_ donors and hydride, phenyl, silyl or amino substituents (Table 1) to answer this question.^[16]^

**Table 1:** Selected geometrical and thermodynamic properties of model dialumenes calculated at SMD‐B3LYP‐D3/6‐311G(2d,2p)//M062X‐D3/def2SVP level^[a]^

	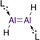	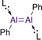	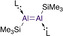	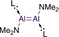	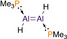	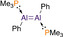	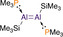	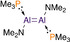
Al=Al [Å]	2.42	2.44	2.39	2.48	2.45	2.47	2.46	2.60
L‐Al‐R [°]	101.7	97.1	112.1/106.9	91.9	95.8	92.7	98.7	99.4
*θ* [°] ^[b]^	29.6/47.2^[d]^	33.4/53.9^[e]^	19.1/16.3^[f]^	43.1	46.6	50.5/50.4	41.6/41.0	63.5/44.6^[g]^
Δ*G*_298_(dissoc) [kcal mol^−1^]^[c]^	22.1	20.6	33.2	11.5	19.3	19.7	25.5	2.1
Δ*E*_S‐T_(monomer) [kcal mol^−1^]	19.2	20.6	12.3	25.5	23.2	25.4	16.4	32.4

[a] L=NHC, Imidazol‐2‐ylidene (C_3_H_4_N_2_). [b] *θ*=trans‐bend angle, see Figure 2. Unless otherwise noted, *τ*=0°. Where two values are listed, complexes are unsymmetrically trans‐bent. [c] corrected for basis set superposition error (Table S7). [d] *τ*=17.8°. [e] *τ*=20.5°. [f] *τ*=7.9°. [g] *τ*=19.8°.

The structures of the model dialumenes depend strongly on the substituent and Lewis base (NHC or PMe_3_). Electropositive substituents (SiMe_3_) provoke shorter Al=Al bonds, wider R−Al−L angles, and more planar structures. More electronegative (Si < H < Ph < N) or π‐donating substituents induce more trans‐bending and longer Al=Al bonds. NHC‐coordinated dialumenes always have shorter and more planar Al=Al bonds than their PMe_3_ counterparts (Al=Al=2.42–2.48 Å vs. 2.45–2.60 Å).

These substituent effects mimic those in disilenes, reflecting the isoelectronic relationship between R_2_Si=SiR_2_ and R(L)Al=Al(L)R. In disilenes, trans‐bend angles and Si=Si bond distances are correlated with the singlet‐triplet energy gap (Δ*E*_S‐T_) of the notional or real silylene monomers, :SiR_2_.^[17]^ We find that the same relationship applies to dialumenes: Al=Al bond dissociation energy increases as Δ*E*_S‐T_ for the monomeric R(L)Al: fragments decreases (Figure S11). The result is that dialumene bond dissociation energy/geometry can be predicted based on properties of the R(L)Al: (aluminyl) monomer.

We attribute the stronger and more planar Al=Al bonds of NHC‐ vs. PMe_3_‐coordinated dialumenes to the strong donor ability of the NHC, which raises the R(L)Al: HOMO, narrowing Δ*E*_S‐T_. In contrast, the low dissociation energy for Me_2_N(PMe_3_)Al=Al(PMe_3_)NMe_2_, (2.1 kcal mol^−1^) is explained by the large Δ*E*_S‐T_ for the Me_2_N(Me_3_P)Al: fragment (32.4 kcal mol^−1^).

Returning to dialumene **1**, we can ascribe its extreme trans‐bending to the electronegative/π‐donating NR_2_ substituent and narrow 85° N1‐Al1‐P1 angle enforced by the ligand, which both increase Δ*E*_S‐T_ in the monomeric aluminyl fragment (Table S9). Calculations on the full dialumene **1** predict a bond dissociation energy of 7.1 kcal mol^−1^, vs. 25.0 and 19.0 kcal mol^−1^ for **I** and **II** (Table S7). To explore the possible dissociation of **1**, we turned to its solution‐phase behaviour.

**Figure 4:**
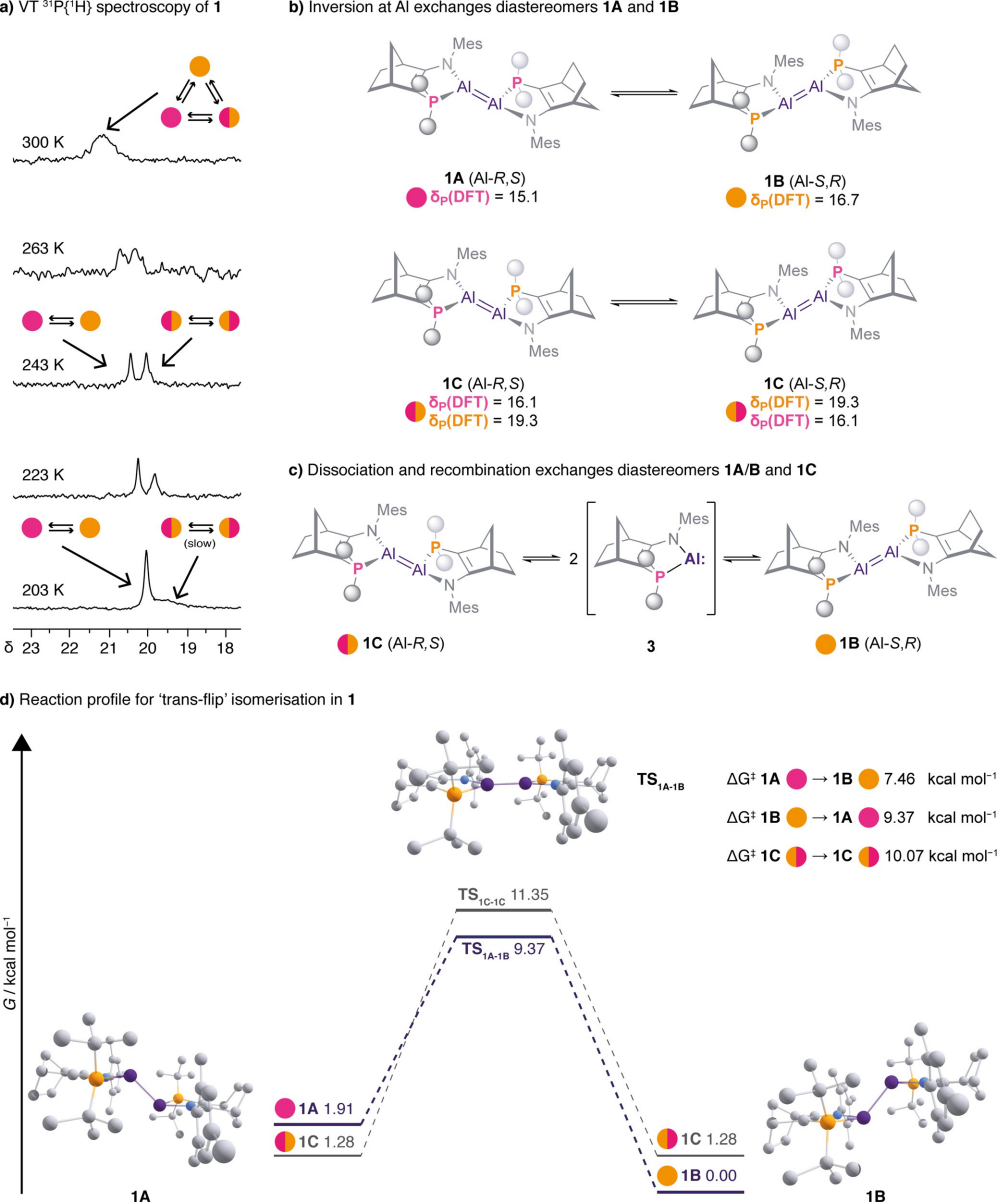
a) ^31^P{^1^H} NMR spectra of **1** (161 MHz, [D_8_]toluene) recorded at 203–300 K. b) Inversion at aluminium exchanges **1A** and **1B**, but is degenerate for **1C**. c) Intermolecular dissociation/recombination of **1** exchanges all diastereomers. d) Reaction energy profile for the “trans‐flip” in diastereomers **1A‐C** at *T*=298.15 K (geometries optimised at M062X‐D3/def2SVP, energies calculated at B3LYP‐D3/6‐311G(2d,2p) corrected for C_6_H_6_ solvent).

Dialumene **1** is predominantly dimeric in solution. Its ^31^P{^1^H} NMR spectrum at 300 K has one broad signal at *δ* 21.3 (Δ*ν*_1/2_=134 Hz) (Figure 4 a). ^1^H NMR spectroscopy reveals two ligand environments for **1**, in the ratio 54 %:46 %, indicating at least two (stereo)isomers. The stereogenic Al centres of **1**, in combination with its ligand backbone, mean that there are three possible diastereomers of *E*‐**1**, **A‐C** (Figures 4 a, S1), each of which must have distinct ^31^P signals.^[18]^ **1A** and **1B** are *meso* compounds with equivalent phosphorus centres—each will give rise to a singlet. **1C** has inequivalent phosphorus centres, so two ^31^P resonances (potentially doublets with ^3^ *J*_PP_). The pattern of DFT‐predicted ^31^P signals confirms our stereochemical analysis (Figure 4 c, S15).

At 300 K, the broad ^31^P{^1^H} resonance at *δ* 21.3 indicates diastereomers **1A‐C** are exchanging. Cooling to 243 K, resolves this broad signal into two singlets (*δ* 20.4 and *δ* 20.0). At 203 K, the higher field signal (*δ* 19.5) broadens and approaches coalescence (Δ*ν*_1/2_=148 Hz).

The dynamic ^31^P{^1^H} NMR behaviour of **1** arises from a combination of intra‐ and intermolecular exchange processes that exchange diastereomers **1A**–**C**. In the low temperature regime (≲300 K), only intramolecular fluxional processes are operative. The two singlets observed at 243 K are assigned to **1A**/**B** and **1C**. A “trans‐flip” process, fast on the NMR timescale at this temperature, simultaneously inverts the stereochemistry at both aluminium centres (Figure 4 b). This has the effect of interconverting diastereomers **1A** and **1B**, generating a (concentration‐weighted) time‐averaged signal for them. In **1C**, the trans‐flip is instead a degenerate process that exchanges the two inequivalent phosphorus centres, leading to the observed singlet. At 203 K, we assign the broad signal to **1C**, in which the trans‐flip is becoming slow on the NMR timescale.

Using DFT calculations we were able to locate the planar transition states **TS_1A‐1B_** and **TS_1C‐1C_** for the trans‐flip process (Figure 4 d). The barriers for this process range from 8 to 11 kcal mol^−1^. **TS_1C‐1C_** is higher in energy than **TS_1A‐1B_** (11.35 vs. 9.37 kcal mol^−1^).

In the higher temperature regime (∼300 K), exchange between isomers **1A**/**B** and **1C** becomes active through an intermolecular route. Dissociation of dialumene **1** generates monomeric aluminyl **3**, which can then recombine to form any of the three diastereomers of **1** (Figure 4 c). This process is possible due to the low dissociation energy of **1**, (DFT predicts Δ*G*_298_=+7.1 kcal mol^−1^). 2D ^1^H EXSY NMR spectroscopy at 300 K reveals exchange cross peaks between resonances for **1A**/**1B** (time averaged) and **1C** (Figures S6/7). Our DFT calculations place diastereomers **1A**–**C** very close in energy, spanning just 2 kcal mol^−1^. Experimental measurements are consistent with this. We were able to determine the equilibrium constants for the exchange of [**1A**+**1B**] with **1C** in the temperature range 188–243 K (Figure S5). We can thus estimate Δ*G* ^0^ for [**1A**+**1B**] → **1C** as 0.8±0.2 kJ mol^−1^ (0.19±0.04 kcal mol^−1^).

The presence of aluminyl **3** in solution is revealed by dynamic NMR behaviour, but its concentration must be rather low since we did not observe signals for it. Nor did UV‐vis spectroscopy in the temperature range 5–65 °C reveal absorptions for **3** (Figure S3). Lacking direct spectroscopic evidence, we sought to trap **3**.

**Scheme 2:**
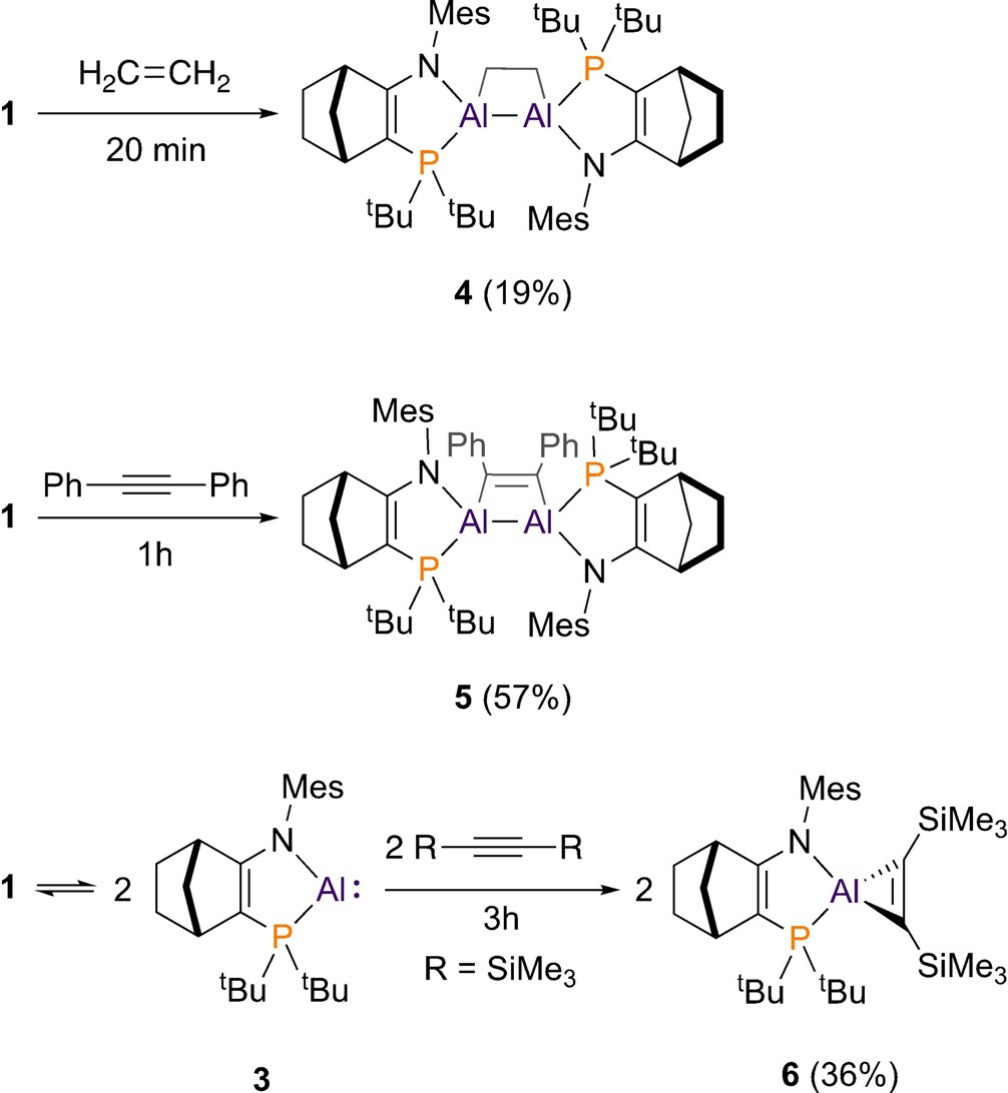
Reactivity of dialumene **1** with alkenes/alkynes.

Like **I** and **II**,^[3]^ **1** can react with alkenes and alkynes to form 4‐membered aluminacycles. Treatment of 1 with ethene (1 atm) at room temperature results in rapid (5–20 mins) conversion to dialuminacyclobutane **4** by formal [2+2] cycloaddition of the Al=Al and C=C bonds. Similarly, diphenylacetylene reacts with **1** to form dialuminacyclobutene **5** (Scheme 2). ^31^P{^1^H} NMR spectroscopy of **4** and **5** reveals distinct signals for three diastereomers in each case. This is a result of the “locking” of the stereogenic aluminium centres enforced by their cyclic structures (**4**: *δ* 11.6 (d, ^3^ *J*_PP_=12 Hz), 11.5 (s), 11.5 (s), 11.4 (d, ^3^ *J*_PP_=12 Hz);, **5**: *δ* 11.0 (br s), 10.7 (s), 10.4 (s), 10.3 (br s).; see SI).

X‐ray crystallography reveals the geometry of the C_2_Al_2_ rings of **4** and **5**. The Al−Al distances in **4** and **5** are not notably longer than in **1**, despite destruction of the Al=Al π bond (**1**, 2.519(1) Å; **4,** 2.558(1) Å; **5**, 2.512(1) Å, see SI). This is rather different to the behaviour of dialumenes **I** or **II** in comparable reactions with alkenes/alkynes. The resulting analogues of **4**/**5** exhibit substantial Al−Al bond elongation (0.20–0.25 Å) compared to **I**/**II**. The difference reflects the lower Al=Al bond order in **1** vs. **I**/**II**.

When dialumene **1** is treated with the bulkier alkyne Me_3_SiC≡CSiMe_3_, the observed product is derived not from **1** but rather from its monomer, **3**. On addition of Me_3_SiC≡CSiMe_3_, purple solutions of **1** become yellow within three hours. ^31^P{^1^H} NMR reveals a broad signal at *δ* 9.8, characteristic of amidophosphine‐coordinated Al(III) compounds.^[19]^

**Figure 5:**
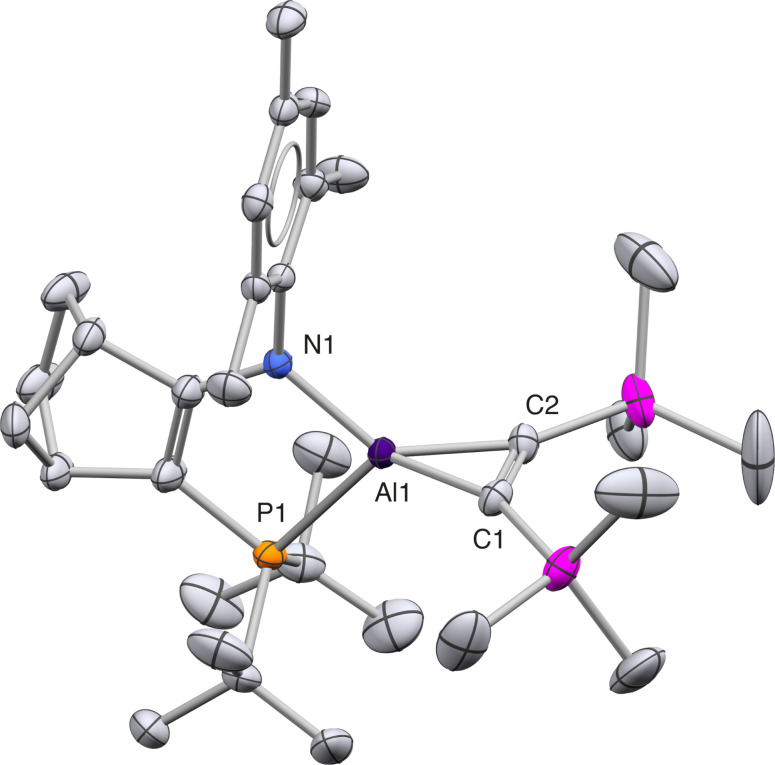
X‐ray crystal structure of aluminacyclopropene **6** (H atoms omitted). Thermal ellipsoids at 50 % probability. The asymmetric unit comprises two molecules; just one is shown.

X‐ray crystallography shows that the product from **1** and Me_3_SiC≡CSiMe_3_ is aluminacyclopropene **6** (Figure 5). **6** has the narrow C1−Al−C2 angle expected for aluminacyclopropenes (42.05(9)°) and its C1=C2 distance is typical for a double bond (1.367(2) Å). Cycloaddition reactions with alkynes are a characteristic reaction for neutral aluminyls. A NacNac‐coordinated analogue of **6** has been prepared by reduction of Al(III) precursors in the presence of Me_3_SiC≡CSiMe_3_, though with other alkynes direct reaction with NacNacAl(I) is also viable.^[20]^ Structurally, the AlC_2_ core of **5** and its NacNac analogue are closely comparable.

## Conclusion

In summary, we have prepared the first isolable dialumene that dissociates in solution. The donor properties of the amidophosphine ligand generate a large Δ*E*_S‐T_ on the transient aluminyl monomers. This large Δ*E*_S‐T_ is the origin of the low bond order, high trans‐bending, and weak Al=Al bond in **1**. We continue to explore the reactivity of **1** and related systems.

## Conflict of interest

The authors declare no conflict of interest.

## Supporting information

As a service to our authors and readers, this journal provides supporting information supplied by the authors. Such materials are peer reviewed and may be re‐organized for online delivery, but are not copy‐edited or typeset. Technical support issues arising from supporting information (other than missing files) should be addressed to the authors.

Supporting Information

Supporting Information

Supporting Information

## References

[1a] P. P. Power , Nature 2010, 463, 171–177; 20075912 10.1038/nature08634

[1b] C. Weetman , Chem. Eur. J. 2021, 27, 1941–1954. 32757381 10.1002/chem.202002939 PMC7894548

[2a] C. Dohmeier , C. Robl , M. Tacke , H. Schnöckel , Angew. Chem. Int. Ed. Engl. 1991, 30, 564–565;

[2b] C. Cui , H. W. Roesky , H. G. Schmidt , M. Noltemeyer , H. Hao , F. Cimpoesu , Angew. Chem. Int. Ed. 2000, 39, 4274–4276; 10.1002/1521-3773(20001201)39:23<4274::AID-ANIE4274>3.0.CO;2-K 29711904

[2c] J. Hicks , P. Vasko , J. M. Goicoechea , S. Aldridge , Angew. Chem. Int. Ed. 2021, 60, 1702–1713; 10.1002/anie.202007530 32567755

[3a] P. Bag , A. Porzelt , P. J. Altmann , S. Inoue , J. Am. Chem. Soc. 2017, 139, 14384–14387; 28898060 10.1021/jacs.7b08890

[3b] C. Weetman , A. Porzelt , P. Bag , F. Hanusch , S. Inoue , Chem. Sci. 2020, 11, 4817–4827. 34122939 10.1039/d0sc01561j PMC8159210

[4a] R. J. Wright , A. D. Phillips , P. P. Power , J. Am. Chem. Soc. 2003, 125, 10784–10785; 12952447 10.1021/ja034478p

[4b] the same dialumene may also be trapped by alkynes. C. Cui , X. Li , C. Wang , J. Zhang , J. Cheng , X. Zhu , Angew. Chem. Int. Ed. 2006, 45, 2245–2247; 10.1002/anie.200504329 16518863

[5] T. Agou , K. Nagata , N. Tokitoh , Angew. Chem. Int. Ed. 2013, 52, 10818–10821; 10.1002/anie.201305228 24926488

[6a] K. Nagata , T. Agou , N. Tokitoh , Angew. Chem. Int. Ed. 2014, 53, 3881–3884; 10.1002/anie.201310559 24616148

[6b] T. Agou , K. Nagata , T. Sasamori , N. Tokitoh , Phosphorus Sulfur Silicon Relat. Elem. 2016, 191, 588–590;

[6c] K. Nagata , T. Murosaki , T. Agou , T. Sasamori , T. Matsuo , N. Tokitoh , Angew. Chem. Int. Ed. 2016, 55, 12877–12880; 10.1002/anie.201606684 27529165

[7] C. Weetman , P. Bag , T. Szilvási , C. Jandl , S. Inoue , Angew. Chem. Int. Ed. 2019, 58, 10961–10965; 10.1002/anie.201905045 31112624

[8a] J. Moilanen , P. P. Power , H. M. Tuononen , Inorg. Chem. 2010, 49, 10992–11000; 21049909 10.1021/ic101487g

[8b] for a review of related systems see E. Rivard , P. P. Power , Inorg. Chem. 2007, 46, 10047–10064. 17975890 10.1021/ic700813h

[9] T. Agou , K. Nagata , T. Sasamori , N. Tokitoh , Chem. Asian J. 2014, 9, 3099–3101. 25154494 10.1002/asia.201402798

[10] J. D. Queen , A. Lehmann , J. C. Fettinger , H. M. Tuononen , P. P. Power , J. Am. Chem. Soc. 2020, 142, 20554–20559. 33226797 10.1021/jacs.0c10222

[11] R. L. Falconer , G. S. Nichol , I. V. Smolyar , S. L. Cockroft , M. J. Cowley , Angew. Chem. Int. Ed. 2021, 60, 2047–2052; 10.1002/anie.202011418 PMC7894477 33022874

[12] W. Uhl , Z. Naturforsch. 1988, 43b, 1113–1118.

[13] H. Grützmacher , T. F. Fässler , Chem. Eur. J. 2000, 6, 2317–2325. 10939733 10.1002/1521-3765(20000703)6:13<2317::aid-chem2317>3.0.co;2-x

[14] By calculating *δ*(Al,Al)_ **1** _/*δ*(Al,Al)_ **VI** _ we can estimate the Al−Al bond order in **1** as 1.24. For details see: D. B. Chesnut , Chem. Phys. 2006, 321, 269–276.

[15] C. Gatti , Z. Kristallogr. 2005, 220, 399–457.

[16] For calculations on (NHC)_2_Al_2_X_2_ systems see N. Holzmann , A. Stasch , C. Jones , G. Frenking , Chem. Eur. J. 2011, 17, 13517–13525. 22038936 10.1002/chem.201101915

[17] For a summary see

[17a] M. Kira , T. Iwamoto , Adv. Organomet. Chem. 2006, 54, 73–148. Initial reports:

[17b] E. A. Carter , W. A. Goddard , J. Phys. Chem. 1986, 90, 998–1001;

[17c] J. P. Malrieu , G. Trinquier , J. Am. Chem. Soc. 1989, 111, 5916–5921;

[17d] M. Karni , Y. Apeloig , J. Am. Chem. Soc. 1990, 112, 8589–8590.

[18] *Z* isomers of **1** are ruled out on the basis of their higher energy, see SI.

[19] R. L. Falconer , G. S. Nichol , M. J. Cowley , Inorg. Chem. 2019, 58, 11439–11448. 31411460 10.1021/acs.inorgchem.9b01061 PMC6727621

[20a] H. Zhu , R. B. Oswald , H. Fan , H. W. Roesky , Q. Ma , Z. Yang , H. Schmidt , M. Noltemeyer , K. Starke , N. S. Hosmane , J. Am. Chem. Soc. 2006, 128, 5100–5108; 16608344 10.1021/ja057731p

[20b] C. Cui , S. Koepke , R. Herbst-Irmer , H. W. Roesky , M. Noltemeyer , H.-G. Schmidt , B. Wrackmeyer , J. Am. Chem. Soc. 2001, 123, 9091–9098; 11552816 10.1021/ja003185i

[20c] **6** is also related to the 2-π aromatic aluminacyclopropenes discussed in a recent review, see: K. Ota , R. Kinjo , Chem. Rev. 2021, 121, 10594–10673. 10.1039/d0cs01354d 34369490

[21] Deposition Numbers 2100531 (for **1**), 2100549 (for **4**), 2100550 (for **5**), and 2100551 (for **6**) contain the supplementary crystallographic data for this paper. These data are provided free of charge by the joint Cambridge Crystallographic Data Centre and Fachinformationszentrum Karlsruhe Access Structures service www.ccdc.cam.ac.uk/structures.

