# Supplementary material for: Reversible Dissociation of a Dialumene
Source: Angew Chem Int Ed Engl. 2021 Oct 7;60(46):24702–8. doi: 10.1002/anie.202111385 (PMC8596890; doi:10.1002/anie.202111385)
Supplement: Supplementary file 2 — Supporting Information [file ANIE-60-24702-s003.pdf]

Angewandte Chemie
Eine Zeitschrift der Gesellschaft Deutscher Chemiker

Supporting Information

Reversible Dissociation of a Dialumene**

Rosalyn L. Falconer, Keelan M. Byrne, Gary S. Nichol, Tobias Krämer, and Michael J. Cowley**

anie_202111385_sm_miscellaneous_information.pdf
anie_202111385_sm_cif.zip
anie_202111385_sm_miscellaneous_information.xyz

Synthetic Procedures 4
General Considerations 4
Precursor Synthesis 5
Dihydrodialane VI 5
Diiododialane 2 5
Synthesis of Dialumene 1 6
Other attempted reduction conditions to form 1 7
Solubility and Stability of 1 7
Synthesis of Dialuminacyclobutane 4 8
Synthesis of Dialuminacyclobutene 5 10
Synthesis of aluminacyclopropene 6 12
Stereochemistry of Al(I) and Al(II) compounds 13
Diastereomers of dialumene 1 13
Diastereomers of dialuminacyclobutane 4 and dialuminacyclobutene 5 14
UV-Vis spectroscopy of dialumene 1 16
Low temperature NMR spectroscopy for dialumene 1 17
Density Functional Theory (DFT) calculations 20
Computational Methods 20
Calculated structural parameters of dialumenes I, II, 1, and models $M^1 – M^8$. 21
Comparison of experimental and calculated geometries of I, II and 1 21
Model dialumenes $M^1 – M^8$ 24
Calculated properties of dialumenes I, II, 1, and $M^1 – M^8$. 26
Bond dissociation energies and HOMO/LUMO gaps 26
Properties of aluminyl fragments/monomers 28
Energies and interconversion of diastereomers 1A-1C 30
Calculated ^{31}P NMR chemical shifts of dialumene 1 and dihydrodialane species 32
Results from Time-dependent DFT calculations (TD-DFT) 33
Frontier molecular orbitals of 1 34
Natural Bond Orbital Analyses 35
ELF Analysis 42
QTAIM Analysis 44
X-Ray Crystallography 50
Dialumene 1 50
Dialuminacyclobutane 4 52
Dialuminacyclobutene 5 54
Aluminacyclopropene 6 56
NMR spectra of reported compounds 58

Dialumene 1 58
Compound 4 61
Compound 5 64
Compound 6 67
References 69

Synthetic Procedures

General Considerations

All manipulations were carried out under an argon atmosphere using standard Schlenk or glovebox techniques unless stated. Reactions were carried out in glass Schlenk tubes. Due to the high sensitivity of the compounds synthesised, it was necessary to silanize the glassware prior to use. Glassware was washed with a 5% solution of dichlorodimethylsilane in toluene then allowed to dry. The glassware was then washed with methanol and dried in an oven at 110 °C for at least 16 hours prior to use.

Solvents were obtained from an inert solvent purification system and stored over 4 Å molecular sieves. C_6D_6, d_8-toluene and d_8-THF were dried over a potassium mirror then vacuum distilled and stored over 4 Å molecular sieves. Ambient temperature NMR spectra were recorded on Bruker PRO 500 MHz, AVA 400, 500 or 600MHz spectrometers. ^{1}H and ^{13}C spectra were referenced to residual solvent signals. ^{31}P NMR spectra were referenced to an external standard of 85% H_3PO_4 in H_2O. Mass spectra were acquired using Thermo Mat 900 XP hi resolution double focussing sector mass spectrometer from solid samples. Elemental Analysis was performed by Elemental Microanalysis Ltd. UV-vis spectra were recorded on a Varian Cary 50 Scan UV-vis spectrometer and Shimadzu UV-1800 Spectrometer with VICI-DBS Single Cell Peltier accessory for variable temperature spectra.

Aluminium(II) hydride dimer **VI** and aluminium(II) iodide dimer **2** were synthesised according to a modified literature procedure.[1] Na/K was synthesised by mixing freshly cut Na and K (0.5 g each) until a uniform liquid was achieved. All other reagents were purchased from commercial suppliers and used without further purification.

Key for NMR spectroscopic assignment of ligand resonances:

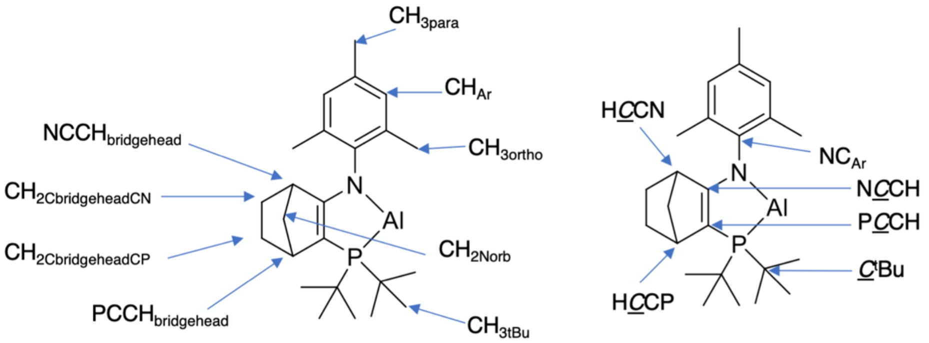

Precursor Synthesis

Dihydrodialane VI

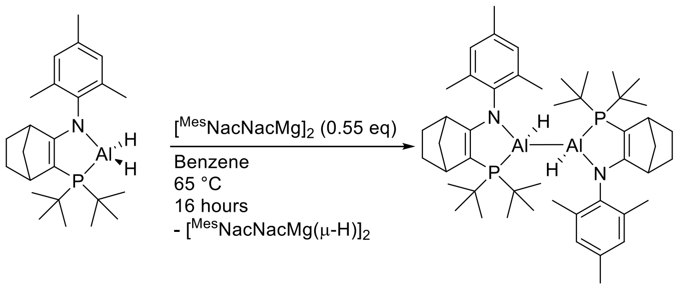

VI

This compound was synthesised according to a modified literature procedure.[1] A stirred solution of NMes/P^{t}Bu$_2$ substituted aluminium dihydride (1.00 g, 2.5 mmol) and [^{Mes}NacNacMg]$_2$ (0.99g, 1.4 mmol, 0.55 eq) in benzene (125 mL) was heated to 65 °C for 16 hours. The resulting solution was cooled to 50 °C, filtered to a flask at room temperature, concentrated to 100 mL and left to crystallise at room temperature. The mixture was filtered, and the crystals washed with benzene and dried to afford the product as a white crystalline solid with identical characterisation to that previously reported. The most common contaminant was the magnesium by-product [^{Mes}NacNacMg(μ-H)]$_2$, which crystallises as large yellow block crystals. The product **VI** must be completely clean of this contaminant for further reactivity to Diiododialane **2**. Typical yields of 400-500 mg (40-50%).

Diiododialane **2**

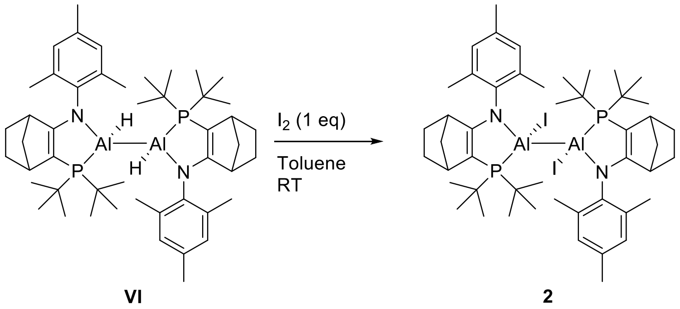

VI **2**

This compound was synthesised according to a modified literature procedure. The preparation was conducted in parallel in two 250 mL round bottomed Schlenk flasks which were then combined during work-up.

Two silanized 250 mL Schlenk flasks were each charged with dihydrodialane **VI** (280.0 mg, 0.351 mmol) and toluene (130 mL). The mixtures were stirred vigorously and heated with a heat gun until all solid had dissolved. The solutions were stirred at room temperature for 30 minutes and then stirred vigorously whilst a solution of I_2 in toluene was added dropwise (4.4 mL of 20 mg/mL solution, 0.351 mmol, 1 eq), accompanied by an immediate colour change to yellow. The solutions were stirred for 30 minutes and then concentrated to 30 mL each. The resultant yellow solutions were filtered into a 100 mL Schlenk flask and the volatiles removed *in vacuo* to afford a pale yellow solid (typical yields of 550-600 mg, 75-80%). Pure material can be afforded by crystallising **2** from toluene at 4 °C, however the initial solid was of sufficient purity for use in the synthesis of dialumene **1**.

Synthesis of Dialumene 1

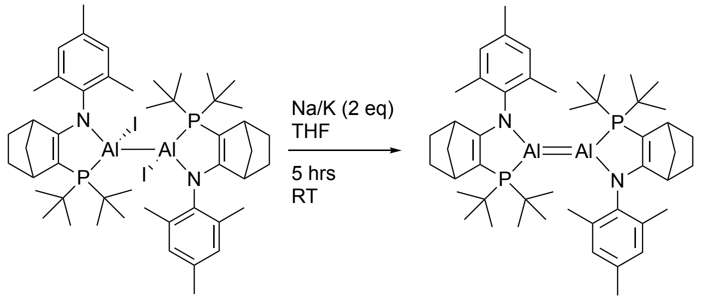

2 **1** (31%)

This synthesis was carried out in two portions, which were then combined for work-up. When reactions were attempted on a larger scale, initiation of the reduction was slow and increased decomposition of the product was observed.

In an Ar filled glovebox, two silanized 100 mL Schlenk flasks were each charged with Na/K (15.9 mg, 0.547 mmol, 2 eq). A solution of Al(II) iodide dimer **2** (287.2 mg, 0.274 mmol, 1 eq.) in THF (50 mL) was added to each flask quickly and the mixtures were stirred vigorously for 5 hours. After approximately 1 hour, the solutions turned intense purple (the exact initiation period depends on purity of starting materials and stirring speed). After 5 hours, the $^{31}P\{^{1}H\}$ NMR spectrum shows the solution contains approximately 40% product by integration. Although starting material is still present in the reaction mixture, higher reaction times result in the formation of higher amounts of by-products and no increase in yield of **1**.

The volatiles were removed from both flasks, then the product was extracted into toluene (total 130 mL) and filtered into a silanized 250 mL flask. The filtrate was concentrated to 40 mL and cooled to -30 °C overnight. The solid was obtained was separated by filtration and the product was afforded as a purple crystalline solid (133 mg, 0.167 mmol, 31%). **1** was stored in the glovebox freezer at -30 °C. Single crystals suitable for X-ray diffraction were grown from a saturated toluene solution at -30 °C.

The product was exhibits two sets of resonances by NMR spectroscopy: **X** (major, 54%) and **Y** (minor, 46%). The two sets correspond to time-averaged signals for diastereomers [**1A**+**1B**], and those for **1C**. We have not been able to assign specific diastereomers. Some resonances are coincident (labelled **X** + **Y**), whilst others are unique (labelled either **X** or **Y**). Due to low solubility of dialumene **1** (<5 mg/mL in C_6D_6), characterisation by ^{13}C was challenging and some peaks were only located using 2D spectra.

^{1}H (300 K, C_6D_6, 500 MHz): δ 6.80 (s, 8H, **X** + **Y**, CH_{meta}), 3.09 (br s, 2H, **Y** $NCCH_{bridgehead}$), 3.06 (br s, 2H, **X** $NCCH_{bridgehead}$), 2.56 (s, 3H, **X** CH_{3ortho}), 2.52 (s, 3H, **Y** CH_{3ortho}), 2.50 (s, 3H, **Y** CH_{3ortho}), 2.44 (s, 3H, **X** CH_{3ortho}), 2.43 (br s, 4H, **X** + **Y**, $PCCH_{bridgehead}$), 2.22 (s, 3H, **X** CH_{3para}), 2.22 (s, 3H, **Y** CH_{3para}), 1.71 (m, 4H, **X** + **Y**, ½ $CH_{2CbridgeheadCN}$), 1.70 (m, 2H, **X** ½ CH_{2Norb}), 1.66 (m, 2H, **Y** ½ CH_{2Norb}), 1.58 (m, 4H, **X** + **Y**, ½ $CH_{2CbridgeheadCN}$), 1.43 (m, 4H, **X** + **Y**, ½ $CH_{2CbridgeheadCP}$), 1.34 (m, 4H, **X** + **Y**, ½ $CH_{2CbridgeheadCP}$), 1.29 (d, $^{3}J_{HP}$ = 13.6 Hz, 9H, **X** CH_{3tBu}), 1.28 (d, $^{3}J_{HP}$ = 13.5 Hz, 9H, **Y** CH_{3tBu}), 1.24 (d, $^{3}J_{HP}$ = 13.6 Hz, 18H, **X** + **Y**, CH_{3tBu}), 1.15 (m, 4H, **X** + **Y**, ½ CH_{2Norb}).

$^{13}C\{^{1}H\}$ (300 K, C_6D_6, 125.8 MHz): δ 180.1 (N$\underline{C}$CH), 143.8 (NC_{Ar}), 136.9 (s, C_{ortho}), 136.7 (s, C_{ortho}), 135.7 (s, C_{ortho}), 135.5 (s, C_{ortho}), 133.0 (s, C_{para}), 133.0 (s, C_{para}), 129.1 (s, CH_{meta}), 129.0 (s, CH_{meta}), 84.4 (P$\underline{C}$CH), 49.3 (d, J_{CP} = 3.3 Hz, CH_{2Norb}), 49.2 (d, J_{CP} = 2.9 Hz, CH_{2Norb}), 45.0 (d, J_{CP} = 4.2 Hz, H$\underline{C}$CN), 44.9 (d, J_{CP} = 4.2 Hz, H$\underline{C}$CN), 43.8 (m, H$\underline{C}$CP), 34.0

(d, J_{CP} = 14.2 Hz, $\underline{C}^tBu$), 31.0 (d, J_{CP} = 6.4 Hz, CH_{3tBu}), 30.9 (d, J_{CP} = 4.8 Hz, CH_{3tBu}), 30.5 (m, CH_{3tBu}), 30.1 (d, J_{CP} = 19.0 Hz, $CH_{2CbridgeheadCN}$), 25.4 (d, J_{CP} = 12.0 Hz, $CH_{2CbridgeheadCP}$), 21.0 (s, CH_{3para}), 21.0 (s, CH_{3para}), 20.3 (s, CH_{3ortho}), 20.2 (s, CH_{3ortho}), 19.6 (s, CH_{3ortho}), 19.5 (s, CH_{3ortho}).

$^{31}P\{^1H\}$ (300 K, C_6D_6, 162.0 MHz): δ 21.3 (br s, $\Delta\nu_{1/2}$ = 134.4 Hz).

Elemental Analysis: Found (%): C, 73.65; H 9.23; N, 3.21. Calc. for $C_{51.5}H_{78}Al_2N_2P_2$ (**1** with 0.5 eq toluene): C, 73.54; H, 9.35; N, 3.33.

High Resolution Mass Spec (APPI): Dimer m/z = 794.49152 $[C_{48}H_{74}Al_2N_2P_2]^+$ (theoretical = 794.49526). Monomer m/z = 397.24576 $[C_{24}H_{37}AlNP]^+$ (theoretical = 397.24736).

UV-vis (hexane solution): 567.0 nm (Ɛ = 10362 L mol^{-1} cm^{-1})

Other attempted reduction conditions to form 1

A number of other conditions were attempted for the reduction of **2** to dialumene **1**. From our observations, THF solvent is important (reactions in toluene or benzene were slow or did not occur). Similarly, closely-stoichiometric quantities of reductant are essential to avoid decomposition by removal of the ligand. Short reaction times are also neccessary to limit decomposition of **1** in solution. Thus, reductions of **2** with KC_8 were too slow. Reactions with dropwise addition of 2 equivalents of K(naphthalenide) or Li(naphthalenide) at room temperature afforded instant conversion to **1** in a similar conversion to Na/K, but had the added complication of naphthalene contaminant in the resulting product. We therefore chose to optimise the reaction with Na/K.

Extraction of the product following reduction was attempted with pentane, but the higher solubility of **1** in toluene meant work-up in toluene was easier and faster (affording lower decomposition of **1** and higher yields).

Solubility and Stability of 1

Dialumene **1** is poorly soluble in a range of solvents. Solubility is highest in THF, benzene or toluene, but is limited to < 6 mg/mL. The solubility of **1** in hexane is lower.

1 is somewhat unstable in solution, depending on the solvent. After 6 hours in THF, benzene, or hexane, solution we found that 3%, 10%, or 20 % (respectively) of **1** had decomposed. The principal decomposition product is the dihydrodialane **VI**.

In the solid state, dialumene **1** decomposes slowly (days) at room temperature, and is thus best stored at –30 °C.

Synthesis of Dialuminacyclobutane 4

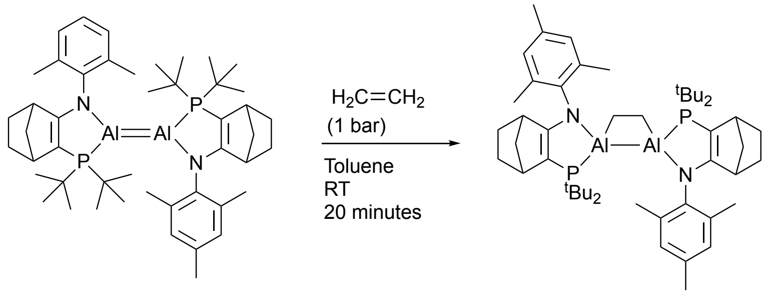

1 **4**

A J Young's ampoule was charged with dialumene **1** (100.0 mg, 0.126 mmol) and toluene (25 mL). The solution was degassed three times by freeze/pump/thaw and the flask was then refilled with ethene at a pressure of 1 atm. The flask was shaken, and then the solution was vigorously stirred. After 20 minutes, a colour change from dark purple to pale yellow was observed. The solution was stirred for a further 30 minutes at room temperature. The volatiles were removed *in vacuo* and the product extracted into pentane, filtered, concentrated, and cooled to -30 °C. The resultant solid was redissolved, filtered, and recrystallised to afford **4** as colourless crystals (20.0 mg, 0.0243 mmol, 19%). The high solubility of the product precluded higher yields. Crystals suitable for X-ray diffraction were grown from a saturated pentane solution at room temperature.

Reactions carried out on an NMR scale (3 mg of dialumene **1** in 0.5 mL C_6D_6) in shaken tubes were complete in 5 minutes.

4 exists as three diastereomers (X, Y and Z). Overlapping resonances precluded the determination of the ratio of these isomers and their individual characterisation.

^{1}H (300 K, C_6D_6, 500 MHz): δ 6.86 (m, 4H, CH_{meta}), 3.03 (br s, 2H, $NCCH_{bridgehead}$), 2.95 (br s, 2H, $NCCH_{bridgehead}$), 2.45 (br s, 2H, $PCCH_{bridgehead}$), 2.43 (s, 3H, CH_{3ortho}), 2.42 (s, 3H, CH_{3ortho}), 2.42 (s, 3H, CH_{3ortho}), 2.39 (br s, 2H, $PCCH_{bridgehead}$), 2.36 (s, 3H, CH_{3ortho}), 2.35 (s, 3H, CH_{3ortho}), 2.34 (s, 3H, CH_{3ortho}), 2.27 (s, 3H, CH_{3para}), 2.26 (s, 3H, CH_{3para}), 1.71 (m, $CH_{2CbridgeheadCN}$), 1.66 (m, 2H, ½ CH_{2Norb}), 1.65 (m, $CH_{2CbridgeheadCN}$), 1.56 (m, $CH_{2CbridgeheadCN}$), 1.42 (m, $CH_{2CbridgeheadCP}$), 1.37 (m, $AlCH_2$), 1.32 (m, $AlCH_2$), 1.29 (m, $AlCH_2$), 1.27 (d, $^3J_{HP}$ = 13.3 Hz, CH_{3tBu}), 1.25 (d, $^3J_{HP}$ = 14.0 Hz, CH_{3tBu}), 1.22 (m, $CH_{2CbridgeheadCP}$), 1.22 (d, $^3J_{HP}$ = 13.8 Hz, CH_{3tBu}), 1.12 (m, 2H, ½ CH_{2Norb}), 1.07 (d, $^3J_{HP}$ = 13.9 Hz, CH_{3tBu}), 1.06 (d, $^3J_{HP}$ = 13.5 Hz, CH_{3tBu}), 1.00 (d, $^3J_{HP}$ = 12.6 Hz, CH_{3tBu}), 0.97 (d, $^3J_{HP}$ = 13.9 Hz, CH_{3tBu}), 0.50 (m, $AlCH_2$), 0.39 (m, $AlCH_2$).

^{113}C{^{1}H} (300 K, C_6D_6, 125.8 MHz): δ 181.5 (m, NC_{Ar}), 144.1 (d, J_{CP} = 3.9 Hz, N*C*CH), 144.0 (d, J_{CP} = 3.8 Hz, N*C*CH), 143.8 (apparent t, J_{CP} = 1.8 Hz, N*C*CH), 137.0 (s, CH_{3ortho}), 136.9 (s, CH_{3ortho}), 136.5 (s, CH_{3ortho}), 136.3 (s, CH_{3ortho}), 136.0 (s, CH_{3ortho}), 135.9 (s, CH_{3ortho}), 135.8 (s, CH_{3ortho}), 135.8 (s, CH_{3ortho}), 133.5 (s, CH_{3para}), 133.3 (s, CH_{3para}), 133.2 (s, CH_{3para}), 129.7 (s, CH_{meta}), 129.7 (s, CH_{meta}), 129.7 (s, CH_{meta}), 129.4 (s, CH_{meta}), 129.3 (s, CH_{meta}), 129.2 (s, CH_{meta}), 128.9 (s, CH_{meta}), 128.9 (s, CH_{meta}), 82.5 (m, P*C*CH), 82.2 (m, P*C*CH), 49.2 (s, CH_{2Norb}), 49.2 (s, CH_{2Norb}), 48.7 (d, J_{CP} = 4.0 Hz, CH_{2Norb}), 44.5 (d, J_{CP} = 3.3 Hz, $CH_{bridgeheadCN}$), 44.4 (m, $CH_{bridgeheadCN}$), 43.8 (m, $CH_{bridgeheadCP}$), 43.7 (m, $CH_{bridgeheadCP}$), 35.9 (d, J_{CP} = 14.8 Hz, *C*^{t}Bu), 35.6 (d, J_{CP} = 17.6 Hz, *C*^{t}Bu), 35.5 (d, J_{CP} = 18.2 Hz, *C*^{t}Bu), 33.4 (d, J_{CP} = 18.6 Hz, *C*^{t}Bu), 33.3 (d, J_{CP} = 18.8 Hz, *C*^{t}Bu), 33.1 (d, J_{CP} = 21.0 Hz, *C*^{t}Bu), 30.8 (d, J_{CP} = 4.6 Hz, CH_{3tBu}), 30.8 (d, J_{CP} = 4.5 Hz, CH_{3tBu}), 30.7 (d, J_{CP} = 4.1 Hz, CH_{3tBu}), 30.4 (d, J_{CP} =

4.3 Hz, CH_{3tBu}), 30.3 (s, $CH_{2CbridgeheadCN}$), 30.1 (s, $CH_{2CbridgeheadCN}$), 30.0 (d, J_{CP} = 5.0 Hz, CH_{3tBu}), 29.9 (s, $CH_{2bridgeheadCN}$), 29.8 (d, J_{CP} = 6.0 Hz, CH_{3tBu}), 29.8 (d, J_{CP} = 4.8 Hz, CH_{3tBu}), 29.5 (d, J_{CP} = 4.3 Hz, CH_{3tBu}), 25.5 (s, $CH_{2CbridgeheadCP}$), 21.1 (s, CH_{3para}), 21.0 (s, CH_{3para}), 20.5 (s, CH_{3ortho}), 20.4 (s, CH_{3ortho}), 20.0 (s, CH_{3ortho}), 20.0 (s, CH_{3ortho}), 19.4 (s, CH_{3ortho}), 19.3 (s, CH_{3ortho}), 19.3 (s, CH_{3ortho}), 10.3 (s, Al$\underline{C}H_2$), 10.1 (s, Al$\underline{C}H_2$), 9.6 (s, Al$\underline{C}H_2$).

$^{31}P\{^1H\}$ (300 K, C_6D_6, 162.0 MHz): δ 11.6 (d, J_{PP} = 12.2 Hz, **X**), 11.5 (s, **Y**), 11.5 (s, **Z**), 11.4 (d, J_{PP} = 12.2 Hz, **X**).

Elemental Analysis: Despite repeated attempts compound **4** proved unsuitable for combustion analysis. Found (%): C, 73.60; H, 9.58; N, 3.25. Calc. for $C_{50}H_{78}Al_2N_2P_2$: C, 72.96; H, 9.55; N, 3.40.

High Resolution Mass Spec (APPI): m/z = 822.52583 $[C_{50}H_{78}Al_2N_2P_2]^+$ (theoretical = 822.52656).

Synthesis of Dialuminacyclobutene 5

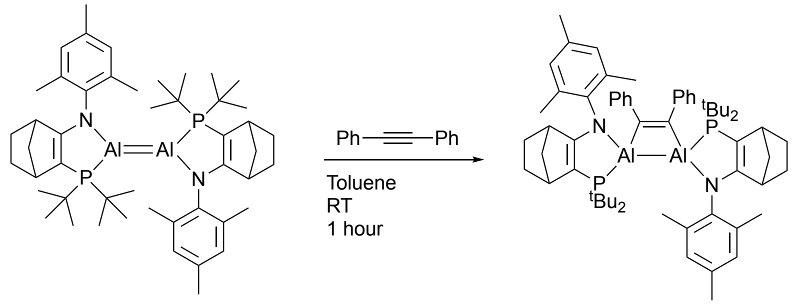

1 **5**

To a stirred solution of dialumene **1** (96.8 mg, 0.122 mmol, 1eq) in toluene (30 mL), a solution of diphenylacetylene (21.7 mg, 0.122 mmol, 1 eq) in 5 mL toluene was added. The resultant solution was stirred for 1 hour to afford a bright yellow solution. The volatiles were removed *in vacuo* and the product extracted into pentane (30 mL), concentrated, and cooled to -30 °C overnight. The first crop of solid was contaminated with the products of decomposition, so was recrystallised in pentane at -30 °C to afford **5** as a yellow crystalline solid (67.3 mg, 0.069 mmol, 57%).

5 exists as three diastereomers (41% **X**, 12% **Y** and 47% **Z**). Due to overlapping resonances, it was not possible to distinguish/assign individual diastereomers in the ^{1}H and ^{13}C spectra.

^{1}H (300 K, C_6D_6, 500 MHz): δ 7.19 (quintet, 4H, H_{Ar}), 7.01-6.82 (m, 10H, H_{Ar}), 2.93 (br s, 1H, $NCCH_{bridgehead}$), 2.86 (br s, 1H, $NCCH_{bridgehead}$), 2.55 (s, CH_{3Ar}), 2.47 (s, CH_{3Ar}), 2.43 (s, CH_{3Ar}), 2.33 (br s, 1H, $PCCH_{bridgehead}$), 2.31 (s, CH_{3Ar}), 2.30 (s, CH_{3Ar}), 2.26 (s, CH_{3Ar}), 2.22 (s, CH_{3Ar}), 1.63 (m, 2H, ½ $CH_{2CbridgeheadCP}$), 1.61 (m, 2H, ½ CH_{2Norb}), 1.57 (m, 2H, ½ $CH_{2CbridgeheadCP}$), 1.55 (m, 2H, ½ $CH_{2CbridgeheadCP}$), 1.45 (m, 2H, ½ CH_{2Norb}), 1.43 (m, 2H, ½ $CH_{2CbridgeheadCP}$), 1.33 (m, 2H, ½ $CH_{2CbridgeheadCN}$), 1.16 (m, 2H, ½ $CH_{2CbridgeheadCN}$), 1.13 (d, 9H, J_{CP} = 14.3 Hz, CH_{3tBu}), 1.12 (d, 9H, J_{CP} = 13.7 Hz, CH_{3tBu}), 1.10 (d, 9H, J_{CP} = 13.6 Hz, CH_{3tBu}), 1.06 (d, 9H, J_{CP} = 13.6 Hz, CH_{3tBu}), 1.05 (m, 2H, ½ CH_{2Norb}), 1.04 (d, 9H, J_{CP} = 13.6 Hz, CH_{3tBu}), 1.04 (d, 9H, J_{CP} = 13.8 Hz, CH_{3tBu}), 1.02 (d, 9H, J_{CP} = 13.4 Hz, CH_{3tBu}), 1.02 (m, 2H, ½ CH_{2Norb}), 1.01 (d, 9H, J_{CP} = 13.7 Hz, CH_{3tBu}).

^{13}C{^{1}H} (300 K, C_6D_6, 125.8 MHz): δ 185.1 (Al$\underline{C}$Ph), 184.8 (Al$\underline{C}$Ph), 182.1 (d, J_{CP} = 21.7 Hz, N$\underline{C}$CH), 181.6 (d, J_{CP} = 21.4 Hz, N$\underline{C}$CH), 181.5 (d, J_{CP} = 21.7 Hz, N$\underline{C}$CH), 150.5 (s, AlC$\underline{C}_{Ar}$), 150.4 (s, AlC$\underline{C}_{Ar}$), 149.9 (s, AlC$\underline{C}_{Ar}$), 144.8 (m, NC_{Ar}), 144.5 (d, J_{CP} = 4.5 Hz, NC_{Ar}), 144.3 (d, J_{CP} = 4.5 Hz, NC_{Ar}), 137.2 (s, $\underline{C}_{Ar}CH_3$), 137.2 (s, $\underline{C}_{Ar}CH_3$), 136.9 (s, $\underline{C}_{Ar}CH_3$), 136.9 (s, $\underline{C}_{Ar}CH_3$), 136.4 (s, $\underline{C}_{Ar}CH_3$), 136.3 (s, $\underline{C}_{Ar}CH_3$), 136.0 (s, $\underline{C}_{Ar}CH_3$), 135.9 (s, $\underline{C}_{Ar}CH_3$), 133.6 (s, $\underline{C}_{Ar}CH_3$), 133.6 (s, $\underline{C}_{Ar}CH_3$), 133.5 (s, $\underline{C}_{Ar}CH_3$), 133.5 (s, $\underline{C}_{Ar}CH_3$),129.9 (s, CH_{Ar}), 129.8 (s, CH_{Ar}), 129.8 (s, CH_{Ar}), 129.7 (s, CH_{Ar}), 129.4 (s, CH_{Ar}), 129.4 (s, CH_{Ar}), 129.1 (s, CH_{Ar}), 128.6 (s, CH_{Ar}), 128.5 (s, CH_{Ar}), 127.5 (s, CH_{Ar}), 127.5 (app t, J_{CP} = 7.7 Hz, CH_{Ar}), 123.3 (s, CH_{Ar}), 123.3 (s, CH_{Ar}), 123.3 (s, CH_{Ar}), 123.2 (s, CH_{Ar}), 82.9 (m, P$\underline{C}$CH), 49.0 (m, CH_{2Norb}), 48.8 (s, CH_{2Norb}), 48.6 (d, J_{CP} = 4.1 Hz, CH_{2Norb}), 44.5 (s, H$\underline{C}$CP), 44.4 (m, H$\underline{C}$CP), 44.2 (m, H$\underline{C}$CP), 34.6 (d, J_{CP} = 16.0 Hz, $\underline{C}^{t}$Bu), 34.6 (d, J_{CP} = 17.1 Hz, $\underline{C}^{t}$Bu), 34.2 (d, J_{CP} = 17.8 Hz, $\underline{C}^{t}$Bu), 33.3 (d, J_{CP} = 19.0 Hz, $\underline{C}^{t}$Bu), 33.1 (d, J_{CP} = 20.7 Hz, $\underline{C}^{t}$Bu), 33.0 (d, J_{CP} = 20.2 Hz, $\underline{C}^{t}$Bu), 31.0 (d, J_{CP} = 5.3 Hz, CH_{3tBu}), 30.4 (d, J_{CP} = 4.2 Hz, CH_{3tBu}), 30.1 (br s, $CH_{2CbridgeheadCP}$), 30.0 (d, J_{CP} = 7.0 Hz, CH_{3tBu}), 29.9 (d, J_{CP} = 6.2 Hz, CH_{3tBu}), 29.8 (d, J_{CP} = 5.7 Hz, CH_{3tBu}), 29.8 (s, $CH_{2CbridgeheadCP}$), 29.7 (s, $CH_{2CbridgeheadCP}$), 29.6 (d, J_{CP} = 3.6 Hz,

CH_{3tBu}), 25.4 (s, $CH_{2bridgeheadCN}$), 21.7 (s, CH_{3Ar}), 21.5 (s, CH_{3Ar}), 21.1 (s, CH_{3Ar}), 21.1 (s, CH_{3Ar}), 21.1 (s, CH_{3Ar}), 21.0 (s, CH_{3Ar}), 21.0 (s, CH_{3Ar}), 20.9 (s, CH_{3Ar}), 20.9 (s, CH_{3Ar}), 20.8 (s, CH_{3Ar}), 20.7 (s, CH_{3Ar}), 20.5 (s, CH_{3Ar}).

$^{31}P\{^1H\}$ (300 K, C_6D_6, 162.0 MHz): δ 11.0 (br s, **X**), 10.7 (s, **Y**), 10.4 (s, **Z**), 10.3 (br s, **X**).

Elemental Analysis: Found (%): C, 76.41; H, 8.76; N, 3.07. Calc. for $C_{62}H_{84}Al_2N_2P_2$: C, 76.51; H, 8.70; N, 2.88.

Synthesis of aluminacyclopropene 6

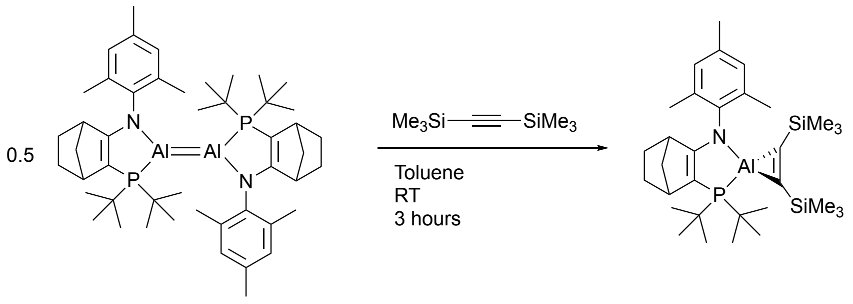

1 **6**

To a stirred solution of dialumene **1** (90.3 mg, 0.114 mmol, 1eq) in toluene (30 mL), a solution of diphenylacetylene (38.7 mg, 0.227 mmol, 2 eq) in 5 mL toluene was added. The resultant solution was stirred for 3 hours to afford a bright yellow solution. The volatiles were stored at -30 °C overnight. The first crop of solid was contaminated with the products of decomposition, so was recrystallised in toluene at -30 °C to afford **6** as a yellow crystalline solid (46.7 mg, 0.082 mmol, 36%). High solubility of the product in toluene precluded a higher yield.

^{1}H (300 K, C_6D_6, 500 MHz): δ 6.78 (s, 1H, CH_{meta}), 6.74 (s, 1H, CH_{meta}), 2.90 (br s, 1H, $NCCH_{bridgehead}$), 2.48 (br s, 1H, $PCCH_{bridgehead}$), 2.34 (s, 3H, CH_{3para}), 2.31 (s, 3H, CH_{3ortho}), 2.06 (s, 3H, CH_{3ortho}), 1.61 (m, 1H, ½ $CH_{2CbridgeheadCN}$), 1.48 (dm, $^2J_{HH}$ = 8.1 Hz, ½ CH_{2Norb}), 1.44 (m, 1H, ½ $CH_{2CbridgeheadCN}$), 1.33 (m, 1H, ½ $CH_{2CbridgeheadCP}$), 1.21 (d, $^3J_{HP}$ = 15.0 Hz, 9H, CH_{3tBu}), 1.19 (d, $^3J_{HP}$ = 15.0 Hz, 9H, CH_{3tBu}), 1.15 (m, 1H, ½ $CH_{2CbridgeheadCP}$), 1.02 (dm, $^2J_{HH}$ = 8.1 Hz, 1H, ½ CH_{2Norb}), 0.42 (s, 9H, $SiCH_3$), 0.33 (s, 9H, $SiCH_3$).

^{13}C{^{1}H} (300 K, C_6D_6, 125.8 MHz): δ 226.6 (s, Al$\underline{C}$Si), 223.7 (s, Al$\underline{C}$Si), 184.5 (d, J_{CP} = 15.8 Hz, N$\underline{C}$CH), 141.0 (d, J_{CP} = 2.7 Hz, NC_{Ar}), 135.3 (s, C_{ortho}), 134.7 (s, C_{ortho}), 134.2 (s, C_{para}), 129.3 (s, CH_{meta}), 129.0 (s, CH_{meta}), 79.2 (d, J_{CP} = 48.1 P$\underline{C}$CH), 48.0 (d, J_{CP} = 4.3 Hz, CH_{2Norb}), 44.1 (d, J_{CP} = 7.8 Hz, H$\underline{C}$CP), 43.9 (d, J_{CP} = 0.9 Hz, H$\underline{C}$CN), 34.3 (d, J_{CP} = 26.5 Hz, $\underline{C}^t$Bu), 33.9 (d, J_{CP} = 24.7 Hz, $\underline{C}^t$Bu), 30.1 (s, $CH_{2CbridgeheadCN}$), 30.0 (d, J_{CP} = 4.7 Hz, CH_{3tBu}), 29.5 (d, J_{CP} = 4.8 Hz, CH_{3tBu}), 25.2 (d, J_{CP} = 1.5 Hz, $CH_{2CbridgeheadCP}$), 20.9 (s, CH_{3para}), 19.4 (s, CH_{3ortho}), 19.3 (s, CH_{3ortho}), 1.4 (s, $SiCH_3$), 1.2 (s, $SiCH_3$).

^{31}P{^{1}H} (300 K, C_6D_6, 162.0 MHz): δ 9.8 (br s, Δν½ = 149.7 Hz).

Elemental Analysis: Found (%): C, 67.68; H, 9.71; N, 2.52. Calc. for $C_{32}H_{55}AlNPSi_2$: C, 67.68; H, 9.76; N, 2.47.

Stereochemistry of Al(I) and Al(II) compounds

Diastereomers of dialumene 1

Dialumene **1** contains 6 stereogenic centres: two in each norbornene unit of the ligand backbone and one at each Al atom due to the *trans*-bending of the Al–Al bond. These stereogenic centres result in 6 distinct possible diastereomers. This is analogous to the N,P-stabilised Al(II) hydride dimers we previously reported.[1] A full stereochemical explanation of the 6 diastereomers was provided in that previous publication.

Diastereomers **A**, **B** and **C** all contain *trans*-P atoms with respect to the Al–Al bond. These diastereomers are found in the X-ray crystal structure. Diastereomers **D**, **E** and **F** all contain *cis*-P atoms with respect to the Al–Al bond. We computed the relative energies of diastereomers **A-F** for dialumene **1** and found **A**, **B** and **C** to be very similar in energy (within error of the computational methods used). Diastereomers **A-C** are more stable than diastereomers **D**, **E** and **F**.

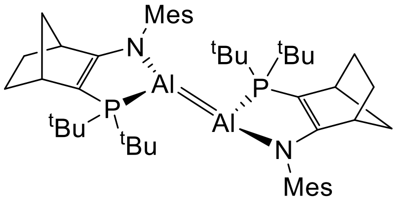

A 1.91 kcal/mol

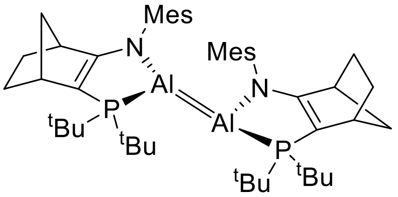

D 5.66 kcal/mol

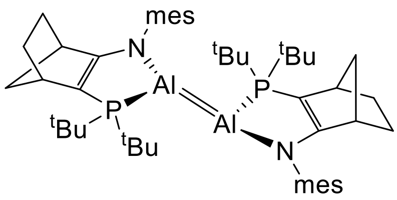

B 0.00 kcal/mol

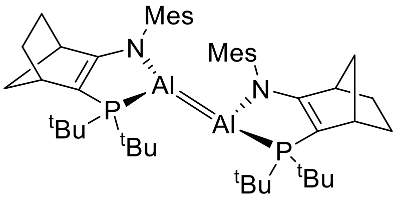

E 5.84 kcal/mol

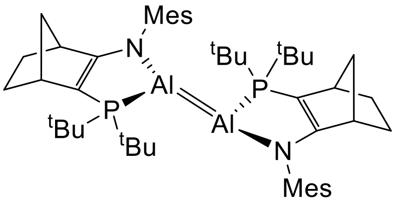

C 1.28 kcal/mol

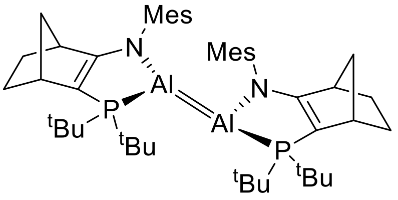

F 4.72 kcal/mol

Figure S1: Relative energies of diastereomers **A-F** of the Al(I) species (Gibbs free energy). See computational methods sections below for further details.

Diastereomers of dialuminacyclobutane 4 and dialuminacyclobutene 5

Reaction of diastereomers **A**, **B** and **C** of dialumene **1** in a [2+2] cycloaddition with ethylene or diphenylacetylene results in the formation of three diastereomeric products. Below is an illustration of the possible products from reaction with ethylene. The analysis applies equally to **5**.

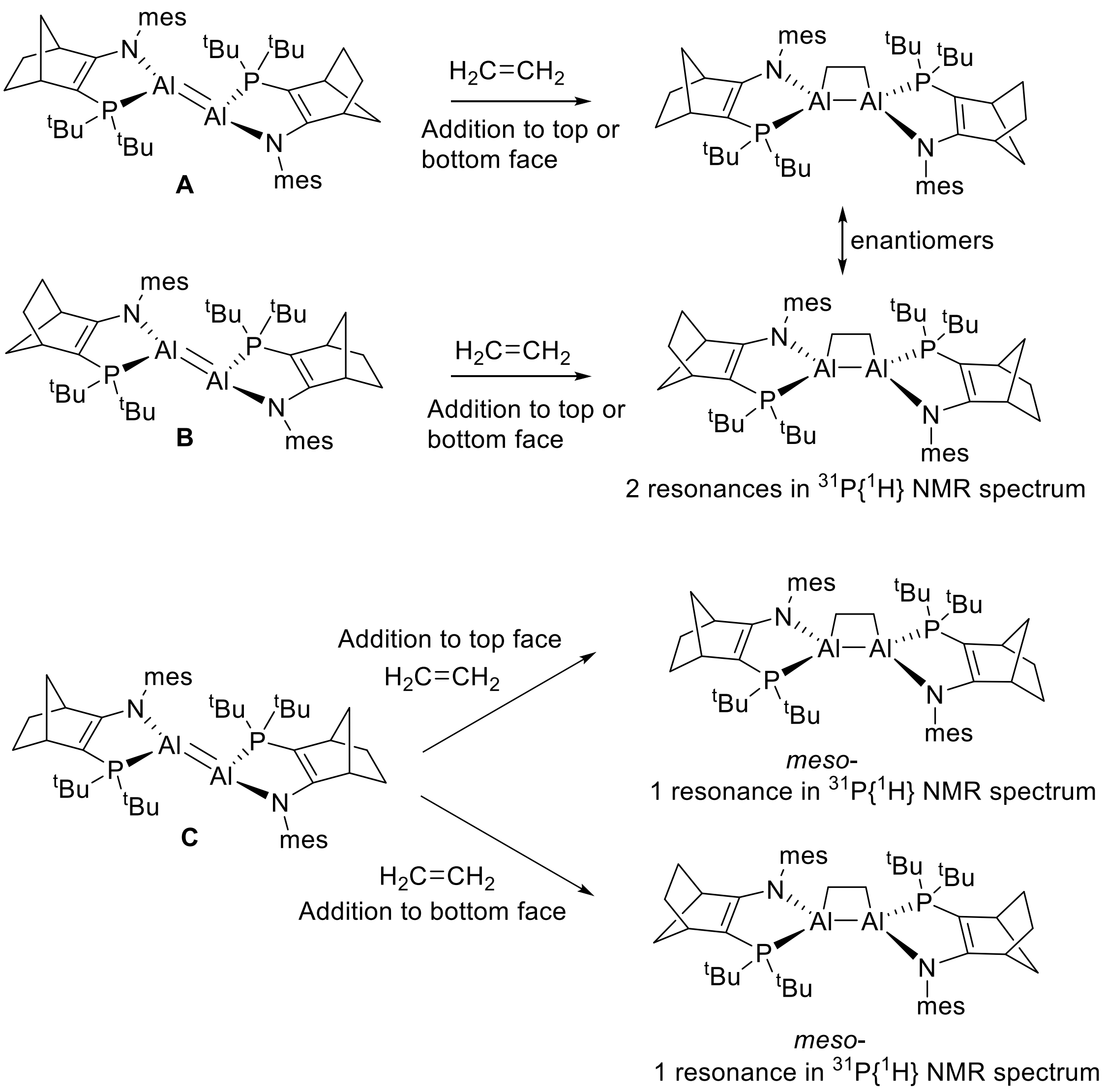

Figure S2: The possible diastereomers of **4** formed from reaction of ethylene with dialumene **1A-C**. The analysis applies equally to **5**, formed from **1** and diphenylacetylene.

Reaction of **A** or **B** with ethylene results in the formation of one diastereomeric product irrespective of addition to the 'top' or 'bottom' face of the Al=Al bond. The products from reaction of diastereomers **A** and **B** are enantiomeric, and thus have identical $^{31}P\{^{1}H\}$ NMR chemical shifts. The products have two ^{31}P resonances because the P atoms are inequivalent (due to the influence of the norbornene ring, which is in opposite orientations at each Al/P centre). In the product from reaction with ethylene (**4**), these are two mutually coupled doublets, but in the product from reaction with diphenylacetylene (**5**), these are two

singlets, likely due to a change in the P–Al–Al–P dihedral angle and therefore lower value for $^{3}J_{P-P}$.

When ethylene adds to the third dialumene diastereomer, **1-C**, addition to the top or bottom face of the Al=Al bond generates two distinct products. This is due to the relative orientation of the norbornene ligand backbone units with respect to the new C-C bridge. Both products are *meso*-compounds and so the P atoms are in equivalent environments, resulting in each compound appearing as a singlet in the $^{31}P\{^{1}H\}$ NMR spectrum of **4**.

UV-Vis spectroscopy of dialumene 1

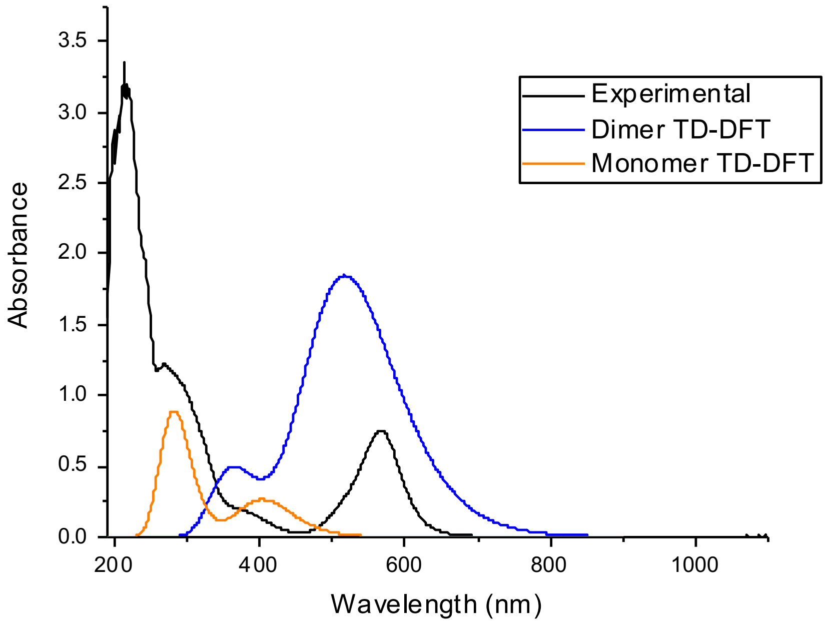

Figure S3: experimental UV-vis of **1** (hexane solution) and those predicted using TD-DFT (SMD-B3LYP-D3/6-311G(2d,2p)). λ_{max} = 567.0 nm (ε = 10362 L mol^{-1} cm^{-1}).

A series of variable temperature UV-vis spectra of **1** in hexane or toluene were recorded from 5 to 65 °C. We observed no change in λ_{max} over this temperature range compared to the spectra at room temperature.

Comparing the experimental UV-Vis spectrum of **1** (λ_{max} = 567.0 nm) to that predicted by TD-DFT reveals good agreement, albeit with some blue-shift of the lowest energy absorption (δ = –47 nm, λ_{max} = 520nm, Figure S4). TD-DFT predicts an absorption for the aluminyl monomer **3** at 410nm. We could observe no such absorption in solutions of **1** over the temperature range 5 to 65 °C, indicating that **3** is not present in substantial (observable) concentration. (the predicted absorption of **3** at 284nm arises from a HOMO to LUMO transition (Al lone pair to Al p orbital) and a HOMO-1 to LUMO+1 transition (associated with the ligand backbone), but experimentally would be obscured by absorptions (visible in the experimental spectrum) from arene π-π* transitions).

Low temperature NMR spectroscopy for dialumene 1

Variable temperature ^{1}H and $^{31}P\{^{1}H\}$ NMR spectroscopy studies were performed on d_8-toluene solutions of dialumene **1** over the temperature range of 188 – 300 K. Due to the low solubility and stability of **1**, decomposition was observed in both sets of spectra over the course of the experiment.

Other than the expected variation of chemical shift with temperature, ^{1}H spectra over the range 188 – 300 K are very similar. Across all temperatures, the ^{1}H NMR spectrum contains two full sets of ligand peaks, indicating two distinct ligand environments.

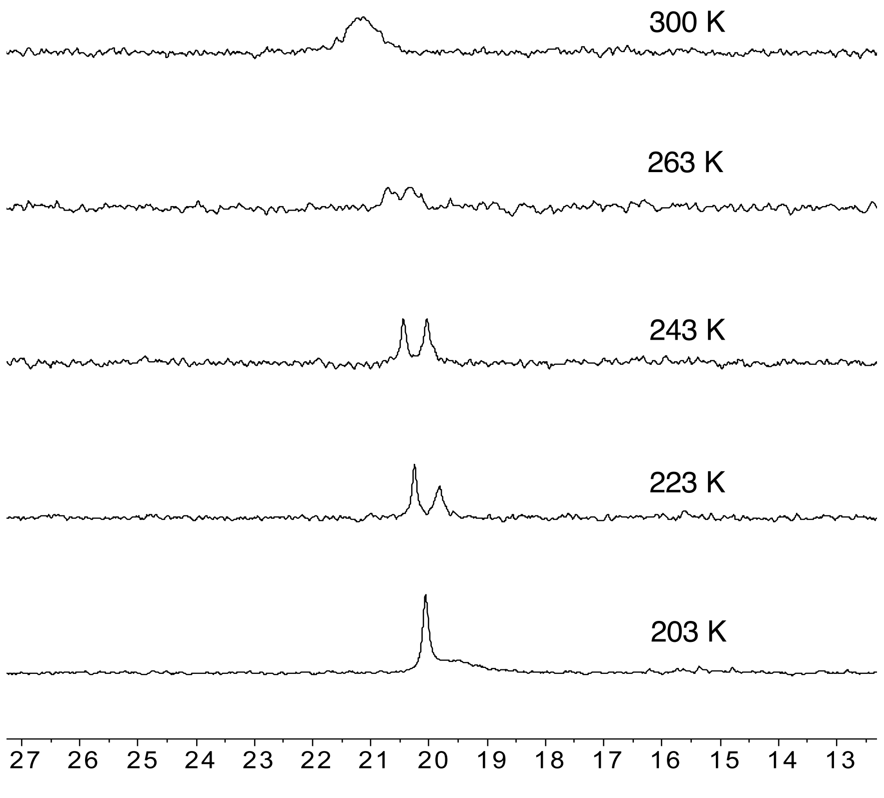

Figure S4: $^{31}P\{^{1}H\}$ NMR spectra for dialumene **1** over temperature range of 203 to 300 K.

In the ^{1}H NMR data, two full sets of ligand resonances are observed. These are assigned to the time averaged environments of isomers **A/B** and **C**. From ^{1}H NMR spectra taken at low temperatures (188-243 K), it was possible to extract thermodynamic data on the interconversion of **A/B** and **C** (Figure S5, Table S1).

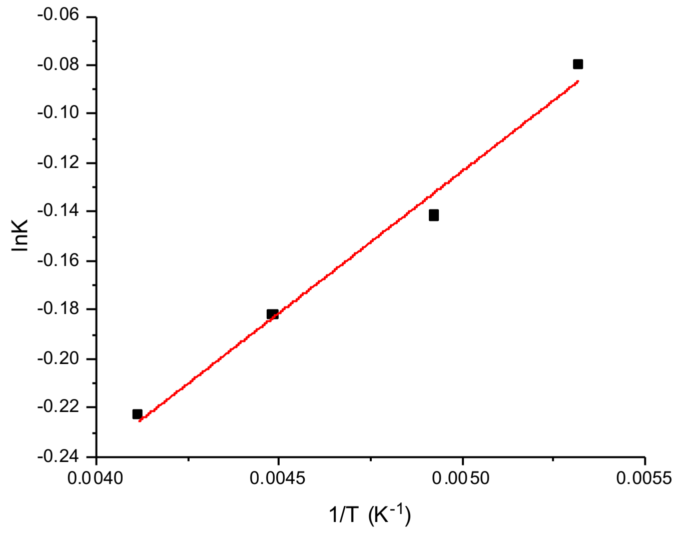

Figure S5: Graph of lnK vs 1/T from concentration data extracted from ^{1}H NMR spectra of **1** at 188-243 K. Values taken from integrating the NCC<u>H</u> resonances for each isomer. Associated parameters given in the table.

Table S1: Thermodynamic parameters extracted from Figure S5

Intercept	Slope	R^2 (COD)	ΔH / kJ mol^{-1}	ΔS (J K^{-1} mol^{-1})	ΔG (kJ mol^{-1})
−0.70 ± 0.04	116 ± 9	0.98721	−0.96 ± 0.08	−5.8 ± 0.4	0.80 ± 0.2

These experimentally determined values for ΔH, ΔS and ΔG indicate that isomers **A/B** and **C** are very close in energy, thereby corroborating DFT calculations.

At 300 K, using ^{1}H EXSY NMR experiments it is possible to observe intermolecular exchange between **1A/B** and **1C.** (Figure S6 and S7).

We could not assign which signals in the ^{1}H NMR spectrum correspond to **1A/B** and which to **1C**, so the two environments are here labelled **X** and **Y**. From the 1H 2D-EXSY experiment, clear exchange cross peaks are present between the resonances for the mesityl-CH_3 and bridgehead NCC<u>H</u> H atoms for the two isomers, indicating exchange between the **1A/B** and **1C**.

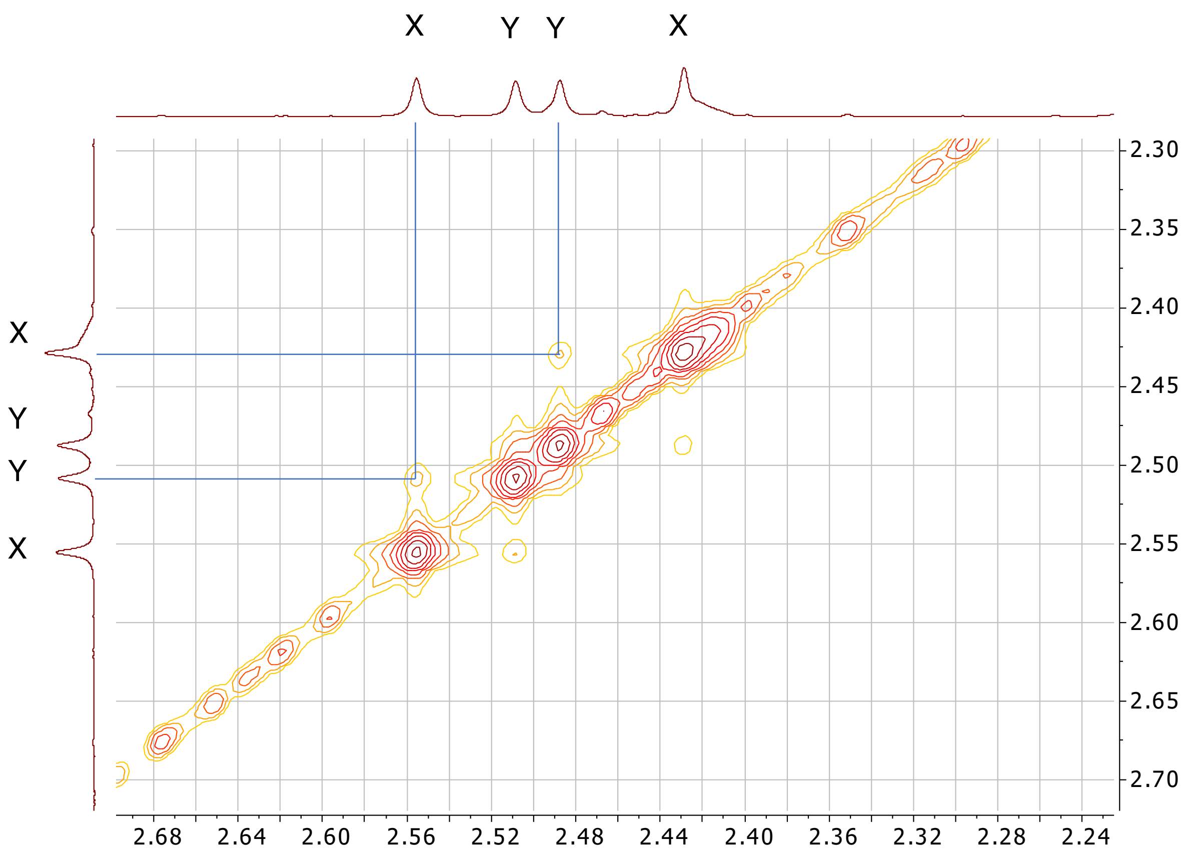

Figure S6: [1]H EXSY experiment on **1** carried out in C_6D_6 at 300 K with a 0.3 s mixing time. Expansion of diagonal and cross peaks for mesityl methyl groups assigned to **1A/B** and **1C**.

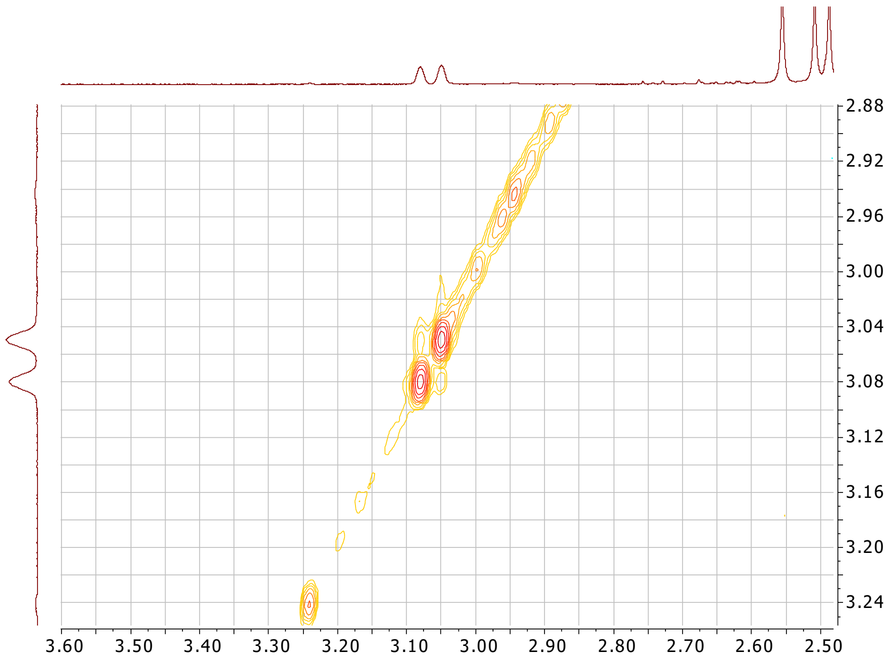

Figure S7: [1]H EXSY experiment on **1** carried out in C_6D_6 at 300 K with a 0.3 s mixing time. Expansion of NCC**<u>H</u>** diagonal and cross peaks for bridgehead NCC**<u>H</u>** H signals observed for **1A/B** and **1C**.

Density Functional Theory (DFT) calculations

Computational Methods

All electronic structure calculations were carried out using the Gaussian 16 (Revision B.01) program.[2] Initial coordinates of all compounds were extracted from the experimental single-crystal X-ray structures. Geometries were optimised at the M062X-D3/def2SVP[3-5] level of theory without symmetry constraints, and minima were confirmed by the absence of imaginary eigenvalues in the hessian matrix, by way of a frequency calculation performed at the same level of theory.

Comparison of metrical parameters for the dialumenes **I**, **II**, and **1** indicates that optimisation at the M062X-D3/def2SVP level of theory gives excellent agreement with their experimentally determined geometries (see following Tables for summary of key bond parameters). The electronic structure analysis of **1** has been performed on isomer **1C** of the major component from the crystal structure. Geometry optimisations of the other isomers (major and minor component) furnished similar structures with nearly equidistant Al–Al bonds.

Single point energy calculations were performed on the optimised geometries at the SMD-B3LYP-D3/6-311G(2d,2p)[6-8] level of theory, utilising the SMD solvation model with benzene as the solvent (ε=2.2706). The basis set superposition error in dimeric structures was corrected with the counterpoise method by Boys and Simon.[9]

The NBO program (version 6.0) was used to perform Natural Bond Orbital analyses on the optimised structures at the B3LYP-D3/6-311G(2d,2p) level of theory.[10]

The topology of the electron density was analysed using QTAIM (quantum theory of atoms in molecules), as implemented in the AIMALL package.[11]

Time dependent (TD-)DFT calculations were carried out at the SMD-B3LYP-D3/6-311G(2d,2p) level of theory, employing the Tamm-Dancoff Approximation (TDA) and employing hexane as the solvent (ε=1.8819). Natural Transition Orbital calculations were then carried out on selected excited states of the TD-DFT calculations, using the same level of theory, to provide an intuitive picture of the involved states.[12]

NMR shielding tensors were calculated at the SMD-B3LYP-D3/6-311G(2d,2p) level of theory, using the Gauge-Independent Atomic Orbital (GIAO)[13] method and employing benzene as the solvent (ε=2.2706).

Analysis of the topology of the Electron Localisation Function (ELF)[14], $\eta(r)$, was carried out using the Multiwfn program[15]. ELF basin analysis was carried out with a grid step size of 0.10 Bohr and by integrating the electron density over the basins, the average population of each basin was obtained. The sum of the synaptic valence basin populations corresponding to the Al-Al bond, $\sum_i V_i$(Al,Al) was used as an estimate of the total basin population of the Al-Al bond.

Figures of structures and isosurface plots were generated with ChemCraft (Version 1.8)[16] and UCSF Chimera (Version 1.15).[17]

Calculated structural parameters of dialumenes I, II, 1, and models M[1] – M[8].

Comparison of experimental and calculated geometries of I, II and 1

Table S2: X-ray and calculated bond parameters of dialumene **I**, NHC = Imidazol-2-ylidene ($C_3H_4N_2$).

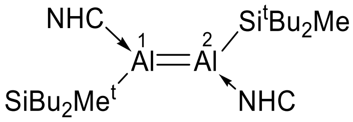

	X–Ray	DFT
Al^1–Al^2 / Å	2.394 (1)	2.372
Al^1–Si / Å	2.494 (1)	2.467
Al^1–C^{NHC} / Å	2.072 (3)	2.081
C^{NHC}–Al^1–Si / °	116.21 (9)	114.95
Si–Al^1–Al^2–Si / °	180.00 (7)	180.00
C^{NHC}–Al^1–Al^2–C^{NHC} / °	180.00 (1)	180.00
Si–Al^1–Al^2–C^{NHC} / °	1.20 (1)	10.02
θ	0.71	7.51/7.51
τ	0.00	0.00

Table S3: X-ray and calculated bond parameters of dialumene **II**, NHC = Imidazol-2-ylidene ($C_3H_4N_2$).

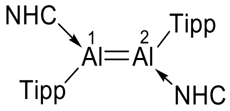

	X-Ray	DFT
Al^1–Al^2 / Å	2.404 (8)	2.373
Al^1–C^{Tipp} / Å	2.029 (2)	2.033
Al^2–C^{Tipp} / Å	2.018 (2)	2.031
Al^1–C^{NHC} / Å	2.060 (2)	2.048
Al^2–C^{NHC} / Å	2.042 (2)	2.050
C^{NHC}–Al^1–C^{Tipp} / °	110.27 (7)	111.76
C^{NHC}–Al^2–C^{Tipp} / °	112.84 (7)	109.93
C^{Tipp}–Al^1–Al^2–C^{Tipp} / °	-167.04 (9)	-171.76
C^{NHC}–Al^1–Al^2-C^{NHC} / °	-175.54 (8)	-174.56
C^{Tipp}–Al^1–Al^2–C^{NHC} / °	35.81 (9)	-13.49
C^{NHC}–Al^1–Al^2–C^{Tipp} / °	-18.39 (9)	27.17
θ	17.25/23.70	14.07/15.84
τ	12.06	7.61

Table S4: X-ray and calculated bond parameters of dialumene **1**.

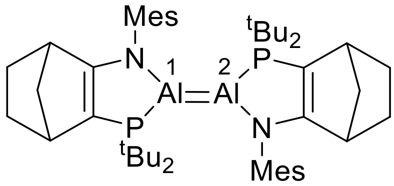

	**X-Ray**	**DFT**
Al^1–Al^2 / Å	2.519 (14)	2.514
Al^1–N / Å	1.909 (2)	1.925
Al^1–P / Å	2.482 (9)	2.523
Al^2–N / Å	-	1.924
Al^2-P / Å	-	2.504
N–Al^1–P / °	84.86 (7)	84.26
N–Al^2–P / °	-	84.10
θ	48.82	48.38/53.97
τ	0.00	0.00

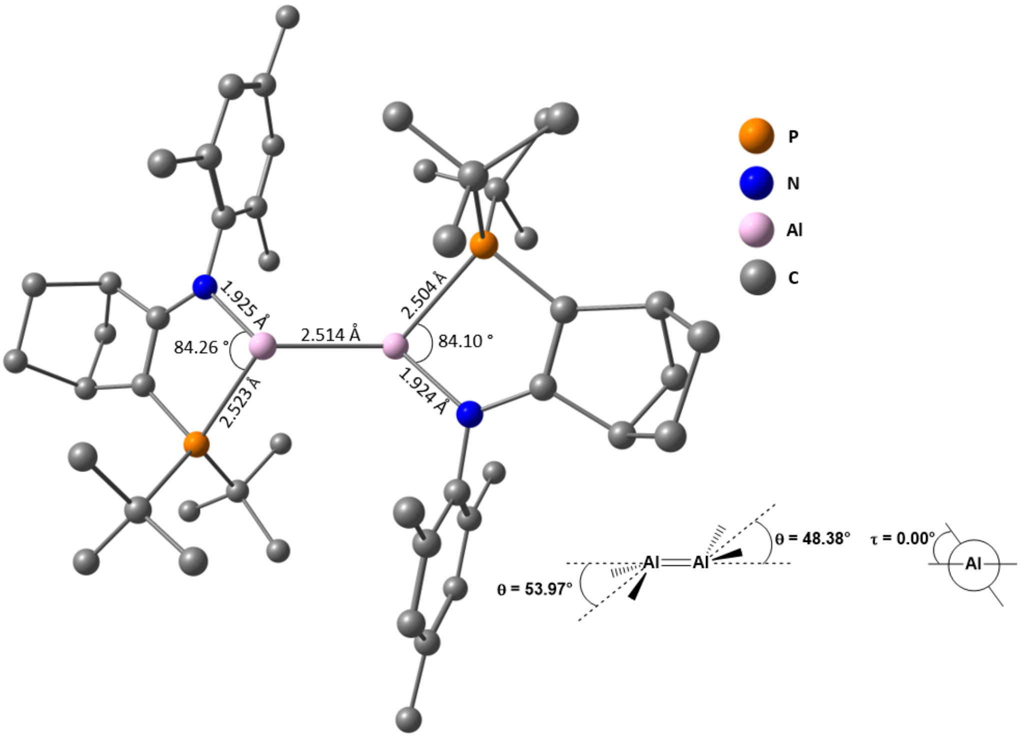

Figure S8: Calculated structure of **1** (major component, isomer C) with key structural parameters.

Model dialumenes M^1 – M^8

Table S5: Calculated bond parameters of model systems with monodentate ligands. NHC = Imidazol-2-ylidene ($C_3H_4N_2$).

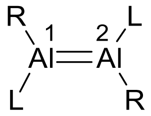

	L	R	Al^1–Al^2 / Å	R–Al–R' / °	θ(Al^1)/ θ(Al^2)	τ
**M^1**	NHC	H	2.422	101.71	29.59/47.22	17.81
**M^2**	NHC	NMe_2	2.483	91.97	43.06/43.07	0.00
**M^3**	NHC	Ph	2.444	97.10	33.37/53.92	20.54
**M^4**	NHC	$SiMe_3$	2.392	112.08/106.94	19.10/16.34	7.58
**M^5**	PMe_3	H	2.446	95.84	46.59/46.59	0.00
**M^6**	PMe_3	NMe_2	2.602	99.43	63.46/44.60	19.76
**M^7**	PMe_3	Ph	2.469	92.71	50.52/50.43	0.00
**M^8**	PMe_3	$SiMe_3$	2.425	98.72	41.55/41.02	0.00

Table S6: Calculated bond parameters of truncated model systems for **1** with bidentate ligands.

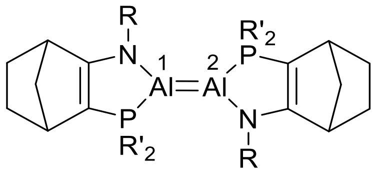

	R	R'	Al^1–Al^2 / Å	R–Al–R' / °	θ(Al^1)/ θ(Al^2)	τ
**1_{Small}**	H	H	2.643	79.72	65.66/62.53	0.00
**1_{Medium}**	Ph	Me	2.557	83.87	80.63/44.38	37.89

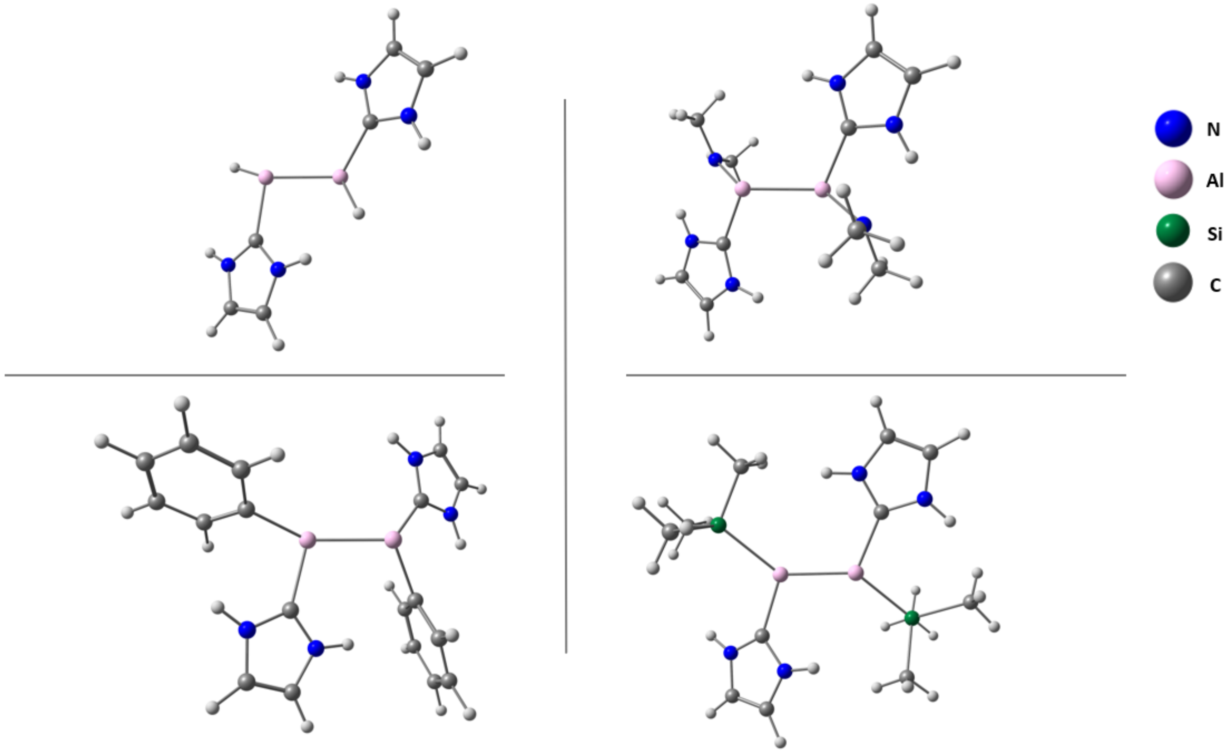

Figure S9: Optimized geometries for the model compounds **M[1]** (top left), **M[2]** (top right), **M[3]** (bottom left) and **M[4]** (bottom right) with L = Imidazol-2-ylidene ($C_3H_4N_2$) and R = H, NMe_2, Ph, $SiMe_3$ respectively.

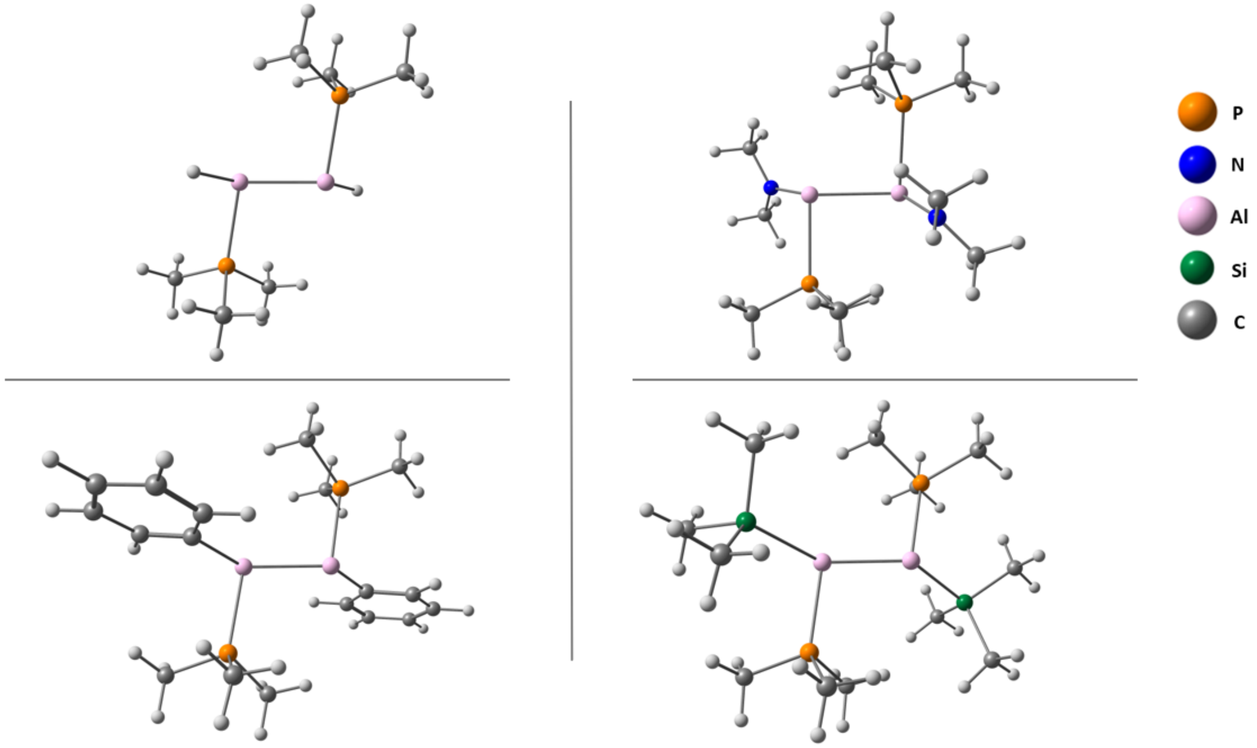

Figure S10: Optimized geometries for the model compounds **M[5]** (top left), **M[6]** (top right), **M[7]** (bottom left) and **M[8]** (bottom right) with L = PMe_3 and R = H, NMe_2, Ph, $SiMe_3$ respectively.

Calculated properties of dialumenes I, II, 1, and M[1] – M[8].

Bond dissociation energies and HOMO/LUMO gaps

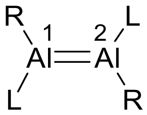

Table S7: Bond dissociation energies for dialumenes **I**, **II**, **1**, and **M[1]** – **M[8]** with and without counterpoise correction for basis set superposition error (BSSE).

	L	R	ΔG_{298} [kcal/mol] Uncorrected	ΔG_{298} [kcal/mol] BSSE-corrected	BSSE [kcal/mol]
**I**	NHC	Si^tBu_2Me	26.93	25.06	1.88
**II**	NHC	Tipp	21.38	18.95	2.42
**1**	P^tBu_2	NMes	9.39	7.09	2.29
$\mathbf{1}_{\mathbf{Small}}$	PH_2	NH	0.44	-0.90	1.35
$\mathbf{1}_{\mathbf{Medium}}$	PMe_2	NPh	11.01	8.74	2.27
**M[1]**	NHC	H	23.18	22.09	1.10
**M[2]**	NHC	NMe_2	13.19	11.46	1.74
**M[3]**	NHC	Ph	22.49	20.60	1.90
**M[4]**	NHC	$SiMe_3$	34.83	33.18	1.64
**M[5]**	PMe_3	H	20.23	19.31	0.92
**M[6]**	PMe_3	NMe_2	4.33	2.11	2.22
**M[7]**	PMe_3	Ph	21.16	19.73	1.44
**M[8]**	PMe_3	$SiMe_3$	26.96	25.50	1.47

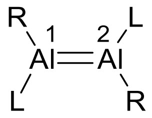

Table S8: HOMO-LUMO energy gaps of dialumenes.

	L	R	HOMO-LUMO Gap [eV]
**I**	NHC	Si^tBu_2Me	2.46
**II**	NHC	Tipp	1.95
**1**	P^tBu_2	NMes	2.34
$\mathbf{1_{Small}}$	PH_2	NH	2.60
$\mathbf{1_{Medium}}$	PMe_2	NPh	2.41
$\mathbf{M^1}$	NHC	H	2.12
$\mathbf{M^2}$	NHC	NMe_2	2.24
$\mathbf{M^3}$	NHC	Ph	2.02
$\mathbf{M^4}$	NHC	$SiMe_3$	2.06
$\mathbf{M^5}$	PMe_3	H	2.52
$\mathbf{M^6}$	PMe_3	NMe_2	2.63
$\mathbf{M^7}$	PMe_3	Ph	2.41
$\mathbf{M^8}$	PMe_3	$SiMe_3$	2.36

Properties of aluminyl fragments/monomers

Table S9: Adiabatic singlet-triplet energy gaps ($\Delta E_{S\text{-}T}$) and HOMO-LUMO gap of monomeric aluminyls.

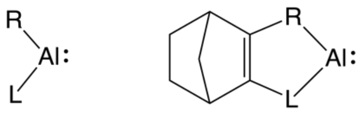

1$_{small\ /\ medium}$

	L	R	$\Delta E_{S\text{-}T}$ [kcal/mol]	HOMO-LUMO of Singlets [eV]
**I** monomer	NHC	Si^tBu_2Me	15.28	2.68
**II**monomer	NHC	Tipp	22.54	2.98
**3**	P^tBu_2	NMes	37.05	3.66
**1**Small monomer	PH_2	NH	34.29	4.04
**1**Medium monomer	PMe_2	NPh	40.28	3.67
**M^1**monomer	NHC	H	19.17	2.95
**M^2**monomer	NHC	NMe_2	25.50	3.39
**M^3**monomer	NHC	Ph	20.64	2.95
**M^4** monomer	NHC	$SiMe_3$	12.34	2.44
**M^5** monomer	PMe_3	H	23.19	3.32
**M^6** monomer	PMe_3	NMe_2	32.36	4.07
**M^7** monomer	PMe_3	Ph	25.38	3.37
**M^8** monomer	PMe_3	$SiMe_3$	16.40	2.71

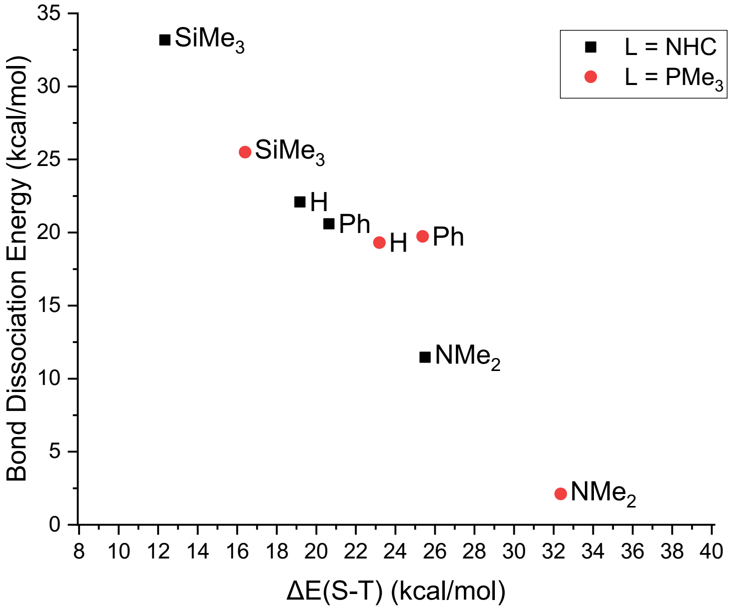

Figure S11: Plot of bond dissociation energies and singlet-triplet energy gaps of the singlet monomers ($\Delta E_{S\text{-}T}$) for model compounds $\mathbf{M^1}$-$\mathbf{M^8}$. The identity of the R substituent is noted on the plot.

Energies and interconversion of diastereomers 1A-1C

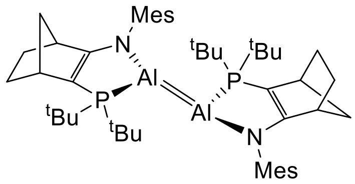

A 1.91 kcal/mol

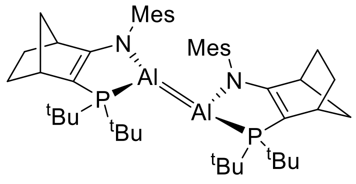

D 5.66 kcal/mol

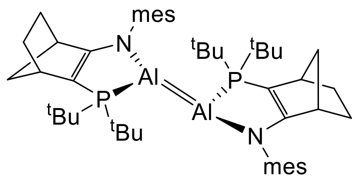

B 0.00 kcal/mol

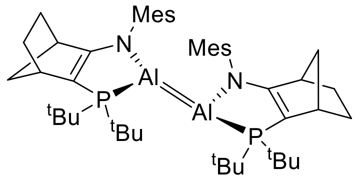

E 5.84 kcal/mol

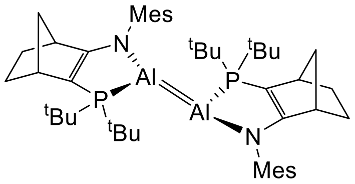

C 1.28 kcal/mol

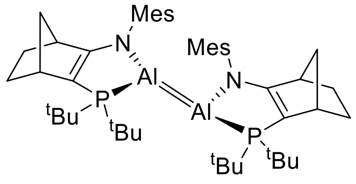

F 4.72 kcal/mol

Figure S12: Relative energies (ΔG_{298}) of diastereomers **1-A to 1-F**.

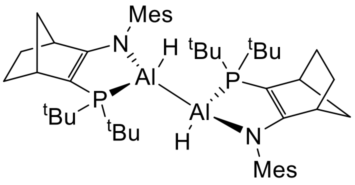

A 1.77 kcal/mol

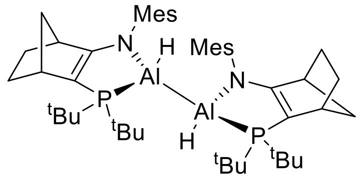

D 6.53 kcal/mol

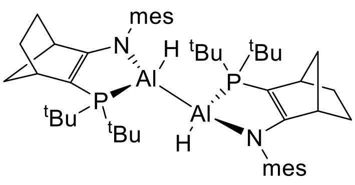

B 1.98 kcal/mol

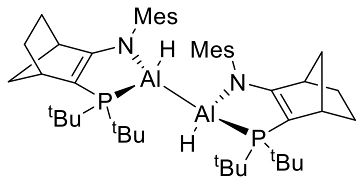

E 7.16 kcal/mol

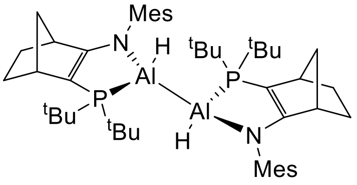

C 0.00 kcal/mol

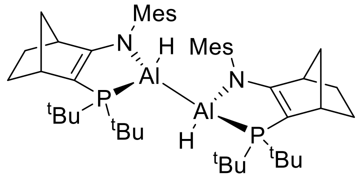

F 3.76 kcal/mol

Figure S13: Relative energies (ΔG_{298}) of diastereomers **A-F** of the Al(II) hydride **VI**.

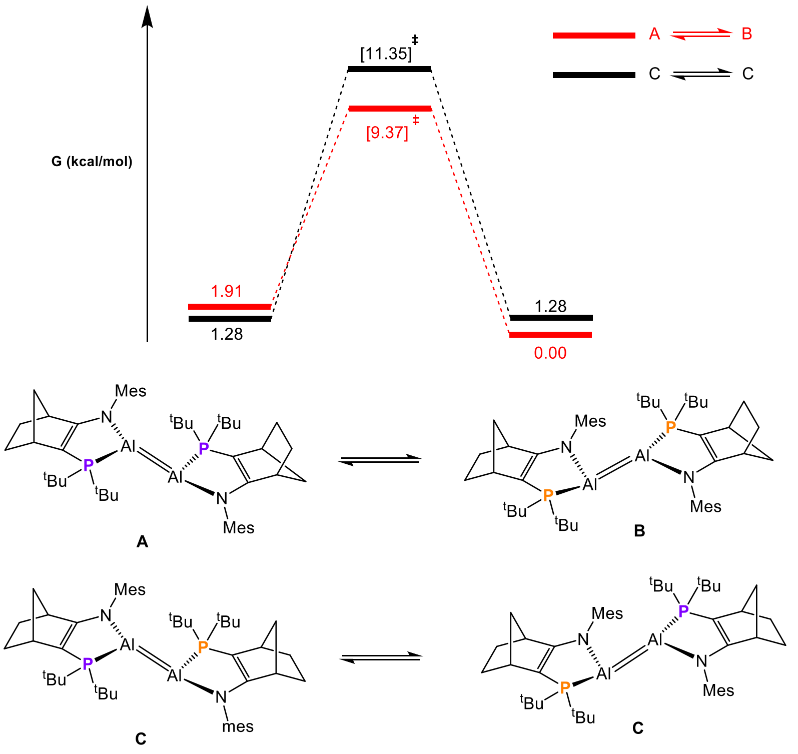

Figure S14: Reaction profiles for the interconversion of dialumene **1** diastereomers **A** to **B** and **C** to **C**, via the inversion of stereochemistry at both Al atoms.

Calculated ^{31}P NMR chemical shifts of dialumene 1 and dihydrodialane species

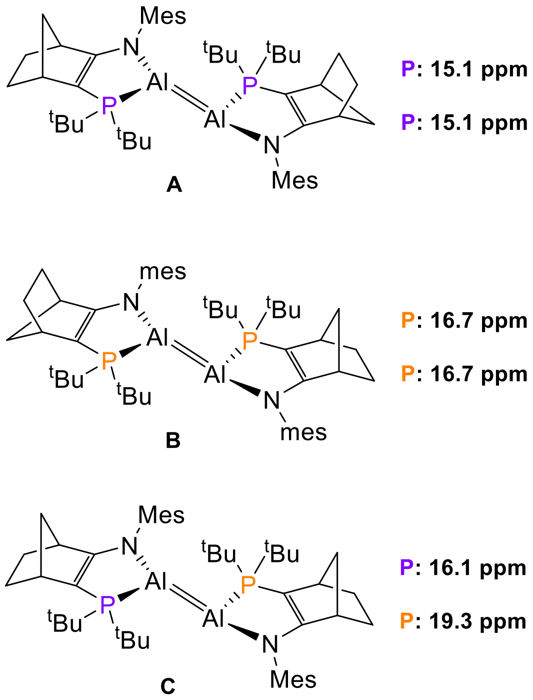

Figure S15: Calculated ^{31}P NMR shifts for diastereomers **A-C** of dialumene **1**, referenced against the magnetic shielding of H_3PO_4 ($\delta_{P,ref}$ = 0 ppm).

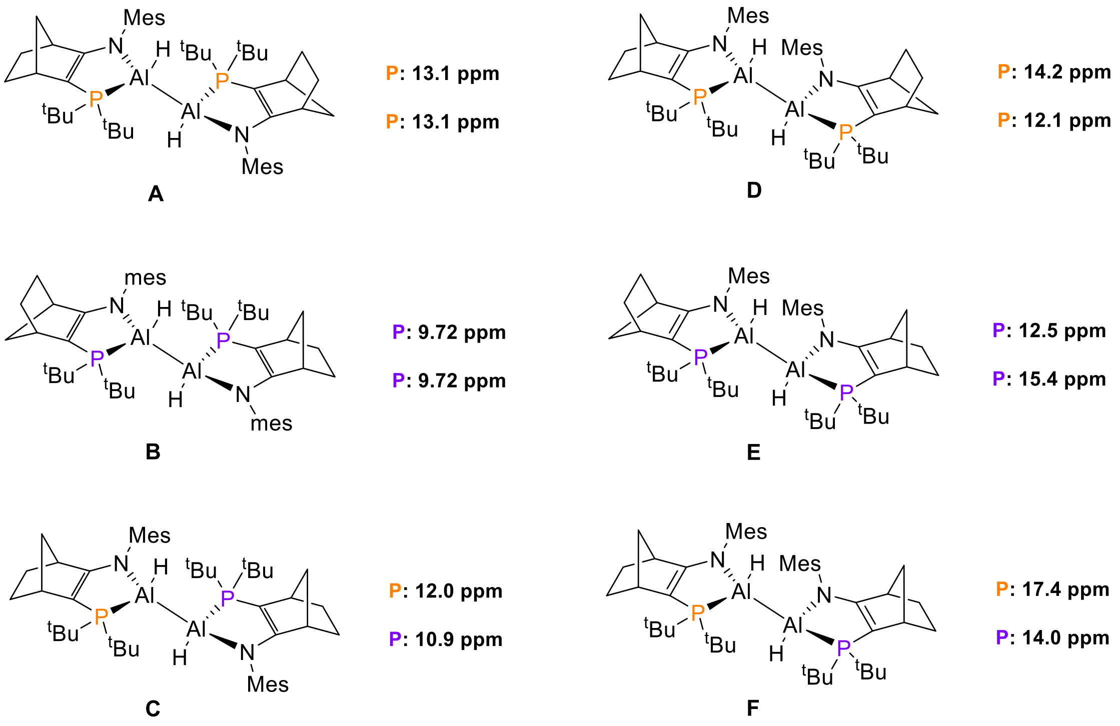

Figure S16: Calculated ^{31}P NMR shifts for diastereomers **A-F** of Al(II) hydride **VI,** referenced against the magnetic shielding of H_3PO_4 ($\delta_{P,ref}$ = 0 ppm).

Results from Time-dependent DFT calculations (TD-DFT)

Table S10: Electronic transitions of dialumenes **I**, **II**, **1** and monomeric aluminyl **3** as determined by TD-DFT (SMD-B3LYP-D3/6-311G(2d,2p)) calculations.

	**Wavelength [nm]**	**MO** Contributions	**F** (oscillator strength)
**I**	492.95	HOMO → LUMO+1<br>HOMO → LUMO+2<br>HOMO → LUMO+3	0.3496
	476.97	HOMO → LUMO+1<br>HOMO → LUMO+3	0.2006
**II**	721.58	HOMO → LUMO<br>HOMO → LUMO+1	0.2360
	679.17	HOMO → LUMO<br>HOMO → LUMO+1	0.2559
**1**	519.65	HOMO-1 → LUMO<br>HOMO → LUMO<br>HOMO → LUMO+1	0.5801
**3**	410.53	HOMO → LUMO	0.0602

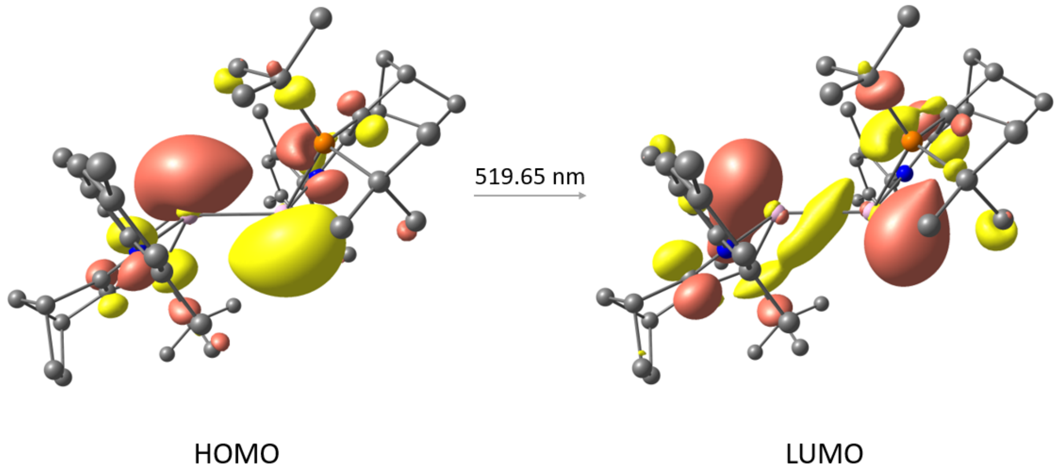

Figure S17: Natural transition orbitals (NTOs, isovalue=0.036) for the first excited state of **1**, corresponding to the dominant absorption band in the absorption spectrum. The transition has dominant HOMO-LUMO character.

Frontier molecular orbitals of 1

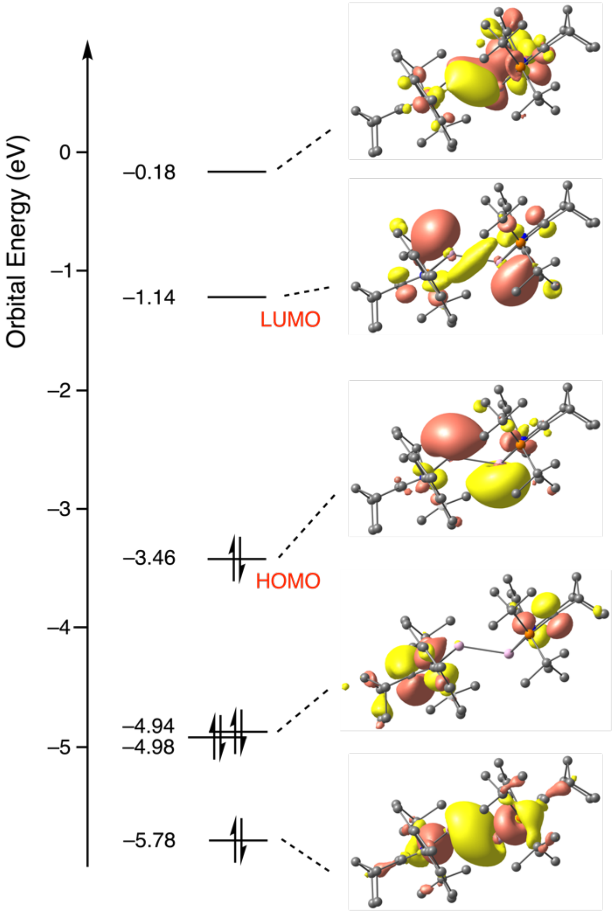

Figure S18: Molecular orbital diagram (B3LYP/6-311G(2d,2p)) of the canonical frontier orbitals of **1** (isovalue=0.036).

Natural Bond Orbital Analyses

Table S11: NBO analysis of dialumenes **I** and **II**.

	Bond	NPA Charge	Occupancy	Pol.	Hybrid.	WBI
**I**	Al^1–Al^2	0.08 (Al^1)<br>0.08 (Al^2)	1.92	50% (Al^1)<br>50% (Al^2)	$sp^{1.11}$ (Al^1)<br>$sp^{1.11}$ (Al^2)	1.67
	Al^1–Al^2	0.08 (Al^1)<br>0.08 (Al^2)	1.76	50% (Al^1)<br>50% (Al^2)	$sp^{99.99}$ (Al^1)<br>$sp^{99.99}$ (Al^2)	1.67
	Al^1–C^{NHC}	0.05 (C^{NHC})	1.94	14% (Al^1)<br>86% (C^{NHC})	$sp^{4.82}$ (Al^1)<br>$sp^{1.27}$ (C^{NHC})	0.49
	Al^1–Si	1.17 (Si)	1.93	36% (Al^1)<br>64% (Si)	$sp^{1.82}$ (Al^1)<br>$sp^{1.97}$ (Si)	0.88
**II**	Al^1–Al^2	0.49 (Al^1)<br>0.49 (Al^2)	1.93	50% (Al^1)<br>50% (Al^2)	$sp^{1.03}$ (Al^1)<br>$sp^{1.06}$ (Al^2)	1.54
	Al^1–Al^2	0.49 (Al^1)<br>0.49 (Al^2)	1.60	50% (Al^1)<br>50% (Al^2)	$sp^{99.99}$ (Al^1)<br>$sp^{43.39}$ (Al^2)	1.54
	Al^1–C^{NHC}	-0.01 (C^{NHC})	1.94	14% (Al^1)<br>86% (C^{NHC})	$sp^{3.66}$ (Al^1)<br>$sp^{1.27}$ (C^{NHC})	0.55
	Al^1–C^{Tipp}	-0.57 (C^{Tipp})	1.92	16% (Al^1)<br>84% (C^{Tipp})	$sp^{2.49}$ (Al^1)<br>$sp^{2.31}$ (C^{Tipp})	0.52
	Al^2–C^{NHC}	0.00 (C^{NHC})	1.94	15% (Al^2)<br>85% (C^{NHC})	$sp^{3.73}$ (Al^2)<br>$sp^{1.28}$ (C^{NHC})	0.57
	Al^2–C^{Tipp}	-0.56 (C^{Tipp})	1.92	17% (Al^1)<br>83% (C^{Tipp})	$sp^{2.52}$ (Al^2)<br>$sp^{2.34}$ (C^{Tipp})	0.52

Table S12: NBO analysis of dialumenes **1**, **TS$_{1C-1C}$** and dihydrodialane **VI**.

	Bond	NPA Charge	Occupancy	Pol.	Hybrid.	WBI
**1**	Al^1–Al^2	0.56 (Al^1)<br>0.66 (Al^2)	1.77	51% (Al^1)<br>49% (Al^2)	$sp^{1.22}$ (Al^1)<br>$sp^{1.24}$ (Al^2)	1.31
	Al^1–Al^2	0.56 (Al^1)<br>0.66 (Al^2)	1.69	51% (Al^1)<br>49% (Al^2)	$sp^{4.14}$ (Al^1)<br>$sp^{3.49}$ (Al^2)	1.31
	Al^1–P	0.90 (P)	1.91	12% (Al^1)<br>88% (P)	$sp^{6.87}$ (Al^1)<br>$sp^{2.06}$ (P)	0.42
	Al^1–N	-0.93 (N)	1.92	6% (Al^1)<br>94% (N)	$sp^{3.19}$ (Al^1)<br>$sp^{2.72}$ (N)	0.23
	Al^2–P	0.88 (P)	1.91	12% (Al^2)<br>88% (P)	$sp^{6.03}$ (Al^2)<br>$sp^{2.12}$ (P)	0.40
	Al^2–N	-0.94	1.91	5% (Al^1)<br>95% (N)	$sp^{3.97}$ (Al^2)<br>$sp^{2.79}$ (N)	0.21
**TS$_{1C-1C}$**	Al^1–Al^2	0.45 (Al^1)<br>0.50 (Al^2)	1.97	50% (Al^1)<br>50% (Al^2)	$sp^{0.73}$ (Al^1)<br>$sp^{0.75}$ (Al^2)	1.68
	Al^1–Al^2	0.45 (Al^1)<br>0.50 (Al^2)	1.75	49% (Al^1)<br>51% (Al^2)	$sp^{99.99}$ (Al^1)<br>$sp^{99.39}$ (Al^2)	1.68
**VI**	Al^1–Al^2	0.90 (Al^1)<br>0.91 (Al^2)	1.92	50% (Al^1)<br>50% (Al^2)	$sp^{1.51}$ (Al^1)<br>$sp^{1.52}$ (Al^2)	0.91
	Al^1–H	-0.45 (H)	1.95	27% (Al^1)<br>73% (H)	$sp^{2.43}$ (Al^1)<br>$sp^{0.01}$ (H)	0.72
	Al^2–H	-0.45 (H)	1.95	26% (Al^2)<br>74% (H)	$sp^{2.44}$ (Al^1)<br>$sp^{0.01}$ (H)	0.72
	Al^1–P	0.97 (P)	1.93	17% (Al^2)<br>83% (P)	$sp^{5.88}$ (Al^1)<br>$sp^{2.14}$ (P)	0.52
	Al^1–N	-0.88 (N)	1.92	8% (Al^2)<br>92% (N)	$sp^{4.91}$ (Al^1)<br>$sp^{2.37}$ (N)	0.28
	Al^2–P	0.97 (P)	1.92	17% (Al^2)<br>83% (P)	$sp^{5.58}$ (Al^1)<br>$sp^{2.18}$ (P)	0.52
	Al^2–N	-0.89 (N)	1.92	8% (Al^2)<br>92% (N)	$sp^{5.04}$ (Al^1)<br>$sp^{2.39}$ (N)	0.27

Table S13: NLMO Analysis of dialumenes **I** and **II**.

	Bond	Occupancy	Pol.	Hybridisation
**I**	Al^1–Al^2	2.00	48% (Al^1)<br>48% (Al^2)	$sp^{0.86}$ s(54%) p(46%) (Al^1)<br>$sp^{0.86}$ s(54%) p(46%) (Al^2)
	Al^1–Al^2	2.00	44% (Al^1)<br>44% (Al^2)	$sp^{99.99}$ s(0%) p(100%) (Al^1)<br>$sp^{99.99}$ s(0%) p(100%) (Al^2)
	Al^1–C^{NHC}	2.00	14% (Al^1)<br>83% (C^{NHC})	$sp^{2.76}$ s(27%) p(73%) (Al^1)<br>$sp^{1.40}$ s(42%) p(58%) (C^{NHC})
	Al^1–Si	2.00	35% (Al^1)<br>62% (Si)	$sp^{1.26}$ s(44%) p(56%) (Al^1)<br>$sp^{1.49}$ s(40%) p(60%) (Si)
	Al^2–C^{NHC}	2.00	14% (Al^2)<br>83% (C^{NHC})	$sp^{2.76}$ s(27%) p(73%) (Al^2)<br>$sp^{1.40}$ s(42%) p(58%) (C^{NHC})
	Al^2–Si	2.00	35% (Al^2)<br>62% (Si)	$sp^{1.26}$ s(44%) p(56%) (Al^2)<br>$sp^{1.49}$ s(40%) p(60%) (Si)
**II**	Al^1–Al^2	2.00	49% (Al^1)<br>49% (Al^2)	$sp^{0.74}$ s(57%) p(42%) (Al^1)<br>$sp^{0.78}$ s(56%) p(44%) (Al^2)
	Al^1–Al^2	2.00	40% (Al^1)<br>40% (Al^2)	$sp^{48.34}$ s(2%) p(98%) (Al^1)<br>$sp^{23.32}$ s(4%) p(96%) (Al^2)
	Al^1–C^{NHC}	2.00	15% (Al^1)<br>83% (C^{NHC})	$sp^{1.99}$ s(33%) p(66%) (Al^1)<br>$sp^{1.40}$ s(42%) p(58%) (C^{NHC})
	Al^1–C^{Tipp}	2.00	16% (Al^1)<br>80% (C^{Tipp})	$sp^{1.44}$ s(41%) p(59%) (Al^1)<br>$sp^{2.61}$ s(28%) p(72%) (C^{Tipp})
	Al^2–C^{NHC}	2.00	15% (Al^2)<br>83% (C^{NHC})	$sp^{2.06}$ s(33%) p(67%) (Al^2)<br>$sp^{1.41}$ s(42%) p(58%) (C^{NHC})
	Al^2–C^{Tipp}	2.00	16% (Al^1)<br>80% (C^{Tipp})	$sp^{1.47}$ s(40%) p(59%) (Al^2)<br>$sp^{2.61}$ s(28%) p(72%) (C^{Tipp})

Table S14: NLMO Analysis of dialumenes **1**, **$TS_{1C\text{-}1C}$** and dihydrodialane **VI**.

	Bond	Occupancy	Pol.	Hybridisation
**1**	Al^1–Al^2	2.00	50% (Al^1)<br>48% (Al^2)	$sp^{0.34}$ s(74%) p(26%) (Al^1)<br>$sp^{0.33}$ s(75%) p(25%) (Al^2)
	Al^1–Al^2	2.00	46% (Al^1)<br>45% (Al^2)	$sp^{1.25}$ s(44%) p(55%) (Al^1)<br>$sp^{1.09}$ s(48%) p(52%) (Al^2)
	Al^1–P	2.00	12% (Al^1)<br>84% (P)	$sp^{3.09}$ s(24%) p(75%) (Al^1)<br>$sp^{1.84}$ s(35%) p(65%) (P)
	Al^1–N	2.00	6% (Al^1)<br>90% (N)	$sp^{1.55}$ s(39%) p(60%) (Al^1)<br>$sp^{2.84}$ s(26%) p(74%) (N)
	Al^2–P	2.00	12% (Al^2)<br>84% (P)	$sp^{2.62}$ s(27%) p(72%) (Al^2)<br>$sp^{1.92}$ s(34%) p(66%) (P)
	Al^2–N	2.00	5% (Al^1)<br>91% (N)	$sp^{1.81}$ s(35%) p(63%) (Al^2)<br>$sp^{2.86}$ s(26%) p(74%) (N)
**$TS_{1C\text{-}1C}$**	Al^1–Al^2	2.00	49% (Al^1)<br>50% (Al^2)	$sp^{0.59}$ s(63%) p(37%) (Al^1)<br>$sp^{0.61}$ s(62%) p(38%) (Al^2)
	Al^1–Al^2	2.00	43% (Al^1)<br>44% (Al^2)	$sp^{99.99}$ s(0%) p(100%) (Al^1)<br>$sp^{99.99}$ s(0%) p(100%) (Al^2)
**VI**	Al^1–Al^2	2.00	49% (Al^1)<br>48% (Al^2)	$sp^{0.99}$ s(50%) p(50%) (Al^1)<br>$sp^{0.93}$ s(51%) p(48%) (Al^2)
	Al^1–H	2.00	27% (Al^1)<br>72% (H)	$sp^{1.29}$ s(44%) p(56%) (Al^1)<br>$sp^{0.01}$ s(99%) p(1%) (H)
	Al^2–H	2.00	26% (Al^2)<br>72% (H)	$sp^{1.35}$ s(43%) p(57%) (Al^1)<br>$sp^{0.01}$ s(99%) p(1%) (H)
	Al^1–P	2.00	17% (Al^2)<br>80% (P)	$sp^{2.77}$ s(27%) p(73%) (Al^1)<br>$sp^{1.89}$ s(35%) p(65%) (P)
	Al^1–N	2.00	89% (Al^2)<br>8% (N)	$sp^{2.03}$ s(33%) p(66%) (Al^1)<br>$sp^{2.60}$ s(28%) p(72%) (N)
	Al^2–P	2.00	17% (Al^2)<br>80% (P)	$sp^{2.54}$ s(28%) p(71%) (Al^1)<br>$sp^{1.96}$ s(34%) p(66%) (P)
	Al^2–N	2.00	8% (Al^2)<br>89% (N)	$sp^{2.15}$ s(31%) p(67%) (Al^1)<br>$sp^{2.61}$ s(28%) p(72%) (N)

Table S15: Second order perturbation theory analysis. [a] NHC bonded to Al^1. [b] NHC bonded to Al^2

	Donor NBO Occupancy	Acceptor NBO Occupancy	Energy [kcal/mol]
**II**	π (Al^1–Al^2) (1.60)	π* (C^{NHC}–N^{NHC}) (0.56)[a]	37.38
	π (Al^1–Al^2) (1.60)	π* (C^{NHC}–N^{NHC}) (0.56)[b]	39.00
**1**	σ (Al^1–Al^2) (1.77)	σ* (Al^1–N) (0.12)	13.98
	σ (Al^1–Al^2) (1.77)	π* (Al^1–Al^2) (0.16)	16.13
	σ (Al^1–Al^2) (1.77)	σ* (Al^2–N) (0.10)	13.53
	π (Al^1–Al^2) (1.69)	σ* (Al^1–Al^2) (0.13)	31.32

Table S16: NBO Analysis of aluminyl monomers (singlet ground state).

	Bond	NPA Charge	Occupancy	Pol.	Hybrid.	WBI
**$I_{monomer}$**	LP Al^1	0.41 (Al^1)	1.86	-	$sp^{0.18}$ (Al^1)	-
	Al^1–Si	1.04 (Si)	1.85	26% (Al^1)<br>74% (Si)	$sp^{8.72}$ (Al^1)<br>$sp^{1.97}$ (Si)	0.71
	Al^1–C^{NHC}	-0.04 (C^{NHC})	1.94	9% (Al^1)<br>91% (C^{NHC})	$sp^{15.94}$ (Al^1)<br>$sp^{1.37}$ (C^{NHC})	0.45
**$II_{monomer}$**	LP Al^1	0.56 (Al^1)	1.91	-	$sp^{0.20}$ (Al^1)	-
	Al^1–C^{Tipp}	-0.58 (C^{Tipp})	1.92	13% (Al^1)<br>87% (C^{Tipp})	$sp^{8.16}$ (Al^1)<br>$sp^{2.62}$ (C^{Tipp})	0.45
	Al^1–C^{NHC}	0.03 (C^{NHC})	1.94	10% (Al^1)<br>90% (C^{NHC})	$sp^{15.15}$ (Al^1)<br>$sp^{1.39}$ (C^{NHC})	0.40
**3**	LP Al^1	0.68 (Al^1)	1.96	-	$sp^{0.09}$ (Al^1)	-
	Al^1–P	0.89 (P)	1.91	9% (Al^1)<br>91% (P)	$sp^{22.59}$ (Al^1)<br>$sp^{2.37}$ (P)	0.30
	Al^1–N	-0.98 (N)	1.93	4% (Al^1)<br>96% (N)	$sp^{16.01}$ (Al^1)<br>$sp^{2.86}$ (N)	0.25

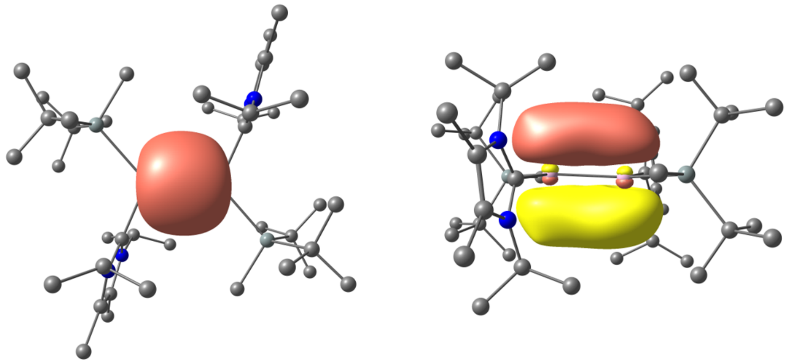

σ (Al¹-Al²) π (Al¹-Al²)

Figure S19: Key NLMOs of dialumene **I** (isovalue=0.036).

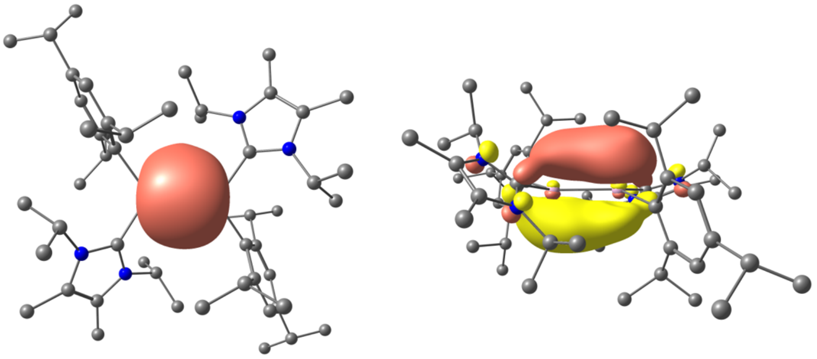

σ (Al¹-Al²) π (Al¹-Al²)

Figure S20: Key NLMOs of dialumene **II** (isovalue=0.036).

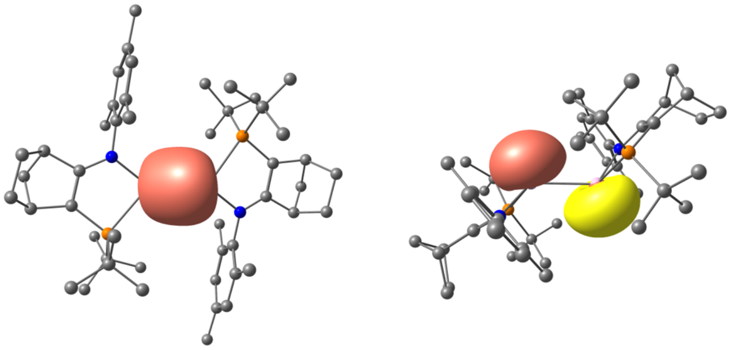

σ (Al¹-Al²) π (Al¹-Al²)

Figure S21: Key NLMOs of dialumene **1** (isovalue=0.036).

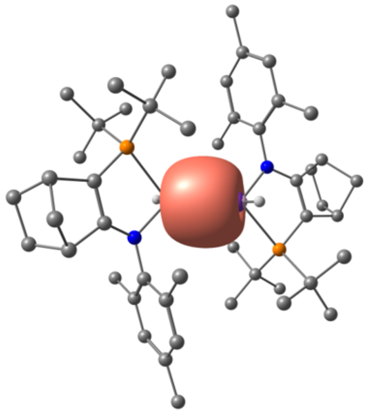

Figure S22: Key NLMO of dihydrodialane **VI** (**S2**) (isovalue=0.036).

Table S17: Comparison of Al–Al bond lengths, Natural Population Analysis Charges and Wiberg Bond Indices (WBI) for the three dialumenes **1**, **I**, and **II**, dihydrodialane **VI**. Structures **I, II** and **TS$_{1C-1C}$** as well as **VI** serve as reference and provide numerical values for the upper bound (double bond) and lower bound (single bond).

	**Al–Al / Å Exp.**	**Al–Al / Å Calc.**	**Al^1/Al^2 NPA charge**	**WBI**
**I**	2.394	2.372	0.08/0.08	1.67
**II**	2.404	2.373	0.49/0.49	1.54
**1**	2.519	2.514	0.56/0.66	1.31
**TS1$_{C-1C}$**	–	2.315	0.45/0.50	1.68
**VI**	2.659	2.565	0.90/0.91	0.91

ELF Analysis

Table S18: Valence basin population values (e) for dialumenes **I, II**, and **1**, and dihydrodialane **VI**. [a]Monosynaptic basins on each Al centre (all other basins are disynaptic).

	Bond (A–B)	V_1(A,B)	V_2(A,B)	V_3(A,B)	V_1(A,B) ∪ V_2(A,B)	V_1(A,B) ∪ V_2(A,B) ∪ V_3(A,B)
**I**	Al^1–Al^2	1.71	1.71	-	3.41	-
	Al^1–C^{NHC}	2.59	-	-	-	-
	Al^1–Si	2.12	-	-	-	-
	Al^2–C^{NHC}	2.59	-	-	-	-
	Al^2–Si	2.12	-	-	-	-
**II**	Al^1–Al^2	1.68	1.68	-	3.36	-
	Al^1–C^{NHC}	2.70	-	-	-	-
	Al^1–C^{Tipp}	2.39	-	-	-	-
	Al^2–C^{NHC}	2.70	-	-	-	-
	Al^2–C^{Tipp}	2.38	-	-	-	-
**1**	Al^1–Al^2	1.11	1.32[a]	1.33[a]	-	3.76
	Al^1–P	2.14	-	-	-	-
	Al^1–N	3.81	-	-	-	-
	Al^2–P	2.13	-	-	-	-
	Al^2–N	3.82	-	-	-	-
**VI**	Al^1–Al^2	1.96	-	-	-	-

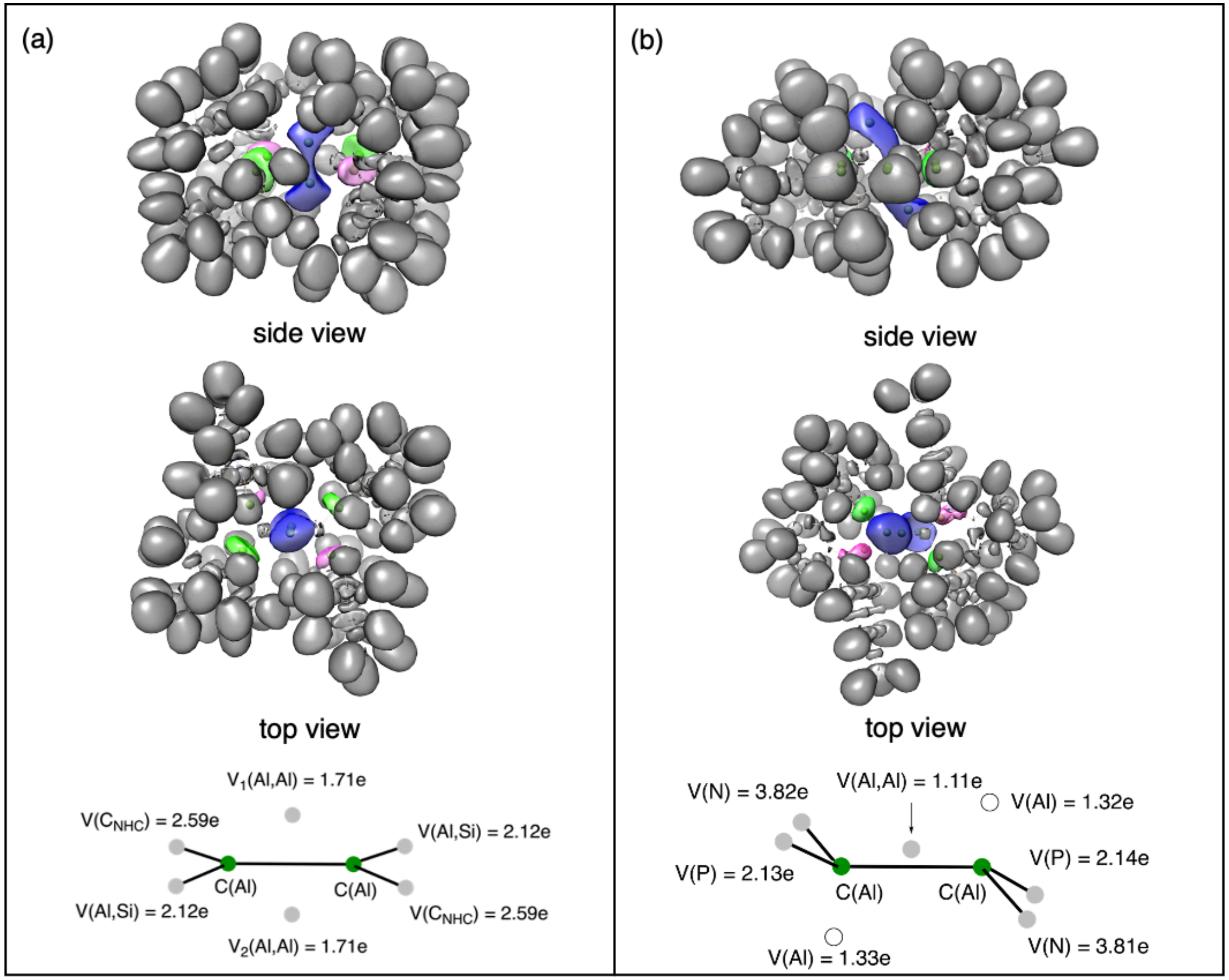

Figure S23: Plots of full ELF localisation domains (isovalue=0.795) along with key attractors of the Al_2 core and their basin populations for dialumenes (a) **I** and (b) **1**. The Al=Al bond in **I** is characterised by the presence of two disynaptic attractors $V_{i=1,2}$(Al,Al) on each side of the Al-Al bond. Their combined disynaptic basin populations (3.44e) support the notion of a covalent double-bond consisting of a σ- and π-component.[18] Color code for basins: blue Al, grey C, and H, green P or Si, pink N or C_{NHC}. Core attractors are indicated by green filled circles, di- and monosynaptic attractors are shown as grey filled circles and non-bonded/lonepair attractors are shown as open circles.

QTAIM Analysis

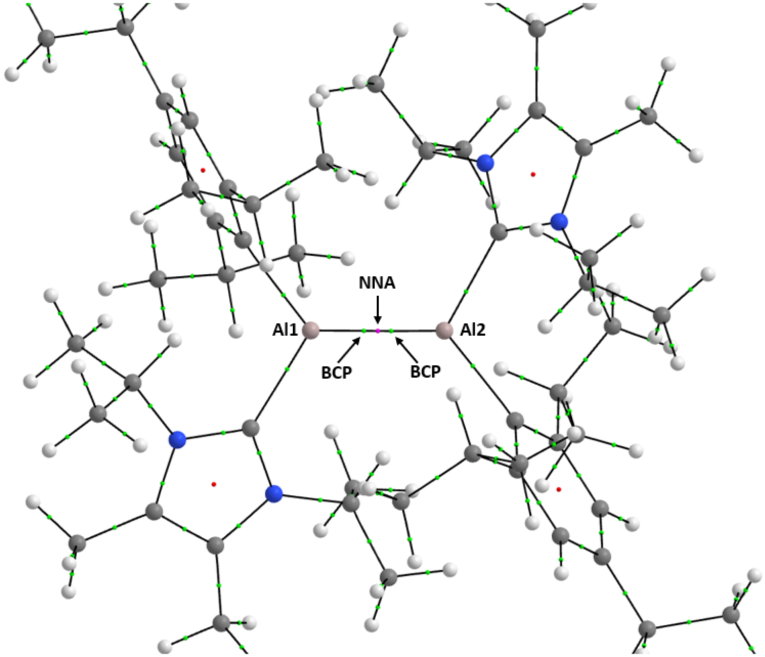

Figure S24: Molecular graph for dialumene **II**.

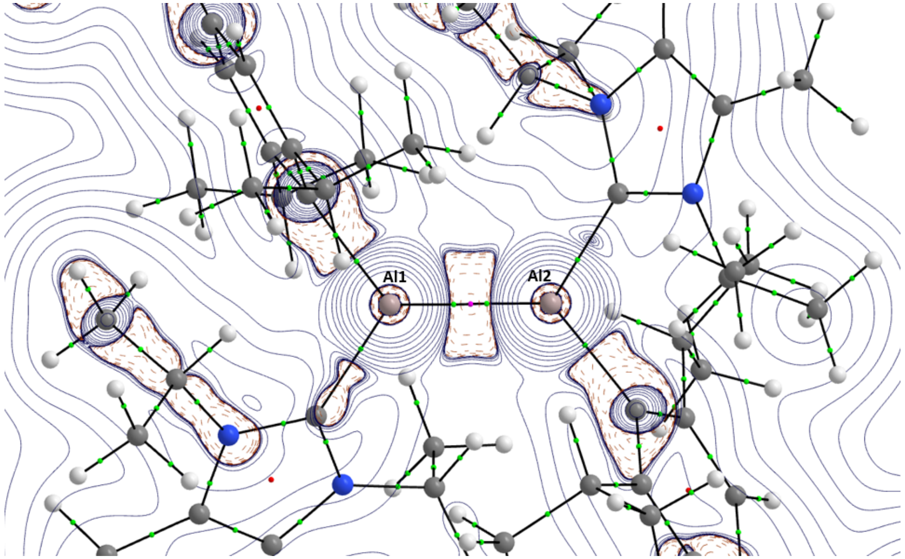

Figure S25: Laplacian $\nabla^2\rho(r)$ of the electron density in the plane containing Al1 and Al2 for dialumene **II**.

Table S19: Selected properties of the Bond Critical Points of dialumene **II**. $\rho(r)$ is the electron density (e/a_0^3), $\nabla^2\rho(r)$ is the Laplacian of the electron density (e/a_0^5), λ_n are eigenvalues of the Hessian of $\rho(r)$ (e/a_0^5), ε is the bond ellipticity defined as $(\lambda_1/\lambda_2)–1$, V(r), G(r) and H(r) represent the potential, kinetic and total energy density, respectively (Hartree/a_0^3), |V(r)|/G(r) is the ratio of potential to kinetic energy and δ(A,B) is the delocalisation index. [a]The presence of a non-nuclear attractor (NNA) and associated basin at the midpoint of the Al–Al bond complicates the analysis of the QTAIM parameters. In order to calculate the bond delocalisation index between the two Al atoms, one defines an effective delocalisation index by dividing the NNA basin equally between the two Al basins. Thus, if the two surrounding aluminum atoms are indicated by 1 and 2 and with the definitions of the localisation (λ) and delocalisation indices (δ) one finds: δ(Al1,Al2)eff = δ(Al1,Al2) + 0.5•δ(Al1,NNA) + 0. 5•δ(A2,NNA) + 0.5•λ(NNA).

**BCP**	**ρ(r)**	**$\nabla^2\rho$(r)**	**λ_1**	**λ_2**	**λ_3**	**ε**	**V(r)**	**G(r)**	**H(r)**	**|V(r)|/G(r)**	**δ(A,B)**
NNA	+0.063	-0.087	-0.049	-0.029	-0.009	NA	-0.037	+0.008	-0.029	+4.625	0.985[a]
Al1–NNA	+0.063	-0.045	-0.051	-0.028	+0.033	+0.804	-0.052	+0.020	+0.752	+2.561	0.432
Al2–NNA	+0.062	-0.044	-0.051	-0.029	+0.035	+0.776	-0.052	+0.020	+0.724	+2.538	0.428
Al1–C_{NHC}	+0.066	+0.235	-0.092	-0.080	+0.407	+0.143	-0.092	+0.075	+0.051	+1.223	0.388
Al2–C_{NHC}	+0.067	+0.236	-0.092	-0.081	+0.409	+0.134	-0.093	+0.076	+0.041	+1.223	0.379
Al1–C_{Tipp}	+0.075	+0.216	-0.097	-0.095	+0.408	+0.029	-0.104	+0.079	-0.075	+1.316	0.399
Al2–C_{Tipp}	+0.075	+0.218	-0.098	-0.094	+0.410	+0.039	-0.104	+0.079	-0.065	+1.314	0.398

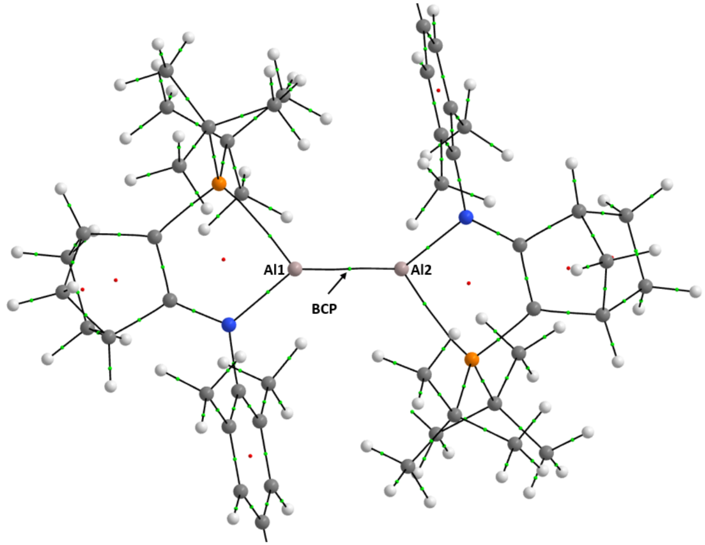

Figure S26: Molecular graph for **1**.

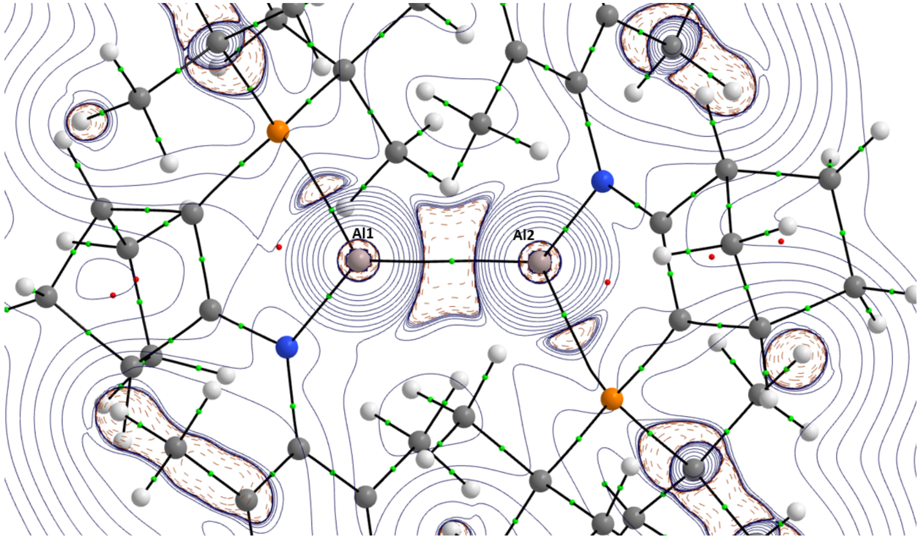

Figure S27: Laplacian $\nabla^2\rho$(r) of the electron density in the plane containing Al1 and Al2 for **1**.

Table S20: Selected properties of the Bond Critical Points of **1**. $\rho(r)$ is the electron density (e/a_0^3), $\nabla^2\rho$(r) is the Laplacian of the electron density (e/a_0^5), λ_n are eigenvalues of the Hessian of ρ(r) (e/a_0^5), ε is the bond ellipticity defined as (λ_1/λ_2)–1, V(r), G(r) and H(r) represent the potential, kinetic and total energy density, respectively (Hartree/a_0^3), |V(r)|/G(r) is the ratio of potential to kinetic energy and δ(A,B) is the delocalisation index.

**BCP**	$\rho(r)$	$\nabla^2\rho(r)$	λ_1	λ_2	λ_3	ε	**V(r)**	**G(r)**	**H(r)**	**|V(r)|/G(r)**	δ**(A,B)**
Al1–Al2	+0.052	-0.058	-0.037	-0.031	+0.011	+0.195	-0.025	+0.005	-0.020	+4.834	0.803
Al1–N	+0.074	+0.349	-0.115	-0.103	+0.567	+0.112	-0.115	+0.101	-0.014	+1.138	0.383
Al2–N	+0.074	+0.349	-0.114	-0.103	+0.566	+0.111	-0.115	+0.101	-0.014	+1.138	0.388
Al1–P	+0.046	+0.049	-0.041	-0.038	+0.129	+0.081	-0.044	+0.028	-0.016	+1.567	0.361
Al2–P	+0.048	+0.054	-0.044	-0.041	+0.139	+0.062	-0.047	+0.030	-0.017	+1.556	0.364

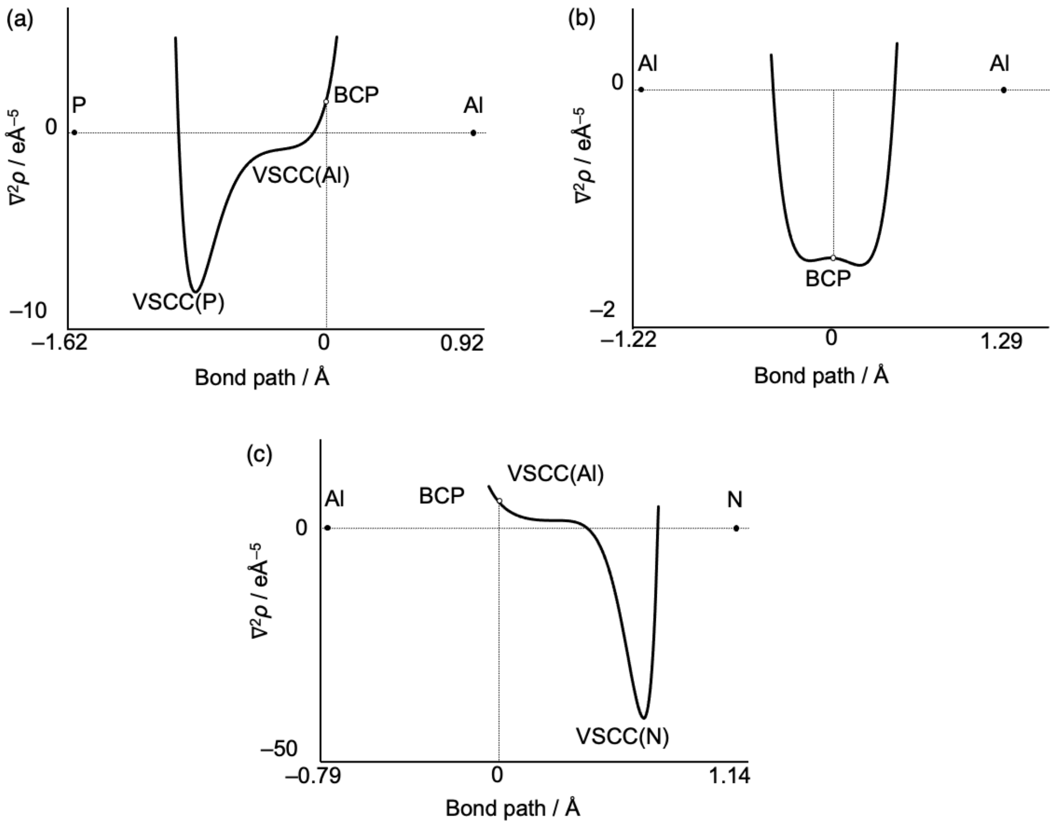

Figure S28: 1D-Laplacian contour plots along the (a) Al–P (b) Al–Al and (c) Al–N bonds in **1**.

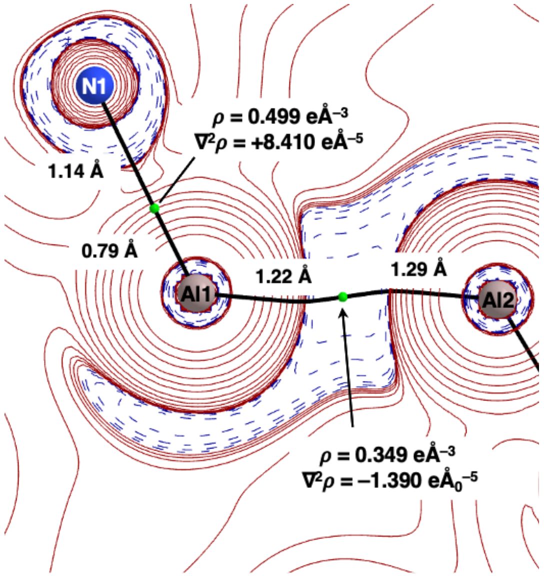

Figure S29: Laplacian $\nabla^2\rho(r)$ of the electron density in the plane containing Al1, Al2 and N1 for **1**.

Plots of the Laplacian along bond paths containing the Al centres are shown in Figure S28. Along the Al–P bond path, a distinct minimum in the 1D plot of the Laplacian can be identified corresponding to the valence shell charge concentration (VSCC) of the P atom. Located in the same atomic basin is a second, shallower region corresponding to the VSCC of the Al centre. A similar situation is also found for the Al–N bond, and in both cases this pattern is diagnostic of dative character of the Al–N and Al–P bonds. In contrast, a continuous region of charge concentration ($\nabla^2\sigma_{bcp} < 0$) is symmetrically arranged around the bcp near the midpoint of the Al–Al bond.

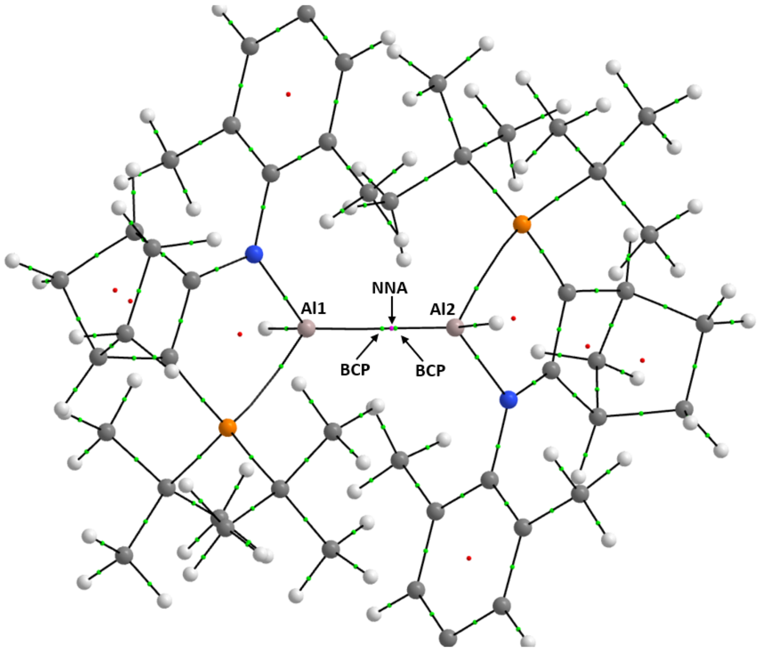

Figure S30: Molecular graph for dihydrodialane **VI**.

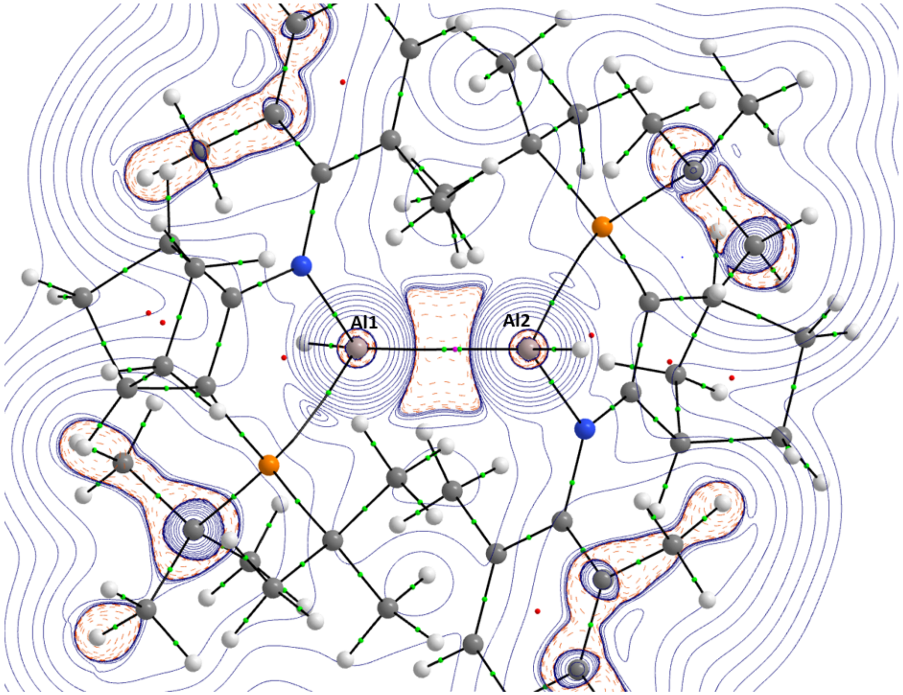

Figure S31: Laplacian $\nabla^2\rho(r)$ of the electron density in the plane containing Al1 and Al2 for dihydrodialane **VI**.

Table S21: Selected properties of the Bond Critical Points of dihydrodialane **VI**. $\rho(r)$ is the electron density (e/a_0^3), $\nabla^2\rho$(r) is the Laplacian of the electron density (e/a_0^5), λ_n are eigenvalues of the Hessian of ρ(r) (e/a_0^5), ε is the bond ellipticity defined as (λ_1/λ_2)–1, V(r), G(r) and H(r) represent the potential, kinetic and total energy density, respectively (Hartree/a_0^3), |V(r)|/G(r) is the ratio of potential to kinetic energy and δ(A,B) is the delocalisation index. [a]The presence of a non-nuclear attractor (NNA) and associated basin at the midpoint of the Al–Al bond complicates the analysis of the QTAIM parameters. In order to calculate the bond delocalisation index between the two Al atoms, one defines an *effective* delocalisation index by dividing the NNA basin equally between the two Al basins. Thus, if the two surrounding aluminum atoms are indicated by 1 and 2 and with the definitions of the localisation (λ) and delocalisation indices (δ) one finds: $\delta(\text{Al1,Al2})_{\text{eff}} = \delta(\text{Al1,Al2}) + 0.5\cdot\delta(\text{Al1,NNA}) + 0.5\cdot\delta(\text{A2,NNA}) + 0.5\cdot\lambda(\text{NNA})$.

BCP	ρ(r)	$\nabla^2\rho$(r)	λ_1	λ_2	λ_3	ε	V(r)	G(r)	H(r)	|V(r)|/G(r)	δ(A,B)
NNA	+0.060	-0.087	-0.044	-0.041	-0.002	NA	-0.030	+0.004	-0.026	+7.500	0.655[a]
Al1–NNA	+0.060	-0.083	-0.045	-0.042	+0.003	+0.080	-0.035	+0.007	-0.028	+4.898	0.209
Al2–NNA	+0.060	-0.084	-0.044	-0.041	+0.002	+0.070	-0.025	+0.002	-0.023	+11.594	0.148
Al1–H	+0.077	+0.205	-0.112	-0.109	+0.427	+0.031	-0.104	+0.078	-0.026	+1.340	0.399
Al2–H	+0.077	+0.201	-0.110	-0.107	+0.419	+0.029	-0.103	+0.076	-0.026	+1.342	0.407
Al1–N	+0.074	+0.354	-0.117	-0.108	+0.578	+0.082	-0.115	+0.102	-0.013	+1.132	0.302
Al2–N	+0.073	+0.347	-0.114	-0.105	+0.566	+0.079	-0.113	+0.100	-0.013	+1.130	0.314
Al1–P	+0.047	+0.070	-0.046	-0.044	+0.160	+0.043	-0.049	+0.033	-0.016	+1.469	0.279
Al2–P	+0.049	+0.077	-0.049	-0.047	+0.173	+0.036	-0.052	+0.036	-0.016	+1.463	0.296

X-Ray Crystallography

Dialumene 1

Crystal Data and Experimental

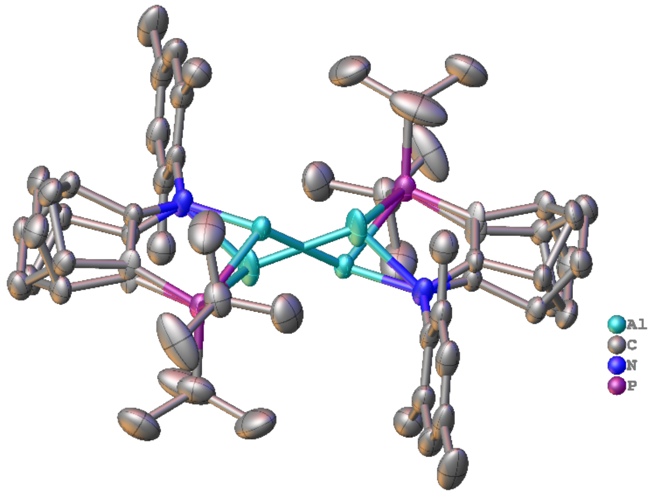

Experimental. Single dark purple block-shaped crystals of **1** were obtained by recrystallisation from a concentrated toluene solution at -30 °C. A suitable crystal 0.17×0.11×0.05 mm^3 was selected and mounted on a suitable support on a SuperNova, Dual, Cu at home/near, Atlas diffractometer. The crystal was kept at a steady *T* = 120.01(10) K during data collection. The structure was solved with the **ShelXT** (Sheldrick, 2015) structure solution program using the intrinsic phasing methods solution method and by using **Olex2** (Dolomanov et al., 2009) as the graphical interface. The model was refined with version 2018/3 of **ShelXL** (Sheldrick, 2015) using full matrix least squares on **F^2** minimisation.

Crystal Data. $C_{55}H_{81}Al_2N_2P_2$, M_r = 886.11, monoclinic, $P2_1/n$ (No. 14), a = 13.1729(3) Å, b = 14.1285(3) Å, c = 14.0626(2) Å, *b* = 92.042(2)°, *a* = *g* = 90°, *V* = 2615.58(9) Å^3, *T* = 120.01(10) K, *Z* = 2, *Z'* = 0.5, *m*(CuK$_a$) = 1.341, 11560 reflections measured, 11560 unique (R_{int} = .) which were used in all calculations. The final wR_2 was 0.1726 (all data) and R_1 was 0.0614 (I > 2(I)).

Compound	**1**
Formula	$C_{55}H_{81}Al_2N_2P_2$
$D_{calc.}$/ g cm^{-3}	1.125
m/mm^{-1}	1.341
Formula Weight	886.11
Colour	dark purple
Shape	block
Size/mm^3	0.17×0.11×0.05
T/K	120.01(10)
Crystal System	monoclinic
Space Group	$P2_1/n$
a/Å	13.1729(3)
b/Å	14.1285(3)
c/Å	14.0626(2)
a/°	90
b/°	92.042(2)
g/°	90
V/Å^3	2615.58(9)
Z	2
Z'	0.5
Wavelength/Å	1.54184
Radiation type	CuK$_a$
Q_{min}/°	4.438
Q_{max}/°	76.511
Measured Refl.	11560
Independent Refl.	11560
Reflections with I > 2(I)	9408
R_{int}	19.98
Parameters	373
Restraints	48
Largest Peak	0.426
Deepest Hole	-0.342
GooF	1.035
wR_2 (all data)	0.1726
wR_2	0.1660
R_1 (all data)	0.0730
R_1	0.0614

Structure Quality Indicators

Reflections:	d min (Cu) CIF 0.79	I/σ CIF 9.5	Rint CIF 19.98%	complete CIF 100%
Refinement:	Shift CIF 0.001	Max Peak CIF 0.4	Min Peak CIF -0.3	GooF CIF 1.035

A dark purple block-shaped crystal with dimensions 0.17×0.11×0.05 mm^3 was mounted on a suitable support. Data were collected using a SuperNova, Dual, Cu at home/near, Atlas diffractometer operating at T = 120.01(10) K.

Data were measured using *w* scans using CuK$_a$ radiation. The total number of runs and images was based on the strategy calculation from the program **CrysAlisPro** (Rigaku, V1.171.41.99a, 2021). The maximum resolution that was achieved was Q = 76.511° (0.83 Å).

The diffraction pattern was indexed The total number of runs and images was based on the strategy calculation from the program **CrysAlisPro** (Rigaku, V1.171.41.99a, 2021) and the unit cell was refined using **CrysAlisPro** (Rigaku, V1.171.41.99a, 2021) on 18089 reflections, 156% of the observed reflections.

Data reduction, scaling and absorption corrections were performed using **CrysAlisPro** (Rigaku, V1.171.41.99a, 2021). The final completeness is 100.00 % out to 76.511° in Q. A multi-scan absorption correction was performed using CrysAlisPro 1.171.41.99a (Rigaku Oxford Diffraction, 2021) using spherical harmonics,implemented in SCALE3 ABSPACK scaling algorithm.. The absorption coefficient *m* of this material is 1.341 mm^{-1} at this wavelength (l = 1.542Å) and the minimum and maximum transmissions are 0.632 and 1.000.

The structure was solved and the space group $P2_1/n$ (# 14) determined by the **ShelXT** (Sheldrick, 2015) structure solution program using Intrinsic Phasing methods and refined by full matrix least squares on **F**^{2} using version 2018/3 of **ShelXL** (Sheldrick, 2015). All non-hydrogen atoms were refined anisotropically. Hydrogen atom positions were calculated geometrically and refined using the riding model. Hydrogen atom positions were calculated geometrically and refined using the riding model.

_refine_special_details: The structure was modelled as a non-merohedral twin with twin law [-1 0 0 / 0 -1 0 / 0 0 1] (BASF = 0.3212(12)). A disordered toluene solvent molecule using the FragmentDB function of Olex2 and fitting the idealised molecules onto peaks observed in a difference map. RIGU and SADI restraints were used to control the toluene.

_exptl_absorpt_process_details: CrysAlisPro 1.171.41.99a (Rigaku Oxford Diffraction, 2021) using spherical harmonics,implemented in SCALE3 ABSPACK scaling algorithm.

_twin_special_details: Component 2 rotated by 179.9003° around [-0.03 0.00 1.00] (reciprocal) or [0.00 0.00 1.00] (direct)

The value of Z' is 0.5. This means that only half of the formula unit is present in the asymmetric unit, with the other half consisting of symmetry equivalent atoms.

Dialuminacyclobutane 4

Crystal Data and Experimental

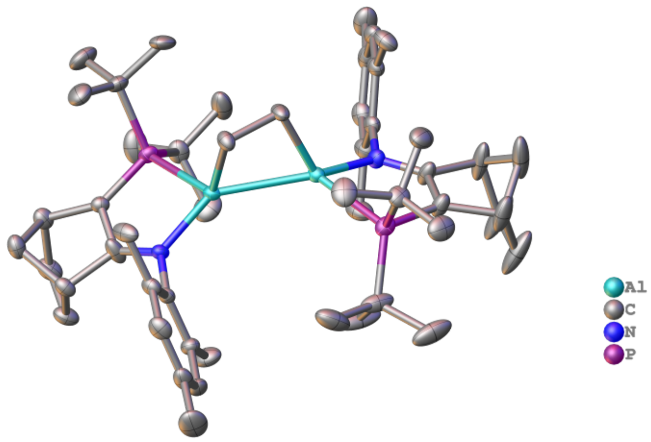

Experimental. Single colourless slab-shaped crystals of **4** were obtained by recrystallisation from a concentrated pentane solution at room temperature. A suitable crystal 0.60×0.30×0.20 mm^3 was selected and mounted on a suitable support on an Bruker APEX-II CCD diffractometer. The crystal was kept at a steady *T* = 100(2) K during data collection. The structure was solved with the ShelXT 2018/2 (Sheldrick, 2018) structure solution program using the intrinsic phasing methods solution method and by using **Olex2** (Dolomanov et al., 2009) as the graphical interface. The model was refined with version 2018/3 of **ShelXL** (Sheldrick, 2015) using full matrix least squares on **F**^{2} minimisation.

Crystal Data. $C_{50}H_{78}Al_2N_2P_2$, M_r = 823.04, monoclinic, $P2_1/c$ (No. 14), a = 8.5372(9) Å, b = 18.1329(18) Å, c = 31.742(3) Å, b = 94.142(3)°, $a = g = 90°$, V = 4900.9(9) Å^3, T = 100(2) K, Z = 4, Z' = 1, m(MoK$_a$) = 0.158, 165160 reflections measured, 14968 unique (R_{int} = 0.0374) which were used in all calculations. The final wR_2 was 0.1163 (all data) and R_1 was 0.0448 (I > 2(I)).

Compound	**4**
Formula	$C_{50}H_{78}Al_2N_2P_2$
$D_{calc.}$/ g cm^{-3}	1.115
m/mm^{-1}	0.158
Formula Weight	823.04
Colour	colourless
Shape	slab
Size/mm^3	0.60×0.30×0.20
T/K	100(2)
Crystal System	monoclinic
Space Group	$P2_1/c$
a/Å	8.5372(9)
b/Å	18.1329(18)
c/Å	31.742(3)
a/°	90
b/°	94.142(3)
g/°	90
V/Å^3	4900.9(9)
Z	4
Z'	1
Wavelength/Å	0.71073
Radiation type	MoK$_a$
Q_{min}/°	2.233
Q_{max}/°	30.552
Measured Refl.	165160
Independent Refl.	14968
Reflections with I > 2(I)	13644
R_{int}	0.0374
Parameters	682
Restraints	251
Largest Peak	0.823
Deepest Hole	-0.758
GooF	1.096
wR_2 (all data)	0.1163
wR_2	0.1138
R_1 (all data)	0.0489
R_1	0.0448

Structure Quality Indicators

Reflections:	d min (Mo) CIF 0.70	I/σ CIF 50.4	Rint CIF 3.74%	complete CIF 100%
Refinement:	Shift CIF 0.001	Max Peak CIF 0.8	Min Peak CIF -0.8	GooF CIF 1.096

A colourless slab-shaped crystal with dimensions 0.60×0.30×0.20 mm^3 was mounted on a suitable support. Data were collected using an Bruker APEX-II CCD diffractometer operating at T = 100(2) K.

Data were measured using *f* and *w* scans using MoK$_a$ radiation. The maximum resolution that was achieved was Q = 30.552° (0.83 Å).

The diffraction pattern was indexed and the unit cell was refined using **SAINT** (Bruker, V8.40A, after 2013) on 9905 reflections, 6% of the observed reflections.

Data reduction, scaling and absorption corrections were performed using **SAINT** (Bruker, V8.40A, after 2013). The final completeness is 99.90 % out to 30.552° in Q. A multi-scan absorption correction was performed using **SADABS**-2016/2 (Bruker,2016/2) was used for absorption correction. wR_2(int) was 0.1442 before and 0.0576 after correction. The Ratio of minimum to maximum transmission is 0.9672. The *l*/2 correction factor is Not present.. The absorption coefficient *m* of this material is 0.158 mm^{-1} at this wavelength (*l* = 0.711Å) and the minimum and maximum transmissions are 0.722 and 0.746.

The structure was solved and the space group $P2_1/c$ (# 14) determined by the ShelXT 2018/2 (Sheldrick, 2018) structure solution program using Intrinsic Phasing methods and refined by full matrix least squares on $\mathbf{F}^2$ using version 2018/3 of **ShelXL** (Sheldrick, 2015). All non-hydrogen atoms were refined anisotropically. Hydrogen atom positions were calculated geometrically and refined using the riding model. Hydrogen atom positions were calculated geometrically and refined using the riding model.

_refine_special_details: Some poorly fitting reflections were omitted during refinement, as listed in the embedded res file. Disordered components were controlled with SADI, RIGU, DELU and SIMU restraints.

_exptl_absorpt_process_details: **SADABS**-2016/2 (Bruker,2016/2) was used for absorption correction. wR_2(int) was 0.1442 before and 0.0576 after correction. The Ratio of minimum to maximum transmission is 0.9672. The *l*/2 correction factor is Not present.

There is a single molecule in the asymmetric unit, which is represented by the reported sum formula. In other words: Z is 4 and Z' is 1.

Dialuminacyclobutene 5

Crystal Data and Experimental

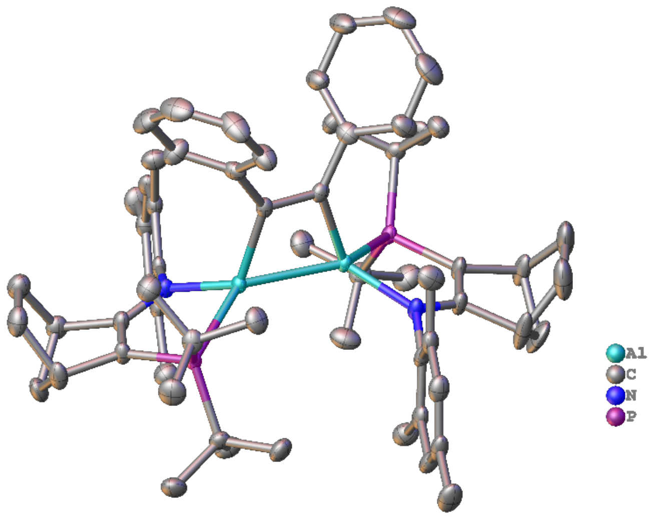

Experimental. Single yellow block-shaped crystals of **5** were obtained by recrystallisation from pentane at -30 °C. A suitable crystal 0.34×0.22×0.18 mm^3 was selected and mounted on a suitable support on an Xcalibur, Eos diffractometer. The crystal was kept at a steady *T* = 120.01(10) K during data collection. The structure was solved with the **ShelXT** (Sheldrick, 2015) structure solution program using the Intrinsic Phasing methods solution method and by using **Olex2** (Dolomanov et al., 2009) as the graphical interface. The model was refined with version 2018/3 of ShelXL 2018/3 (Sheldrick, 2015) using full matrix least squares on **F**^{2} minimisation.

Crystal Data. $C_{74.73}H_{102.54}Al_2N_2P_2$, M_r = 1144.78, monoclinic, $P2_1/c$ (No. 14), a = 13.8267(3) Å, b = 14.4472(4) Å, c = 33.3523(9) Å, *b* = 90.127(2)°, *a* = *g* = 90°, *V* = 6662.3(3) Å^3, *T* = 120.01(10) K, *Z* = 4, *Z'* = 1, *m*(MoK$_a$) = 0.135, 140898 reflections measured, 15027 unique (R_{int} = 0.0783) which were used in all calculations. The final wR_2 was 0.1504 (all data) and R_1 was 0.0638 (I > 2(I)).

Compound	**5**
Formula	$C_{74.73}H_{102.54}Al_2N_2P_2$
$D_{calc.}$/ g cm^{-3}	1.141
m/mm^{-1}	0.135
Formula Weight	1144.78
Colour	colourless
Shape	block
Size/mm^3	0.34×0.22×0.18
T/K	120.01(10)
Crystal System	monoclinic
Space Group	$P2_1/c$
a/Å	13.8267(3)
b/Å	14.4472(4)
c/Å	33.3523(9)
a/°	90
b/°	90.127(2)
g/°	90
V/Å^3	6662.3(3)
Z	4
Z'	1
Wavelength/Å	0.71073
Radiation type	MoK$_a$
Q_{min}/°	3.185
Q_{max}/°	28.240
Measured Refl.	140898
Independent Refl.	15027
Reflections with I > 2(I)	13201
R_{int}	0.0783
Parameters	800
Restraints	253
Largest Peak	0.608
Deepest Hole	-0.407
GooF	1.079
wR_2 (all data)	0.1504
wR_2	0.1443
R_1 (all data)	0.0756
R_1	0.0638

Structure Quality Indicators

Reflections:	d min (Mo) CIF 0.75	I/σ CIF 19.8	Rint CIF 7.76%	complete CIF 100%
Refinement:	Shift CIF 0.001	Max Peak CIF 0.6	Min Peak CIF -0.4	GooF CIF 1.079

A yellow block-shaped crystal with dimensions 0.34×0.22×0.18 mm^3 was mounted on a suitable support. Data were collected using an Xcalibur, Eos diffractometer operating at T = 120.01(10) K.

Data were measured using *w* scans using MoK_a radiation. The total number of runs and images was based on the strategy calculation from the program **CrysAlisPro** (Rigaku, V1.171.41.99a, 2021). The maximum resolution that was achieved was Q = 28.240° (0.83 Å).

The diffraction pattern was indexed The total number of runs and images was based on the strategy calculation from the program **CrysAlisPro** (Rigaku, V1.171.41.99a, 2021) and the unit cell was refined using **CrysAlisPro** (Rigaku, V1.171.41.99a, 2021) on 33888 reflections, 24% of the observed reflections.

Data reduction, scaling and absorption corrections were performed using **CrysAlisPro** (Rigaku, V1.171.41.99a, 2021). The final completeness is 99.80 % out to 28.240° in Q. A multi-scan absorption correction was performed using CrysAlisPro 1.171.41.99a (Rigaku Oxford Diffraction, 2021) using spherical harmonics,implemented in SCALE3 ABSPACK scaling algorithm.. The absorption coefficient m of this material is 0.135 mm^{-1} at this wavelength (l = 0.711Å) and the minimum and maximum transmissions are 0.946 and 1.000.

The structure was solved and the space group $P2_1/c$ (# 14) determined by the **ShelXT** (Sheldrick, 2015) structure solution program using Intrinsic Phasing methods and refined by full matrix least squares on **F**^{2} using version 2018/3 of ShelXL 2018/3 (Sheldrick, 2015). All non-hydrogen atoms were refined anisotropically. Hydrogen atom positions were calculated geometrically and refined using the riding model. Hydrogen atom positions were calculated geometrically and refined using the riding model.

_exptl_absorpt_process_details: CrysAlisPro 1.171.41.99a (Rigaku Oxford Diffraction, 2021) using spherical harmonics,implemented in SCALE3 ABSPACK scaling algorithm.

There is a single molecule in the asymmetric unit, which is represented by the reported sum formula. In other words: Z is 4 and Z' is 1.

_refine_special_details: The structure was modelled as a pseudomerohedral twin with (BASF = 0.3212(12)). Two regions of disordered solvent were identified and modelled. One consists of two toluene molecules alternating position over a symmetry element, which were refined using an isotropic model. The second was modelled as a disordered mix of pentane (64%) and toluene (36%). In all cases the disordered molecules were modelled using the FragmentDB function of Olex2 and fitting the idealised molecules onto peaks observed in a difference map.

Aluminacyclopropene 6

Crystal Data and Experimental

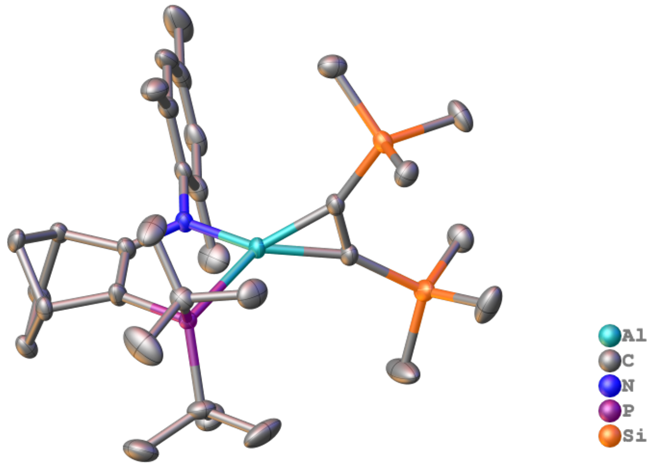

Compound	**6**
Formula	$C_{32}H_{55}AlNPSi_2$
$D_{calc.}$/ g cm^{-3}	1.066
m/mm^{-1}	0.190
Formula Weight	567.90
Colour	colourless
Shape	block
Size/mm^3	0.73×0.47×0.20
T/K	120.00
Crystal System	monoclinic
Space Group	*C*2/*c*
a/Å	36.6020(5)
b/Å	12.01810(10)
c/Å	36.9872(6)
a/°	90
b/°	119.535(2)
g/°	90
V/Å^3	14155.9(4)
Z	16
Z'	2
Wavelength/Å	0.71073
Radiation type	MoK$_a$
Q_{min}/°	3.273
Q_{max}/°	28.193
Measured Refl.	151007
Independent Refl.	16443
Reflections with I > 2(I)	14715
R_{int}	0.0406
Parameters	815
Restraints	136
Largest Peak	0.612
Deepest Hole	-0.326
GooF	1.076
wR_2 (all data)	0.1135
wR_2	0.1097
R_1 (all data)	0.0558
R_1	0.0482

Experimental. Single orange block-shaped crystals of **6** were obtained by recrystallisation from a concentrated toluene solution at -30 °C. A suitable crystal 0.73×0.47×0.20 mm^3 was selected and mounted on a suitable support on an Rigaku Oxford Diffraction XCalibur diffractometer. The crystal was kept at a steady *T* = 120.00 K during data collection. The structure was solved with the **ShelXT** (Sheldrick, 2015) structure solution program using the intrinsic phasing methods solution method and by using **Olex2** (Dolomanov et al., 2009) as the graphical interface. The model was refined with version 2018/3 of ShelXL 2018/3 (Sheldrick, 2015) using full matrix least squares on **F**^{2} minimisation.

Crystal Data. $C_{32}H_{55}AlNPSi_2$, M_r = 567.90, monoclinic, *C*2/*c* (No. 15), a = 36.6020(5) Å, b = 12.01810(10) Å, c = 36.9872(6) Å, *b* = 119.535(2)°, *a* = *g* = 90°, *V* = 14155.9(4) Å^3, *T* = 120.00 K, *Z* = 16, *Z'* = 2, *m*(MoK$_a$) = 0.190, 151007 reflections measured, 16443 unique (R_{int} = 0.0406) which were used in all calculations. The final wR_2 was 0.1135 (all data) and R_1 was 0.0482 (I > 2(I)).

Structure Quality Indicators

Reflections:	d min (Mo) CIF 0.83	I/σ CIF 37.7	Rint CIF 4.06%	complete 100%
Refinement:	Shift CIF 0.002	Max Peak CIF 0.6	Min Peak CIF -0.3	GooF CIF 1.076

An orange block-shaped crystal with dimensions 0.73×0.47×0.20 mm^3 was mounted on a suitable support. Data were collected using an Rigaku Oxford Diffraction XCalibur diffractometer operating at T = 120.00 K.

Data were measured using *w* scans using MoK_a radiation. The total number of runs and images was based on the strategy calculation from the program **CrysAlisPro** (Rigaku, V1.171.41.99a, 2021). The maximum resolution that was achieved was Q = 28.193° (0.83 Å).

The diffraction pattern was indexed The total number of runs and images was based on the strategy calculation from the program **CrysAlisPro** (Rigaku, V1.171.41.99a, 2021) and the unit cell was refined using **CrysAlisPro** (Rigaku, V1.171.41.99a, 2021) on 60312 reflections, 40% of the observed reflections.

Data reduction, scaling and absorption corrections were performed using **CrysAlisPro** (Rigaku, V1.171.41.99a, 2021). The final completeness is 99.80 % out to 28.193° in Q. A multi-scan absorption correction was performed using CrysAlisPro 1.171.41.99a (Rigaku Oxford Diffraction, 2021) using spherical harmonics,implemented in SCALE3 ABSPACK scaling algorithm.. The absorption coefficient m of this material is 0.190 mm^{-1} at this wavelength (l = 0.711Å) and the minimum and maximum transmissions are 0.841 and 1.000.

The structure was solved and the space group *C*2/*c* (# 15) determined by the **ShelXT** (Sheldrick, 2015) structure solution program using Intrinsic Phasing methods and refined by full matrix least squares on $\mathbf{F}^2$ using version 2018/3 of ShelXL 2018/3 (Sheldrick, 2015). All non-hydrogen atoms were refined anisotropically. Hydrogen atom positions were calculated geometrically and refined using the riding model. Hydrogen atom positions were calculated geometrically and refined using the riding model.

_exptl_absorpt_process_details: CrysAlisPro 1.171.41.99a (Rigaku Oxford Diffraction, 2021) using spherical harmonics,implemented in SCALE3 ABSPACK scaling algorithm.

_refine_special_details: Twin law [-1 0 0 / 0 -1 0 / 1 0 1] was used to account for the metric symmetry mimicking a hexagonal setting. The refined twin scale factor (0.0234(2)) is small but makes a visible difference to the displacement ellipsoids and the refinement residuals. Positional disorder in the model was identified from peaks in a difference Fourier map and refined with appropriate geometric and displacement ellipsoid restraints.

Citations

O.V. Dolomanov and L.J. Bourhis and R.J. Gildea and J.A.K. Howard and H. Puschmann, Olex2: A complete structure solution, refinement and analysis program, *J. Appl. Cryst.*, (2009), **42**, 339-341.

Sheldrick, G.M., Crystal structure refinement with ShelXL, *Acta Cryst.*, (2015), **C27**, 3-8.

Software for the Integration of CCD Detector System Bruker Analytical X-ray Systems, Bruker axs, Madison, WI (after 2013).

NMR spectra of reported compounds

Dialumene 1

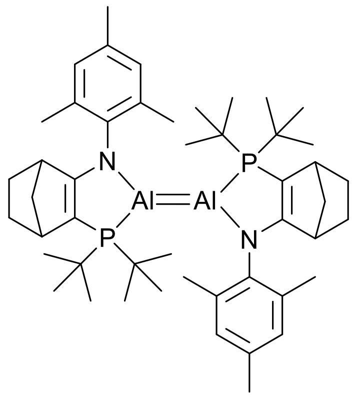

^{1}H

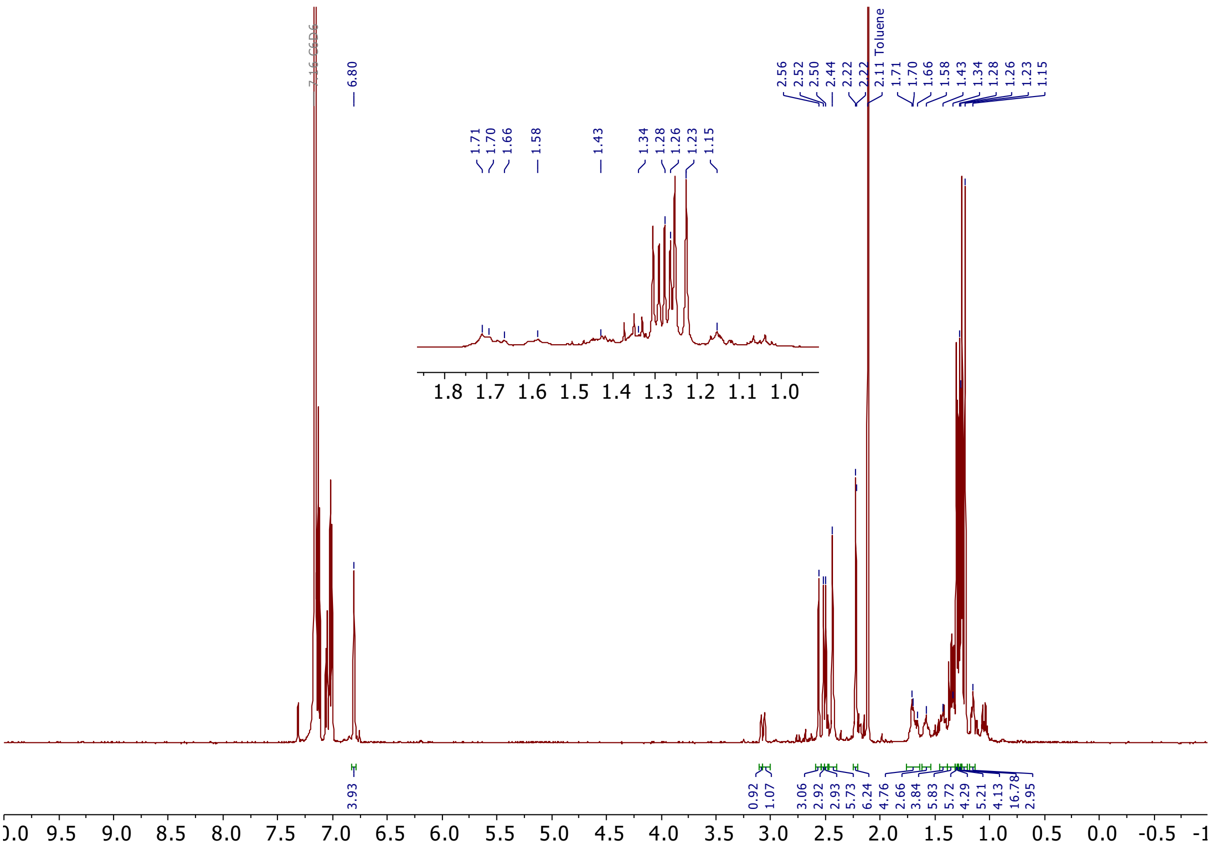

^{13}C

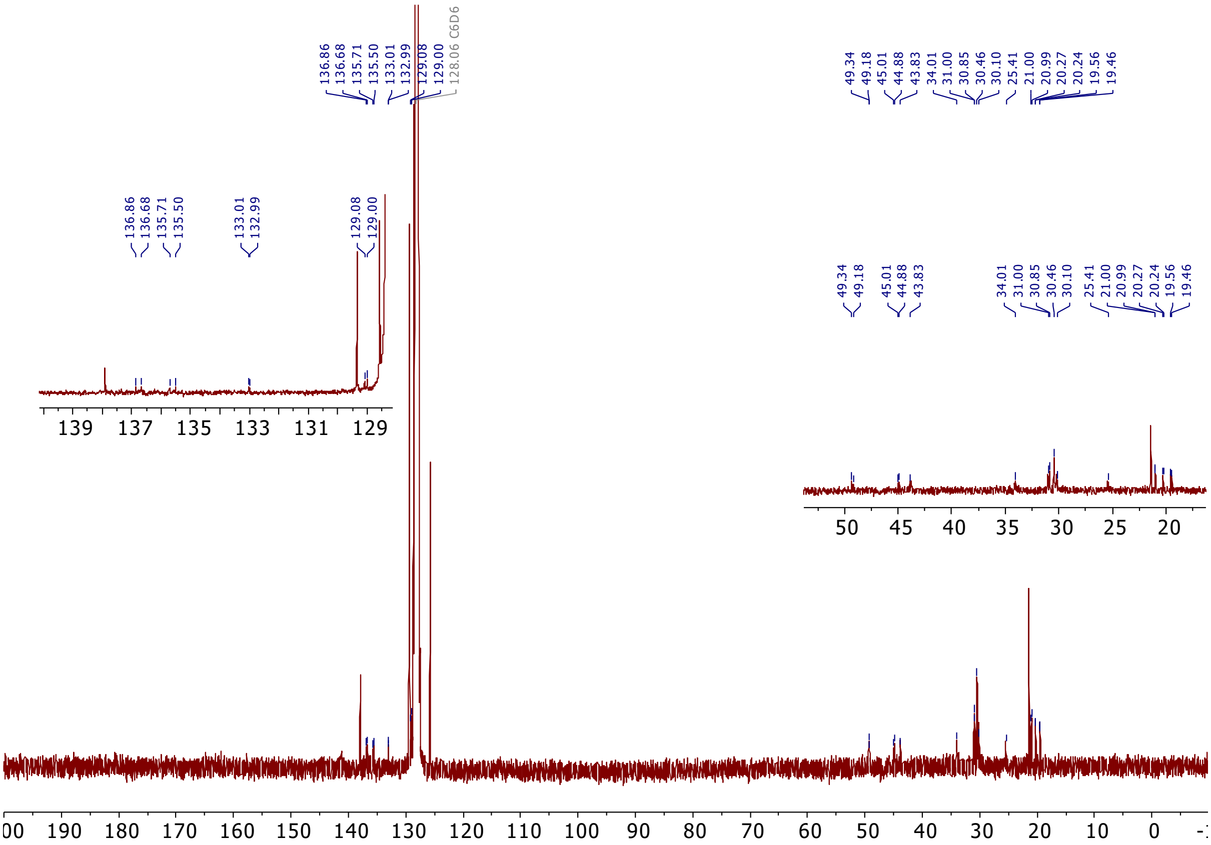

HSQC – selected section for assignment of ^{31}C resonances

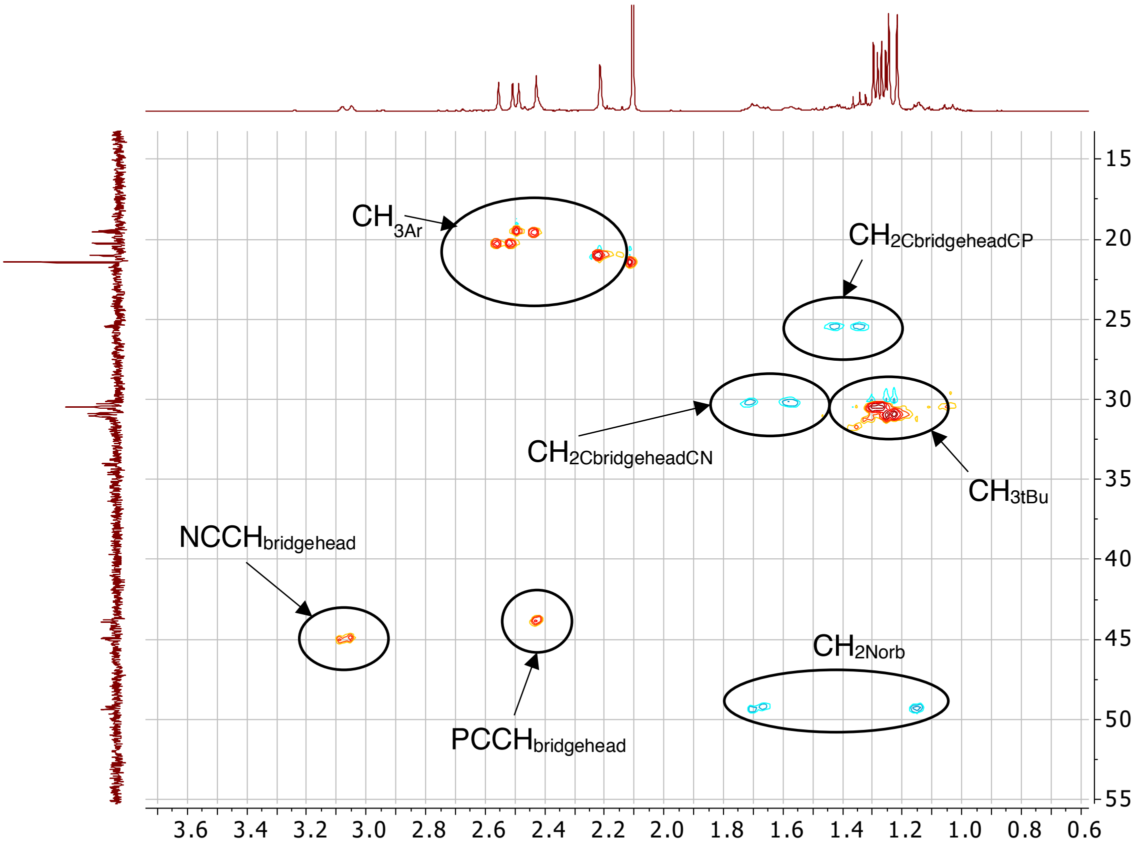

$^{31}P\{^{1}H\}$

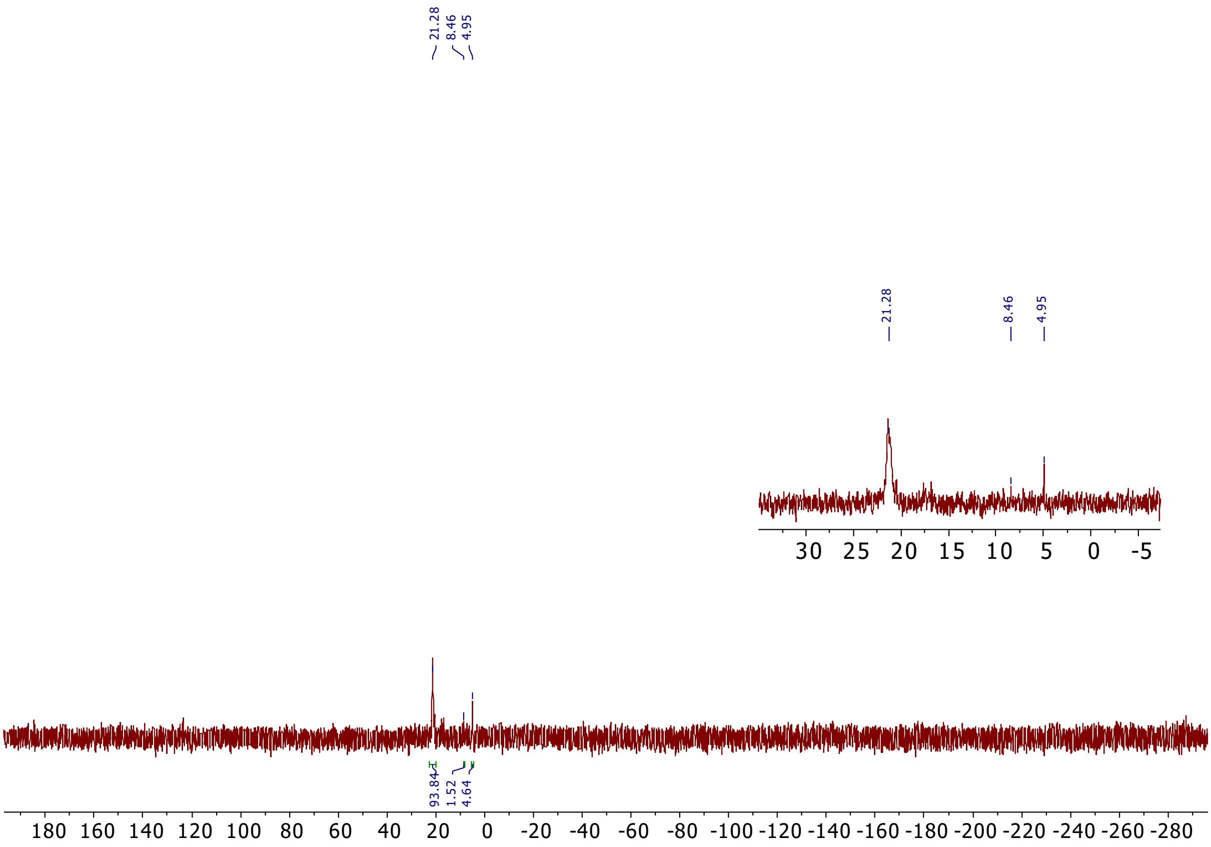
21.28
8.46
4.95
93.84
1.52
4.64
180 160 140 120 100 80 60 40 20 0 -20 -40 -60 -80 -100 -120 -140 -160 -180 -200 -220 -240 -260 -280
30 25 20 15 10 5 0 -5

Compound 4

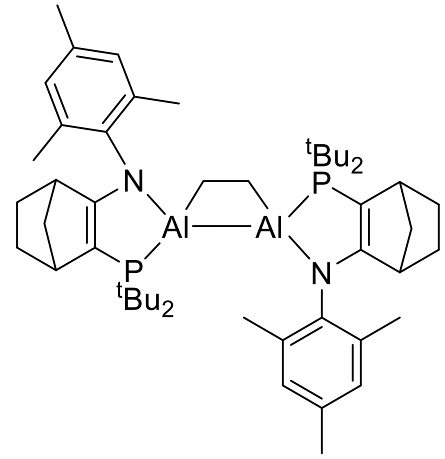

1H

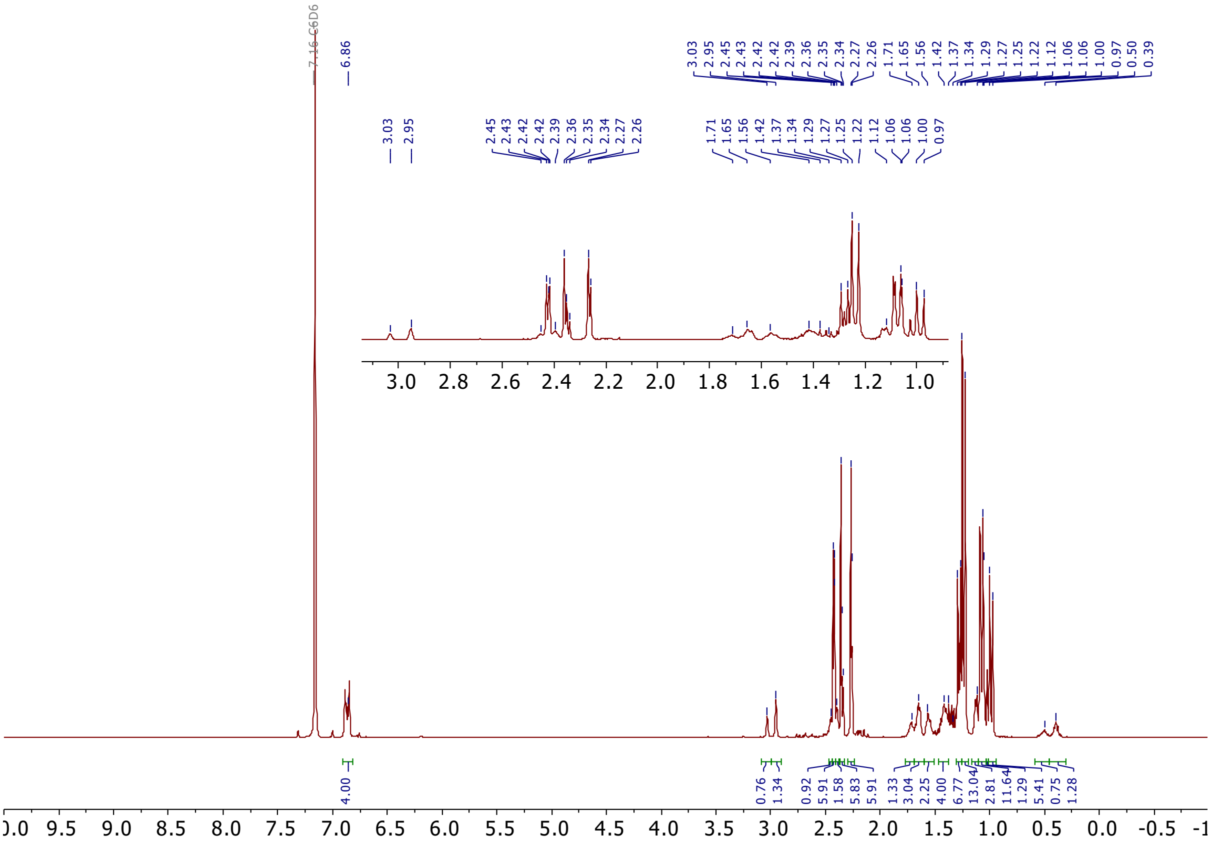

^{13}C

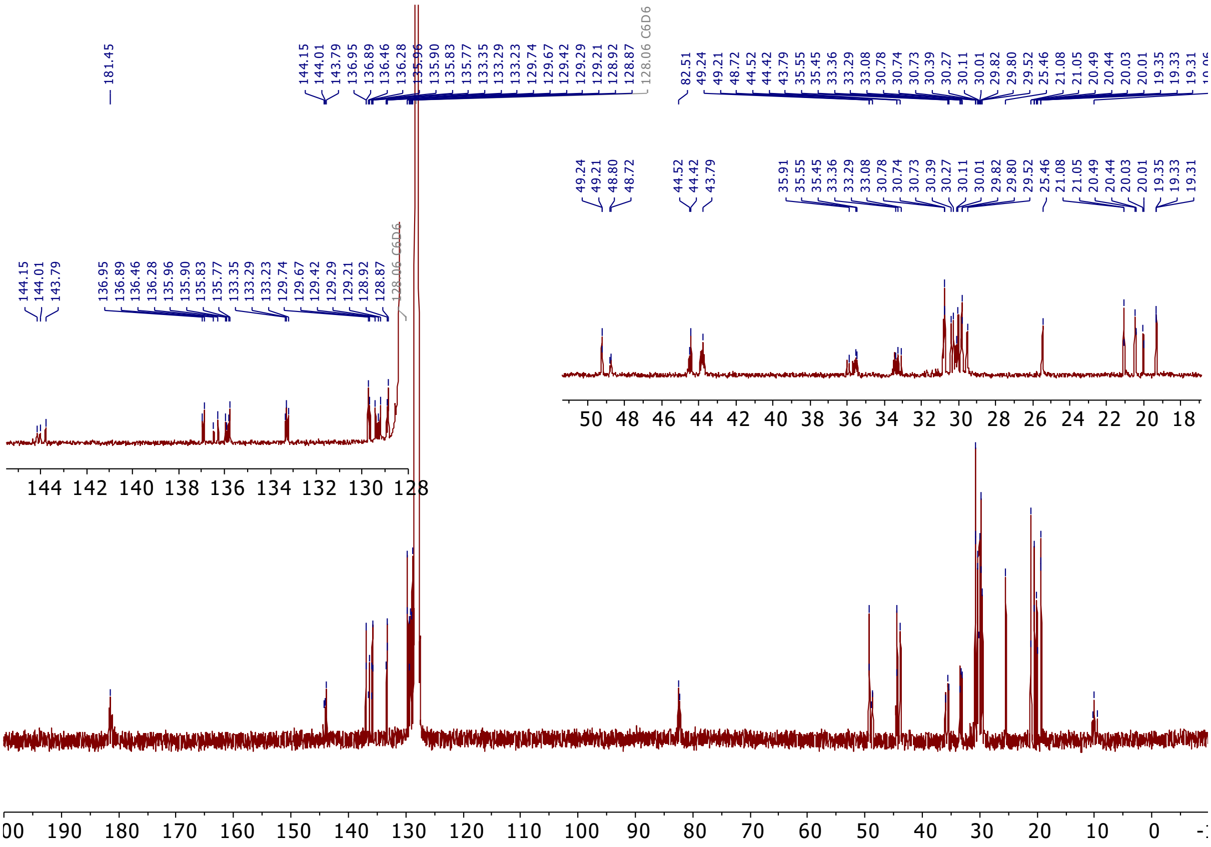

181.45
128.06 C6D6
82.51
144 142 140 138 136 134 132 130 128
50 48 46 44 42 40 38 36 34 32 30 28 26 24 22 20 18
190 180 170 160 150 140 130 120 110 100 90 80 70 60 50 40 30 20 10 0

HSQC

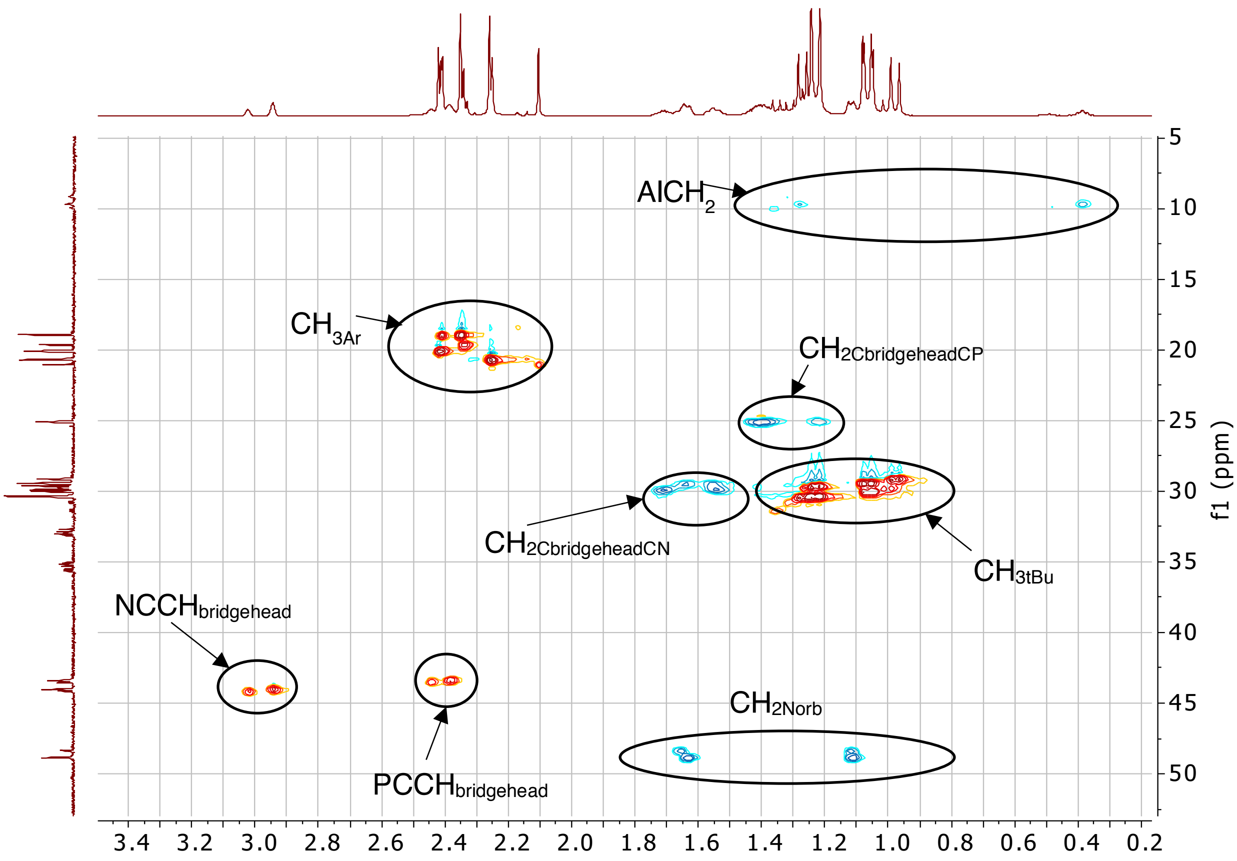

AlCH2
CH3Ar
CH2CbridgeheadCP
CH2CbridgeheadCN
CH3tBu
NCCHbridgehead
PCCHbridgehead
CH2Norb
f1 (ppm)
3.4 3.2 3.0 2.8 2.6 2.4 2.2 2.0 1.8 1.6 1.4 1.2 1.0 0.8 0.6 0.4 0.2
5 10 15 20 25 30 35 40 45 50

$^{31}P\{^1H\}$

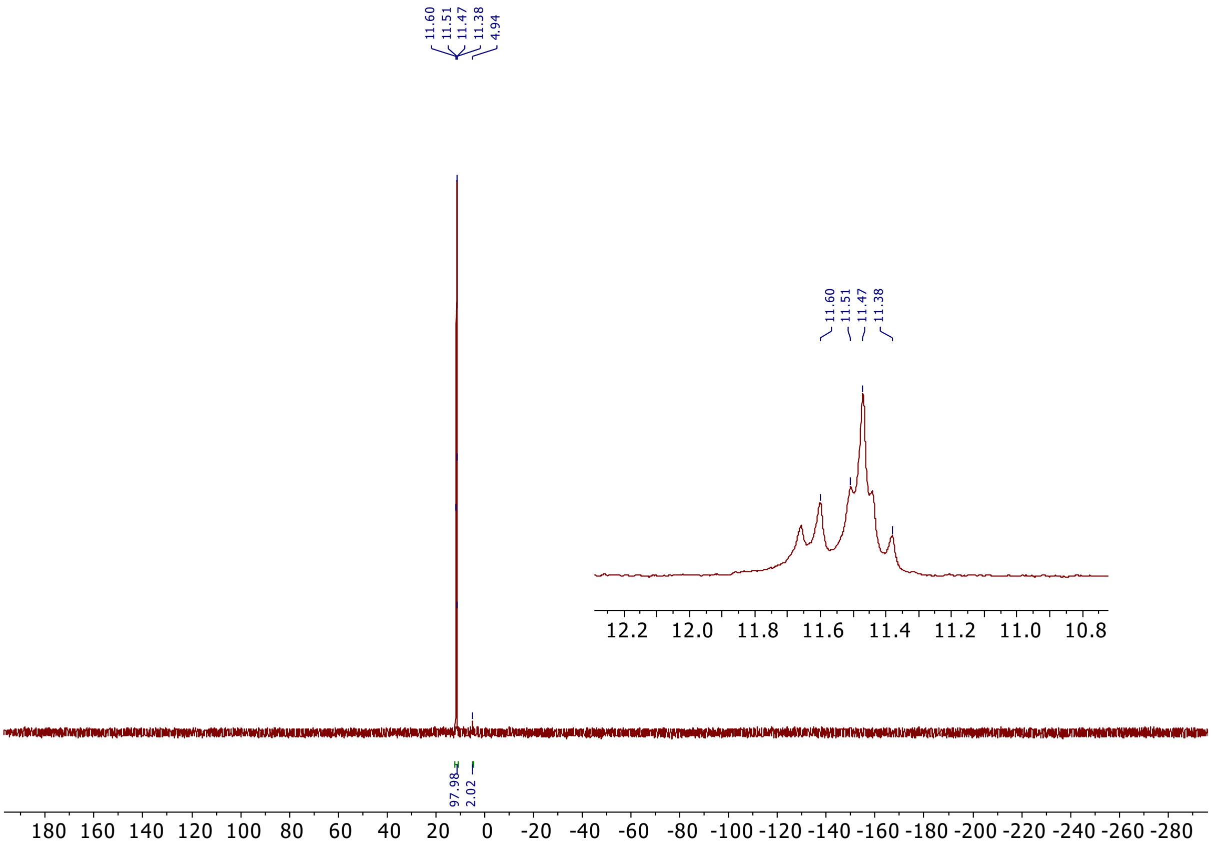

Resonance at 4.94 ppm likely due to reaction with water

Compound 5

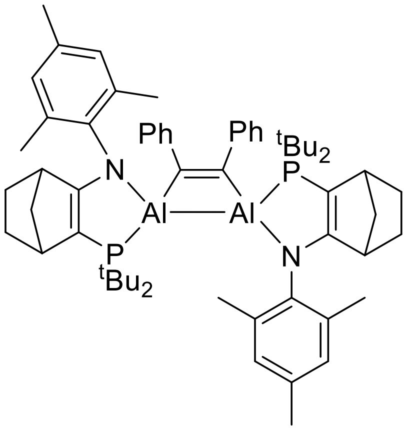

^{1}H

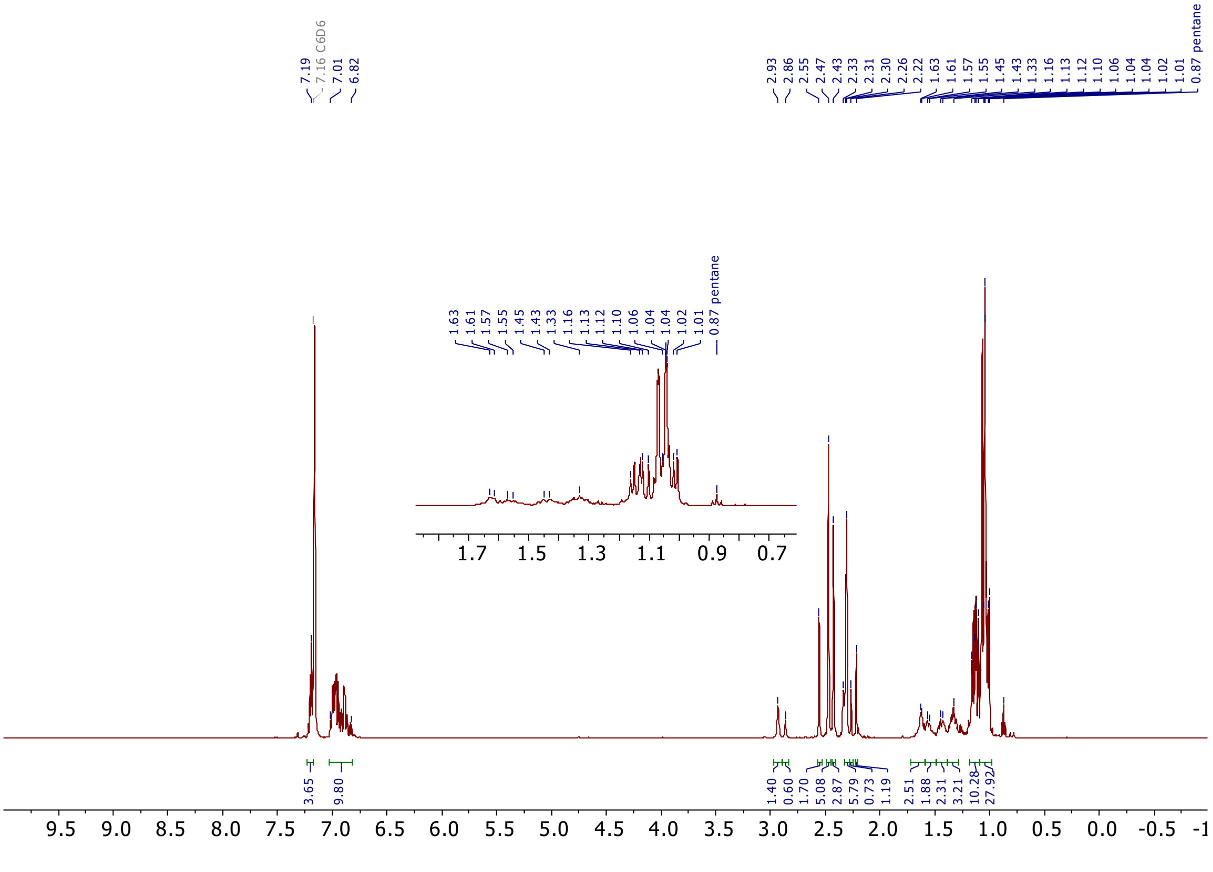

^{13}C

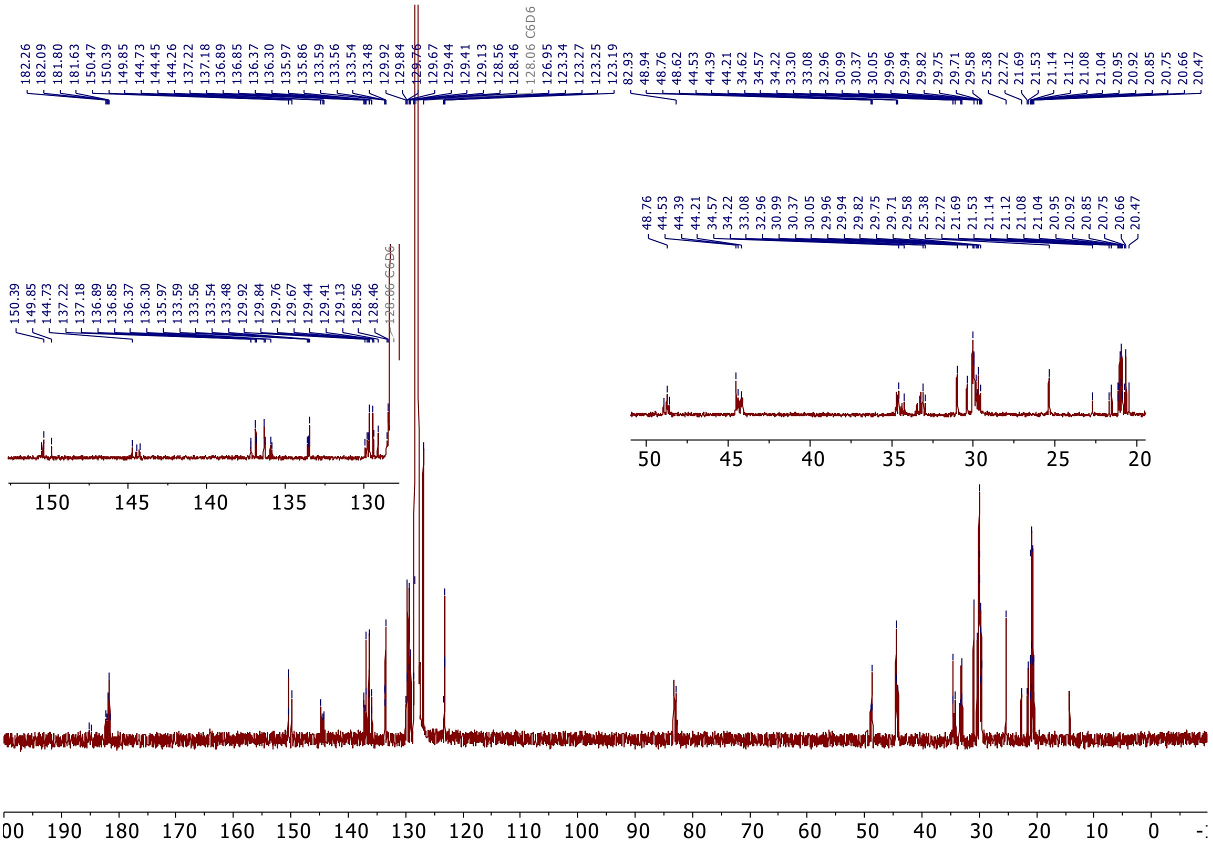
182.26
182.09
181.80
181.63
150.47
150.39
149.85
144.73
144.45
144.26
137.22
137.18
136.89
136.85
136.37
136.30
135.97
135.86
133.59
133.56
133.54
133.48
129.92
129.84
129.76
129.67
129.44
129.41
129.13
128.56
128.46
128.06 C6D6
126.95
123.34
123.27
123.25
123.19
82.93
48.94
48.76
48.62
44.53
44.39
44.21
34.62
34.57
34.22
33.30
33.08
32.96
30.99
30.37
30.05
29.96
29.94
29.82
29.75
29.71
29.58
25.38
22.72
21.69
21.53
21.14
21.12
21.08
21.04
20.95
20.92
20.85
20.75
20.66
20.47

HSQC

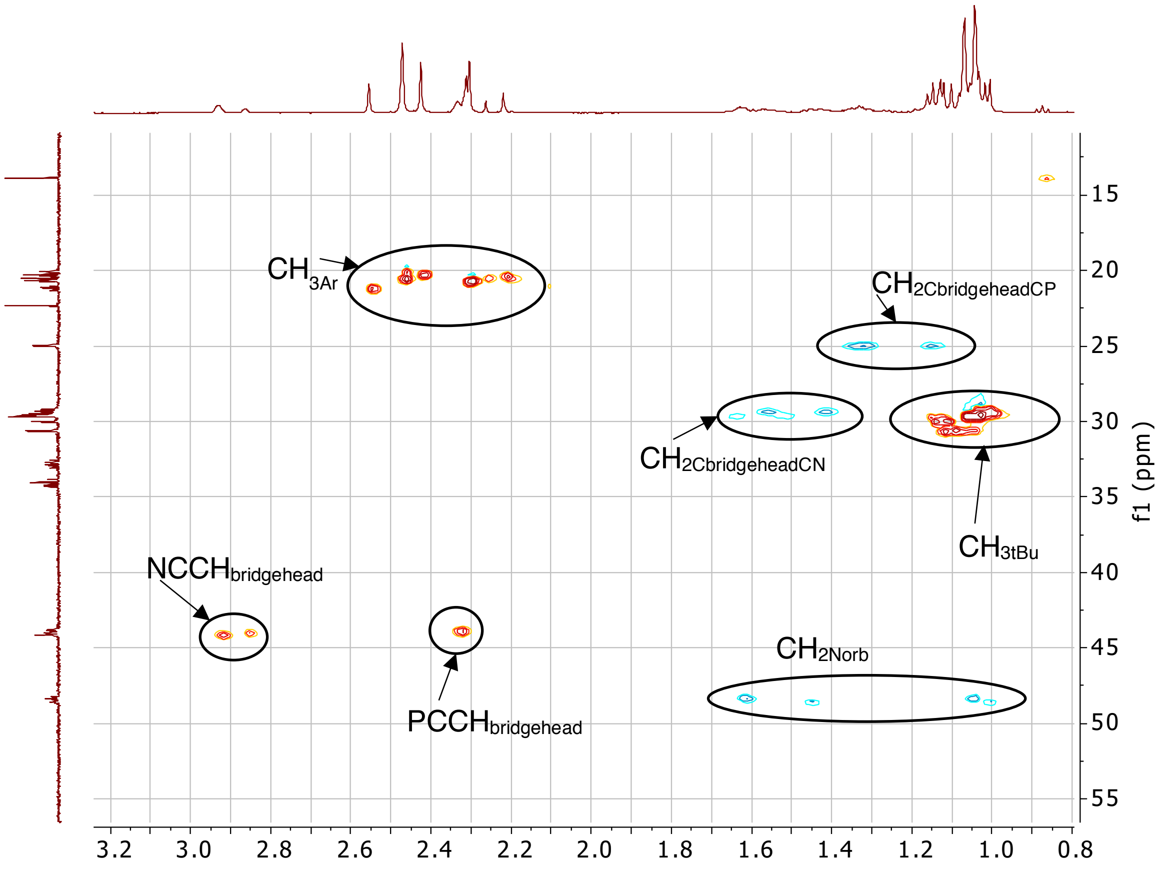
CH3Ar
CH2CbridgeheadCP
CH2CbridgeheadCN
CH3tBu
NCCHbridgehead
PCCHbridgehead
CH2Norb
f1 (ppm)

$^{31}P\{^{1}H\}$

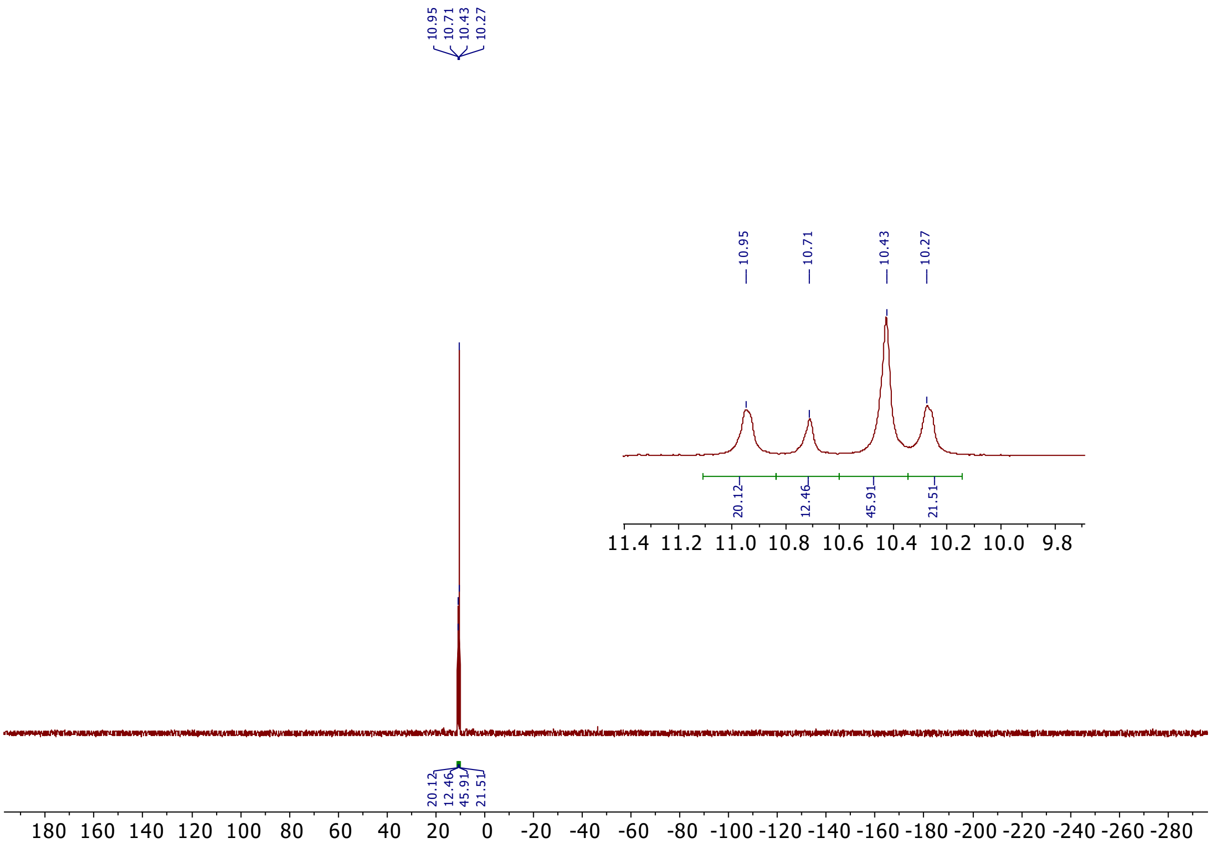
10.95
10.71
10.43
10.27
20.12
12.46
45.91
21.51
11.4 11.2 11.0 10.8 10.6 10.4 10.2 10.0 9.8
180 160 140 120 100 80 60 40 20 0 -20 -40 -60 -80 -100 -120 -140 -160 -180 -200 -220 -240 -260 -280

Compound 6

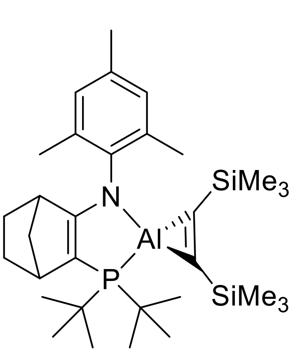

^{1}H

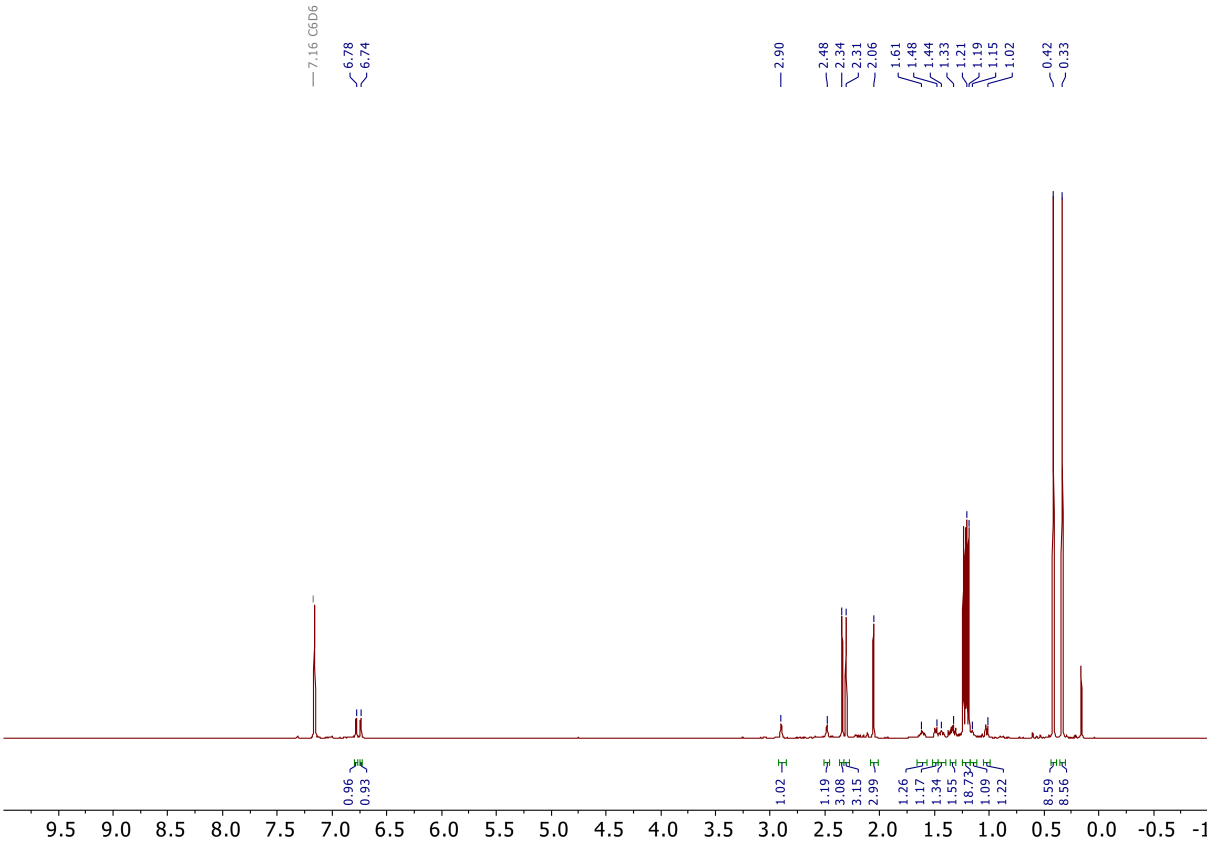

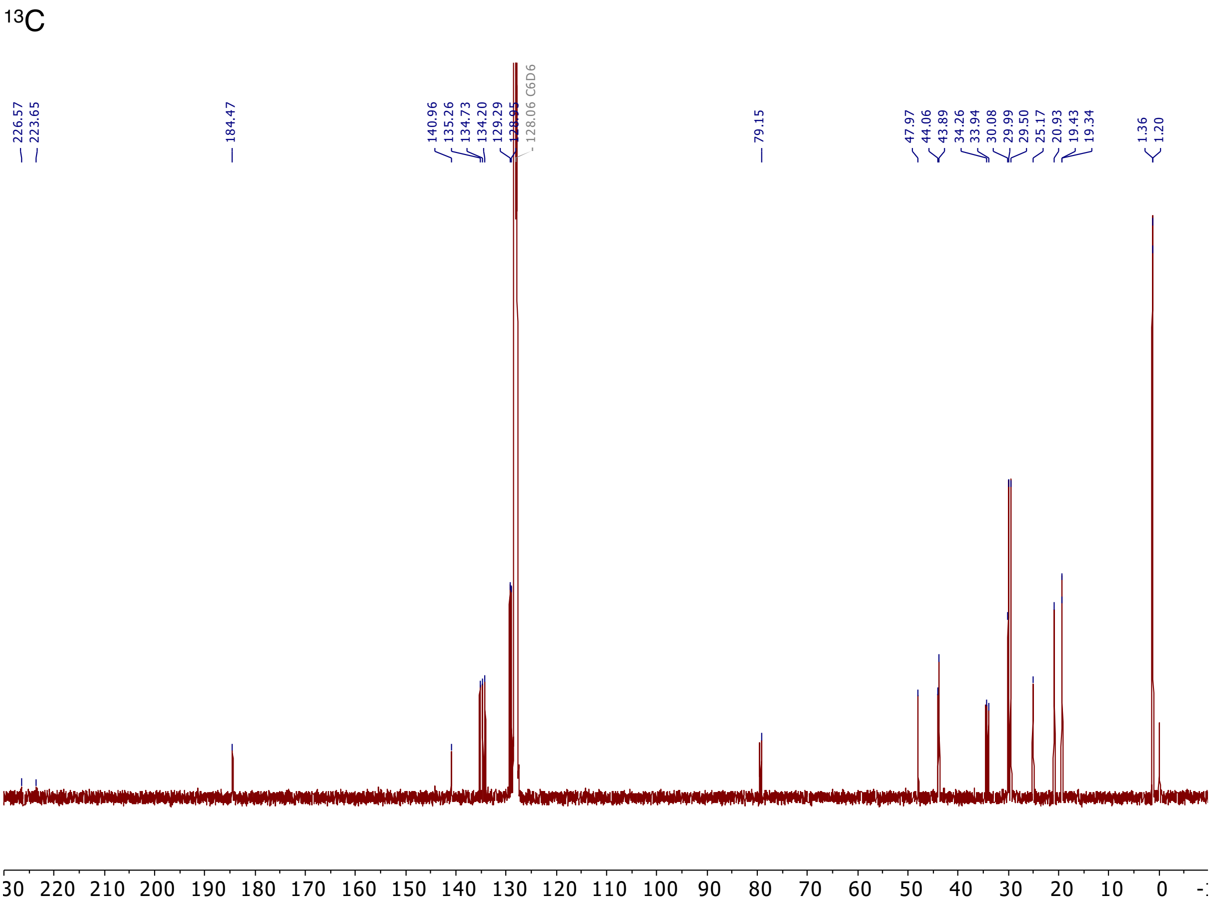
13C
226.57
223.65
184.47
140.96
135.26
134.73
134.20
129.29
128.95
128.06 C6D6
79.15
47.97
44.06
43.89
34.26
33.94
30.08
29.99
29.50
25.17
20.93
19.43
19.34
1.36
1.20

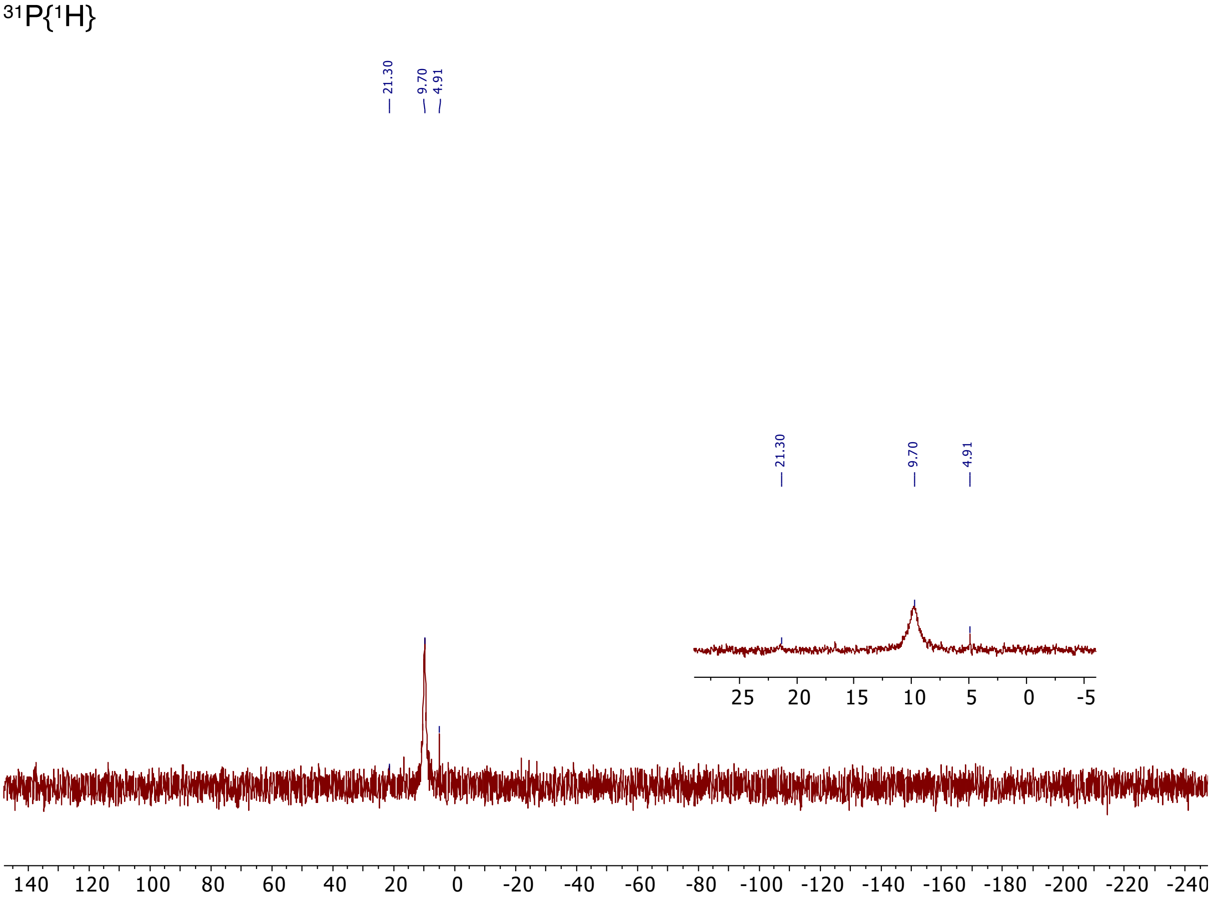
31P{1H}
21.30
9.70
4.91

References

(1) Falconer, R. L.; Nichol, G. S.; Smolyar, I. V.; Cockroft, S. L.; Cowley, M. J. Reversible Reductive Elimination in Aluminum(II) Dihydrides. *Angew. Chemie Int. Ed.* **2021**, *60* (4), 2047–2052. https://doi.org/10.1002/anie.202011418.

(2) Gaussian 16, Revision B.01, M. J. Frisch, G. W. Trucks, H. B. Schlegel, G. E. Scuseria, M. A. Robb, J. R. Cheeseman, G. Scalmani, V. Barone, G. A. Petersson, H. Nakatsuji, X. Li, M. Caricato, A. V. Marenich, J. Bloino, B. G. Janesko, R. Gomperts, B. Mennucci, H. P. Hratchian, J. V. Ortiz, A. F. Izmaylov, J. L. Sonnenberg, D. Williams-Young, F. Ding, F. Lipparini, F. Egidi, J. Goings, B. Peng, A. Petrone, T. Henderson, D. Ranasinghe, V. G. Zakrzewski, J. Gao, N. Rega, G. Zheng, W. Liang, M. Hada, M. Ehara, K. Toyota, R. Fukuda, J. Hasegawa, M. Ishida, T. Nakajima, Y. Honda, O. Kitao, H. Nakai, T. Vreven, K. Throssell, J. A. Montgomery, Jr., J. E. Peralta, F. Ogliaro, M. J. Bearpark, J. J. Heyd, E. N. Brothers, K. N. Kudin, V. N. Staroverov, T. A. Keith, R. Kobayashi, J. Normand, K. Raghavachari, A. P. Rendell, J. C. Burant, S. S. Iyengar, J. Tomasi, M. Cossi, J. M. Millam, M. Klene, C. Adamo, R. Cammi, J. W. Ochterski, R. L. Martin, K. Morokuma, O. Farkas, J. B. Foresman, and D. J. Fox, Gaussian, Inc., Wallingford CT, 2016.

(3) Zhao, Y.; Truhlar, D. *Theo. Chem. Acc.* **2007**, *120* (1-3), 215-241.

(4) Grimme, S.; Antony, J.; Ehrlich, S.; Krieg, H. *J. Chem. Phys.* **2010**, *132* (15), 154104.

(5) a) Weigend, F.; Ahlrichs, R. *Phys. Chem. Chem. Phys.* **2005**, *7* (18), 3297; b) Weigend, F. *Phys Chem. Chem. Phys.* **2006**, *8* (9), 1057.

(6) Marenich, A.; Cramer, C.; Truhlar, D. *J. Phys. Chem. B* **2009**, *113* (18), 6378-6396.

(7) a) Becke, A. *J. Chem. Phys.* **1993**, *98* (7), 5648-5652; b) Lee, C.; Yang, W.; Parr, R. *Phys. Rev. B* **1988**, *37* (2), 785-789; c) Vosko, S.; Wilk, L.; Nusair, M. *Can. J. Phys.* **1980**, *58* (8), 1200-1211; d) Stephens, P.; Devlin, F.; Chabalowski, C.; Frisch, M. *J. Phys. Chem.* **1994**, *98* (45), 11623-11627.

(8) a) McLean, A.; Chandler, G. *J. Chem. Phys.* **1980**, *72* (10), 5639-5648; b) Krishnan, R.; Binkley, J.; Seeger, R.; Pople, J. *J. Chem. Phys.* **1980**, *72* (1), 650-654.

(9) a) Boys, S. F.; Bernardi, F. *Mol. Phys*., **1970**, 19, 553. b) Simon, S.; Duran, M.; Dannenberg, J. J. *J. Chem. Phys.*, **1996**, *105*, 11024-11031.

(10) NBO 6.0, E. D. Glendening, J. K. Badenhoop, A. E. Reed, J. E. Carpenter, C. M. Morales, C. R. Landis, F. Weinhold, Theoretical Chemistry Institute, University of Wisconsin, Madison, 2013.

(11) AIMAll (Version 13.02.26), T. A. Keith, TK Gristmill Software, Overland Park, KS, USA, 2014.

(12) Martin, R. *J. Chem. Phys.* **2003**, *118* (11), 4775-4777.

(13) Gauss, J. *Chem. Phys. Lett*., **1992**, *191*, 614-620.

(14) a) Becke, A.; Edgecombe, K. *J. Chem. Phys.* **1990**, *92* (9), 5397-5403; b) Savin, A.; Becke, A.; Flad, J.; Nesper, R.; Preuss, H.; von Schnering, H. *Angew. Chem. Int. Ed.* 1991, *30* (4), 409-412; c) Savin, A.; Silvi, B.; Colonna, F. *Can. J. Chem.* **1996**, *74* (6), 1088-1096.

(15) Lu, T.; Chen, F. *J. Comput. Chem.* **2011**, *33* (5), 580-592.

(16) Pettersen, E. F.; Goddard, T. D.; Huang, C. C.; Couch G. S.; Greenblatt D.M.; Meng, E. C.; Ferrin T. E. *J. Comput. Chem.* 2004, *13*, 1605-1612.

(17) Chemcraft - graphical software for visualization of quantum chemistry computations. https://www.chemcraftprog.com

(18) Michalski, M; Gordon, A. J.; Berski S. *J. Mol. Model*, **2019**, *25*, 211
